# Cognitive Impairment in Multiple Sclerosis: The Role of Clinical and Sociodemographic Factors ‐ A Systematic Review and Meta‐Analysis

**DOI:** 10.1002/acn3.70172

**Published:** 2025-09-24

**Authors:** Katalin Lugosi, Marie A. Engh, Tamás Kói, Zsolt Molnár, Gábor Csukly, Klaudia Horváth, Emma Hargitai, Péter Hegyi, Zsolt Mezei

**Affiliations:** ^1^ Centre for Translational Medicine Semmelweis University Budapest Hungary; ^2^ Multiple Sclerosis Centre Bajcsy‐Zsilinszky Hospital Budapest Hungary; ^3^ Department of Stochastics Budapest University of Technology and Economics Budapest Hungary; ^4^ Department of Anaesthesiology and Intensive Therapy Semmelweis University Budapest Hungary; ^5^ Department of Anaesthesiology and Intensive Therapy Poznan University of Medical Sciences Poznan Poland; ^6^ Department of Psychiatry and Psychotherapy Semmelweis University Budapest Hungary; ^7^ Institute for Translational Medicine, Medical School, University of Pécs Pécs Hungary; ^8^ Institute of Pancreatic Diseases, Semmelweis University Budapest Hungary; ^9^ Department of Neurology Semmelweis University Budapest Hungary

**Keywords:** cognitive screening, multiple sclerosis, patient characteristics, SDMT

## Abstract

**Objective:**

Cognitive impairment (CI) affects the quality of life in multiple sclerosis (MS). Identifying influencing factors is key to improving CI monitoring. This systematic review and meta‐analysis examines clinical and sociodemographic variables impacting the cognitive screening Symbol Digit Modalities Test (SDMT) performance across MS subtypes, identifying subgroups at greater risk of cognitive impairment.

**Methods:**

Registered on PROSPERO (CRD42023451696), a literature search was conducted on August 6, 2023, using PubMed, Embase, and CENTRAL. Following the PRISMA 2020 guideline, a random‐effects meta‐analysis addressed heterogeneity in correlation and meta‐regression analyses. Multivariable regression model results were qualitatively synthesized (systematic review). The JBI Critical Appraisal Tool assessed bias risk. Primary outcome was SDMT raw scores, with EDSS and disease duration as primary exposures, extended with age, sex, education, depression, fatigue, mobility scores, and treatment as secondary exposures, according to our protocol. Associations were evaluated via univariate study‐level correlations, univariate meta‐regressions, study‐level multivariable regression models, and multivariate meta‐regressions, assessing covariate interdependence. Heterogeneity was quantified with *I* ^2^. Only observational cross‐sectional data (or baseline data from longitudinal studies) were included.

**Results:**

A total of 155 studies with 22,828 patients were analyzed. In mixed and relapsing–remitting MS (RRMS), EDSS was the strongest negative correlate of SDMT (mixed MS: −0.44 CI:[−0.50; −0.36]; RRMS: −0.47 [−0.66; −0.23]). Education showed a moderate positive correlation (mixed MS: 0.31 [0.20; 0.42]; RRMS: 0.32 [−0.02; 0.59]). Due to cross‐sectional design, heterogeneity, and potential aggregation/ecological bias, findings are exploratory.

**Interpretation:**

Poor SDMT performance is mainly driven by physical disability and cognitive reserve (education), modulated by sex, depression, and age, highlighting the need to integrate clinical and sociodemographic data in MS cognitive monitoring.

## 1 Introduction

Multiple sclerosis (MS) is one of the leading causes of disability in young adults [1]. Traditionally, clinical perspectives have emphasized that the degree of *physical impairment*—given the dual main mechanism of “relapse‐associated worsening (RAW)” and “progression independent of relapse activity (PIRA)”—predominantly determines the daily functioning of patients, their ability to work, and consequently, their quality of life [2].

However, seminal studies over the past 20 years have established that *cognitive impairment* is also highly prevalent in MS and can serve not only as a contributing factor but also as a primary cause of overall disability in people with MS (PwMS) [3, 4, 5].

Due to its ease of administration and characteristics such as reliability, validity, predictive validity, sensitivity, and specificity, the Symbol Digit Modalities Test (SDMT) [6] has emerged as the leading neuropsychological assessment tool for MS [3, 7]. As a minimum, the SDMT test is recommended for baseline cognitive screening, as it measures information processing speed—a core feature of overall cognitive impairment—while also assessing connected domains such as attention, working memory, visuomotor coordination, and executive functions [5]. Current recommendations suggest that SDMT‐based cognitive screening is advised at baseline and subsequently annually for patients deemed “clinically stable”. If a “clinically meaningful” change—a 4‐point change or a reduction of 10% on SDMT, or a change with a 0.5 standard deviation, or using Reliable Change Indices change—is detected, a more comprehensive neuropsychological evaluation (including mood assessment) is warranted [5, 7].

Several previous studies have investigated the influence of different patient‐ and disease‐related characteristics on cognitive performance frequently assessed in everyday clinical practice as well (e.g., age, sex, race, education, disease duration, EDSS score). The results suggest that increased EDSS score, older age, lower educational level, and longer disease duration mostly led to a trend toward cognitive dysfunction in the populations studied; although the results are sometimes inconsistent, and the small number of studies, as well as the lack of analysis of the interdependencies between these parameters, often limit the validity of these findings [8, 9, 10, 11].

By identifying key patient and disease characteristics associated with poorer performance on cognitive tests—which may also serve as risk factors for cognitive impairment—we could emphasize intensive neuropsychological monitoring from the outset. This would enable the early detection of subtle, latent signs that may not yet be apparent in the everyday activities of patients, allowing for timely intervention before significant impairment occurs.

Our current comprehensive meta‐analysis seeks to address these considerations. First, we aim to identify the sociodemographic and clinical factors that significantly influence SDMT scores, a test widely recognized as an indicator of overall cognitive function. Following this, we aim to establish a hierarchy of these factors to highlight their relative importance.

## 2 Methods

### 2.1 Study Registration

Our analysis protocol was registered in PROSPERO (international database of prospectively registered systematic reviews; registration ID: CRD42023451696), which we followed without any deviations during the process. We applied the recommendations of the Cochrane Handbook [12] and the Preferred Reporting Items for Systematic Reviews and Meta‐Analyses (PRISMA) 2020 statement [13] (Appendix S1).

### 2.2 Information Sources and Search Strategy

A systematic search was performed in three major databases (Medline—via PubMed, Embase, CENTRAL—The Cochrane Central Register of Controlled Trials) on August 6, 2023 (Appendix S2).

### 2.3 Selection Process

The selection was performed using Endnote 20 (Clarivate Analytics, Philadelphia, PA, USA) software. After automatic and manual removal of duplicates, a selection process was conducted by two independent review authors (Katalin Lugosi and Klaudia Horváth) in two steps (by title and abstract, then by full text), with any disagreements resolved by a third author (Zsolt Mezei). The degree of agreement was quantified by Cohen's kappa statistic.

The “CoCoPop” framework (i.e., condition—context—population) [14] was used to define our selection criteria: the population included adult patients of both sexes (age ≥ 18 years) diagnosed with MS, in the context of their clinical‐sociodemographic features (Expanded Disability Status Scale/EDSS score [15] and disease duration as a minimum), with the condition of SDMT raw score test results (Appendix S3). The initial context of EDSS and disease duration parameters, as a minimum inclusion criterion, was justified by the preliminary literature search, based on feasibility considerations. However, as stated in the PROSPERO protocol, since sufficient data were available, we analyzed other relevant characteristics of the population, such as age, sex, education, depression, fatigue, mobility scores, and treatment. We excluded studies that examined pediatric or pediatric‐onset MS (POMS) populations, smartphone‐based, digital/computerized, or modified/adapted versions of SDMT, and excluded patients who were tested during a relapse, recovery from relapse, or steroid treatment, as these factors could significantly impact cognitive test outcomes [16].

We included only articles that applied the SDMT raw score results—that is, the number of insertions under 90 s—and excluded those that only provided an adjusted “z” or “t” score, as these are already derived values and may distort biased results when examining associations. There was no restriction on the criteria for setting up MS diagnosis, and all MS subtypes [17] were included, except for radiologically isolated syndrome (RIS) patients and “benign MS”. RIS was excluded because not all cases convert to MS, whereas “benign MS” was excluded due to the lack of a universally accepted standardized definition.

A detailed description of all inclusion and exclusion criteria recorded during the selection process can be found in Appendix S4.

All articles included were observational studies. Where a longitudinal study was considered eligible, baseline results were used as cross‐sectional data.

### 2.4 Data Collection Process and Data Items

Data extraction was performed by three reviewers independently (Katalin Lugosi, Klaudia Horváth, and Emma Hargitai) and compared by a fourth author (Zsolt Mezei). Baseline study data (first author, study site, year of publication, study design, study population), clinical‐sociodemographic parameters of the populations (age in years, sex: rate of females, education in years, disease durations in years, EDSS, depression, fatigue, and mobility/gait function scores, disease‐modifying therapy/DMT use), and outcomes (SDMT raw scores, intra‐study direct correlations, and multivariable regression coefficients with the statistical method applied) were extracted into a pre‐designed Excel (Microsoft Corporation, Redmond, Washington, USA) spreadsheet.

### 2.5 Statistical Analysis

Statistical analyses were performed using packages “meta” and “PerformanceAnalytics” of the R statistical software (version 4.1.2). The statistical analyses followed the advice of Harrer et al. [18]. For all statistical analyses, a *p*‐value of < 0.05 was considered significant. All meta‐analyses performed included random effect terms.

When only observational data are available, drawing the right conclusions may be challenging. Confounding can lead to spurious results in univariate analyses. In multivariate analyses, interdependence between predictors makes it difficult to draw the right conclusion. When the meta‐regression is based on aggregated variables, then the results should be interpreted with caution because of the possibility of aggregation bias, as described in section 7.6.2. by Schmid et al. [19]. To obtain the most accurate picture possible, we used four types of analysis separately for mixed MS (including various phenotypes) and RRMS, PPMS, SPMS populations. The first two analyses represent the two key meta‐analyses; the third corresponds to the systematic review component; and the fourth addresses the interdependence among the examined parameters, aiming to assess the reliability of the final results.

- We separately meta‐analyzed the *Pearson and Spearman correlations*. They can be interpreted as *univariate study‐level correlation* measures. We pooled Fisher's z‐transformed correlations using the classical inverse variance approach with REML τ estimator and Hartung‐Knapp adjustment. We visualized the pooled correlations and their 95% confidence intervals in forest plots. Heterogeneity was assessed by calculating the *I* ^2^ measure and its confidence interval and by performing the Cochrane Q test.
- We extracted mean SDMT values along with standard deviation from the studies involved. When only the median and ordered statistics (quartiles, min, max) were available, we used the default method of the metamean () R function to estimate the mean and standard deviation. We performed univariate regression of the SDMT means using the mean or median of the clinical and sociodemographic covariates (*univariate meta‐regressions*). We visualized meta‐regression results in bubble/scatter plots.
- The *study‐level multivariable regression* approaches differed substantially across the studies involved. Several studies selected the covariates involved based on clinical judgment, whereas several studies applied some kind of variable selection. Some of the studies applied methods to detect multi‐collinearity, while others did not. The type of regression tool also differed between studies. For these reasons, a meta‐analysis of the resulting adjusted regression coefficients was not possible. Instead, we created a summary regression table that provided a complete picture of the study‐level multivariable regression results (systematic review part).
- We also performed *meta‐level multivariate regression analyses* of the examined clinical and sociodemographic parameters. We collected covariates that were frequently presented in the studies involved. Then, following the advice of Harrer et al. [18], we used the PerformanceAnalytics R package to assess the pairwise dependency among these covariates. For simplicity, we did not check interdependence among variable triples and quadruples. The resulting correlations between predictors were only simple correlations between the means/medians reported; that is, meta‐weighting was not used in the calculations. However, along with the visualization provided, the results were useful to avoid multicollinearity in the meta‐level regression. Finally, we fitted several multivariate models involving only predictors that were not too strongly correlated. The different runnings served as sensitivity analyses of each other.

For univariate study‐level correlations, meta‐analysis was performed when at least three appropriate studies were available. In cases with fewer studies, or where the correlation method was not Pearson or Spearman, the data were still displayed in the forest plot but were not included in the meta‐analysis. We performed univariate meta‐regression when the number of studies involved was at least eight. However, the Cochrane Handbook [12] does not recommend performing meta‐regression when the number of studies is < 10. For this reason, results based on eight and nine studies should be interpreted with caution.

### 2.6 Risk of Bias Assessment

The risk of bias was assessed using the Joanna Briggs Institute (JBI) Critical Appraisal Tool for Analytical Cross‐Sectional Studies [20, 21] framework by two independent reviewers (KL, EH), with disagreements resolved by a third reviewer (ZMe). Level of evidence rated by The Oxford 2011 Levels of Evidence [22].

## 3 Results

### 3.1 Selection and Study Characteristics

Our search key initially identified 3722 records, and eventually, 155 studies were included in the synthesis (both in the systematic review and in the meta‐analysis).

Although a total of 155 studies were included in the overall analysis, 105 were fit only for univariate meta‐regression analyses, 7 studies examined only univariate study‐level correlations, and 39 studies were suitable for both. Additionally, 4 studies were fit for univariate meta‐regression analyses, univariate study‐level correlations, and multivariable regression models (the multivariable regression models of these 4 articles were also included at the systematic review level). From the 155 included studies, 21 articles examined multiple MS subtypes (i.e., Mixed MS, RRMS, PPMS, SPMS) simultaneously.

**FIGURE 1:**
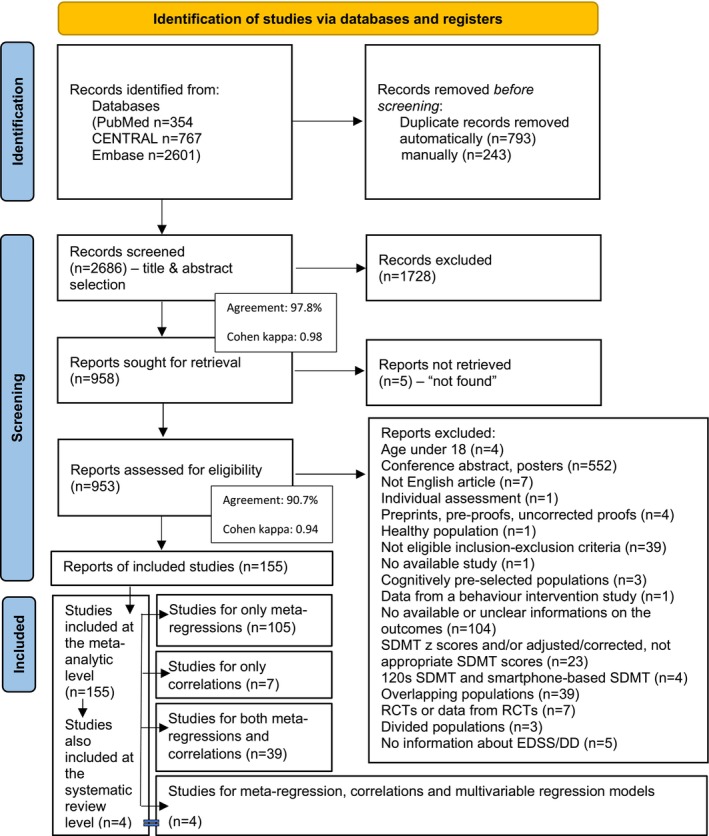
Flow diagram of study identification and selection by PRISMA 2020 with details of the reasons for exclusion (see also Appendix S4 and Appendix S6, Table S1). 155 studies were included at the meta‐analytic level, of which 4 studies were also included at the systematic review level (with statistically non‐analyzable multivariable regression models). From the 155 studies, 105 studies were fit only for univariate meta‐regression analyses, 7 studies examined only univariate study‐level correlations, and 39 studies were suitable for both. Additionally, 4 studies were fit for univariate meta‐regression analyses, univariate study‐level correlations, and multivariable regression models (the multivariable regression models of these 4 articles were included at the systematic review level). From the 155 included studies, 21 articles examined multiple MS subtypes (i.e., Mixed MS, RRMS, PPMS, SPMS) simultaneously. *Cohen kappa*: A statistical measure of inter‐rater agreement that accounts for agreement occurring by chance. It quantifies the consistency between the two independent review authors (Katalin Lugosi and Klaudia Horváth) during study selection. *Agreement*: The degree to which independent review authors made the same inclusion or exclusion decisions during the screening process.

A comprehensive overview of the included studies is provided in Appendix S6, Table S1. Details of the complete selection process are shown in the PRISMA flowchart (Figure 1).

A sufficient amount of aggregate data was available for the following sociodemographic and clinical parameters: age in years, sex: percentage of females, education in years, disease duration in years (“not specified,” “time since diagnosis,” and “time since first symptoms”), EDSS score, depression scores (BDI: Beck Depression Inventory [23], BDI‐II: Beck Depression Inventory‐II [24], BDI‐FS: Beck Depression Inventory‐Fast Screen [25], HADS‐D: Hospital Anxiety and Depression Scale‐Depression score [26]), fatigue scores (FSS: Fatigue Severity Scale [27], MFIS: Modified Fatigue Impact Scale, total scores [28]), Nine‐Hole Peg Test (T9HP) [29] and Timed 25‐Foot Walk Test (T25FW) [30]. The role of “disease‐modifying therapy” (DMT) in influencing cognitive functions was assessed based on the aggregate data from the studied population. Specifically, we evaluated the percentage of patients receiving DMT, distinguishing between “platform” and “highly effective (HET)” treatments. This involved determining what proportion of the population was on DMT (hereafter referred to as “percentage on DMT”) and the respective percentages of those on “platform” versus “high‐efficacy therapy” (hereafter referred to as “percentage on platform” vs. “percentage on HET”). A detailed list of the medications considered “platform” and “highly effective” therapies is provided in the Appendix S1.

Finally, the results of 22,828 MS patients were included in the meta‐analysis. Simultaneously, a systematic review of 505 patients was also performed.

In line with our CoCoPop framework, we report the mean SDMT raw scores as the main cognitive outcome to be interpreted within the context of the relevant and statistically applicable clinical and sociodemographic parameters (EDSS and disease duration as the primary exposures and age, sex, education, depression, fatigue, mobility scores, and treatment as secondary exposures) described above.

A list of references for all included studies is available in the Appendix S5. Baseline characteristics of the studies included are detailed (Appendix S6; Table S1).

In the following, we present our results across four types of analyses at two levels of evidence (first level: *univariate study‐level correlations* and *multivariable study‐level regression models*, and second level: *univariate meta‐regressions*) with *multivariate regressions* based on the pairwise dependency (interdependence) analysis of the examined clinical and sociodemographic covariates.

### 3.2 Meta‐Analysis of Univariate Study‐Level Correlations Stratified by Different MS Subtypes

**FIGURE 2:**
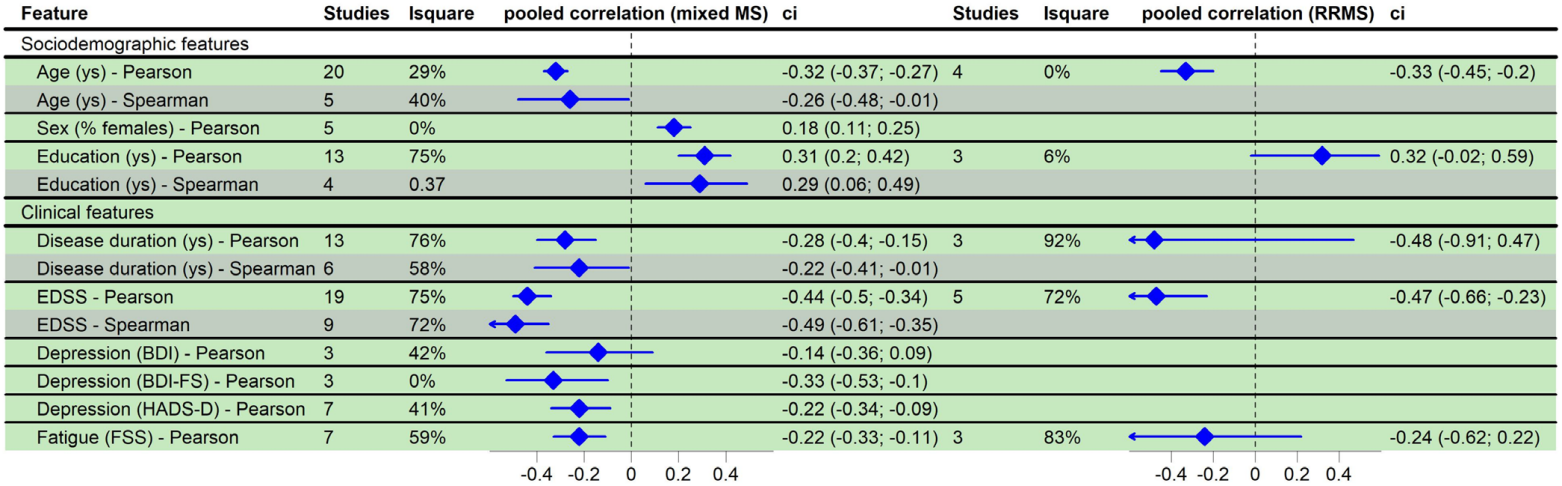
Meta‐analysis results of study‐level correlations. Out of the total 155 included articles, 50 addressed *univariate study‐level correlations*. Among these 50 studies, 1 investigated both Mixed and RRMS populations, 36 focused solely on Mixed populations, and 13 exclusively on RRMS populations. The number of studies presented in Figure 2 reflects the number of studies examining a given parameter within each MS subtype (the subtypes are indicated at the top of the figure). The total count in the figure exceeds 50 because most studies analyzed the *study‐level correlation* between SDMT raw scores and more than one clinical and/or sociodemographic parameter. BDI, beck depression inventory; BDI‐FS, beck depression inventory‐fast screen; CI, confidence interval; EDSS, Expanded Disability Status Scale; FSS, Fatigue Severity Scale; HADS‐D, Hospital Anxiety and Depression Scale‐Depression score; *I* ^2^, level of heterogeneity; *N*, number of the included studies.

For direct intra‐study pooled correlation analyses, based on the available literature, a meta‐analysis could only be performed for the “mixed MS” and RRMS populations. The main findings—taking into account the primary exposures (EDSS and disease duration), the number of the included articles, and the congruence between the levels of evidence—are discussed below, with further details provided in Figure 2.

#### 3.2.1 Mixed MS Populations

For our primary exposures, EDSS and disease duration, both Pearson and Spearman correlations show a highly significant and clearly negative association: higher EDSS score is strongly associated with lower SDMT scores (*Pearson*: −0.44 CI:[−0.50; −0.36], *Spearman*: −0.49 CI:[−0.61; −0.35]) and longer disease duration is correlated with poorer SDMT performance (*Pearson*: −0.28 CI:[−0.40; −0.15], *Spearman*: −0.22 CI:[−0.41; −0.01]).

The female sex has a significantly positive impact on SDMT performance (*Pearson*: 0.18 [0.11; 0.25]), indicating that a higher proportion of females in the population is associated with better SDMT scores.

Pearson correlation analyses for the variable of education yielded highly significant positive correlations (*Pearson*: 0.31 CI: [0.20; 0.42], *Spearman*: 0.29 CI: [0.06; 0.49]), indicating that higher years of education were associated with higher SDMT scores.

For the depression scales—however, with only a few studies were available—a consistent, negative correlation with SDMT scores was observed (BDI: *Pearson*: −0.14 CI:[−0.36; 0.09], BDI‐FS: *Pearson*: −0.33 CI:[−0.53; −0.10], HADS‐D: *Pearson*: −0.22 CI:[−0.34; −0.09]), indicating that a negative trend can be inferred: higher depression scores are associated with lower SDMT scores.

#### 3.2.2 RRMS Populations

Regarding our primary exposures, EDSS showed a significant negative correlation: higher EDSS scores are associated with lower SDMT scores (*Pearson*: −0.47 CI:[−0.66; −0.23]), and disease duration showed a non‐significant negative association: longer disease duration is correlated with poorer SDMT performance (*Pearson*: −0.48 CI:[−0.91; 0.47]).

Education (in years) also demonstrated a nearly significant positive correlation with SDMT scores in the RRMS population (0.32 CI:[−0.02; 0.59]), indicating that higher education is associated with higher SDMT scores. However, this significance level is marginal, based on Pearson correlations, and is derived from only three eligible studies.

A summary panel plot of all the meta‐analyzed direct correlation results is shown in Figure 2. The individual forest plots of the pooled univariate study‐level correlation analyses examined are detailed in Appendix S7.

### 3.3 Results of Univariate Meta‐Regressions Stratified by Different MS Subtypes

Univariate meta‐regression analyses were conducted for the mixed MS and RRMS, PPMS, and SPMS populations. However, for the PPMS and SPMS subgroups, only limited data were available for some parameters, as this was based on a very small number of studies. Therefore, considering the number of included studies, our primary exposures (EDSS and disease duration), the other available parameters, and the congruence across the two levels of evidence, we focus our main findings only on the mixed and RRMS populations, with further details for other MS subgroups and variables provided in Table 1.

**TABLE 1:** Tabular summary of univariate meta‐regression results stratified by different MS subtypes.

	Covariates	Mixed MS	RRMS	PPMS	SPMS
Socio‐demographic features	Age (years)	−0.096 *p*: 0.285 *n*: 164 *N*: 22,211	0.209 *p*: 0.311 *n*: 57 *N*: 4217	−0.408 *p*: 0.777 *n*: 8 *N*: 224	0.11 *p*: 0.679 *n*: 13 *N*: 426
	Sex (% female)	**0.185 *p*: 0.001 *n*: 155** ***N*: 21,320**	0.132 *p*: 0.108 *n*: 52 *N*: 3587	nd	0.13 *p*: 0.496 *n*: 12 *N*: 418
	Education (years)	**2.443 *p* < 0.001 *n*: 67** ***N*: 7722**	**3.636 *p*: < 0.001 *n*: 22** ***N*: 1833**	nd	nd
Clinical features	DD (years)	***−0.278 p*: *0.064 n*:*121*** ***N*: *18,324*** −0.474 *p*: 0.246 *n*: 15^a^ ***N*:** 1218 −0.305 *p*: 0.379 *n*: 9^b^ *N*: 1041	0.044 *p*: 0.913 *n*: 41 *N*: 3085	nd	−0.018 *p*: 0.959 *n*: 10 *N*: 400
	EDSS	**−2.772 *p*: < 0.001 *n*: 151** ***N*: 15,028**	**−3.731 *p*: 0.001 *n*: 57** ***N*: 4217**	0.264 *p*: 0.947 *n*: 8 *N*: 224	−2.367 *p*: 0.555 *n*: 13 *N*: 426
	Depression	**−2.031 *p*: 0.003 *n*: 9** ^c^ ***N*: 1157** **−4.926 *p*: 0.006 *n*: 12** ^d^ ***N*: 1256** **−2.337 *p*: 0.007 *n*: 20** ^e^ ***N*: 3086**	**−3.607 *p*: 0.01 *n*: 8** ^e^ ***N*: 746**	nd	nd
	Fatigue	1.817 *p*: 0.397 *n*: 22^f^ *N*: 2078 −0.168 *p*: 0.297 *n*: 10^g^ *N*: 1097	−0.04 *p*: 0.992 *n*: 10^f^ *N*: 853	nd	nd
	T25FW	**−0.492 *p*: 0.028 *n*: 22** ***N*: 2573**	**−0.818 *p*: 0.016 *n*: 13** ***N*: 911**	nd	nd
	T9HP	−0.669 *p*: 0.218 *n*: 10 *N*: 965	nd	nd	nd
	Treatment	−0.017 *p*: 0.674 *n*: 45^h^ *N*: 9392 −0.011 *p*: 0.86 *n*: 25^i^ *N*: 7139 0.026 *p*: 0.673 *n*: 24^j^ *N*: 7097	0.011 *p*: 0.858 *n*: 24^h^ *N*: 1662 −0.059 *p*: 0.414 *n*: 15^i^ *N*: 896 0.107 *p*: 0.174 *n*: 14^j^ *N*: 854	nd	nd

*Note:* Significant results are highlighted in bold, results close to significance are highlighted in bold and italics.

Abbreviations: DD, disease duration; EDSS, Expanded Disability Status Scale; Edu, education; MS, multiple sclerosis; *N*, number of the included patients; *n*, number of the included studies; nd, no data; *p*, significance level; PPMS, primary progressive multiple sclerosis; RRMS, relapsing–remitting multiple sclerosis; SPMS, secondary progressive multiple sclerosis; T25FW, timed 25‐ft walk test; T9HP, nine‐hole peg test.

^a^ Time since diagnosis.

^b^ Time since first symptom.

^c^ BDI, beck depression inventory.

^d^ BDI‐FS, beck depression inventory fast screen.

^e^ HADS‐D, hospital anxiety and depression scale‐depression score.

^f^ FSS, Fatigue Severity Scale.

^g^ MFIS, Modified Fatigue Impact Scale, total scores.

^h^ % on DMT (disease‐modifying therapy).

^i^ % on platform therapy.

^j^ % on HET (highly effective therapy).

#### 3.3.1 Mixed MS Populations

In mixed MS populations, one of our primary exposures, EDSS, showed the most pronounced and significant negative association with SDMT performance (*b*: −2.772, *p* < 0.001). Our other primary exposure, disease duration, showed a marginally significant negative association with SDMT scores (*b*: −0.278, *p*: 0.064).

Of the additional parameters examined, the severity of depression showed an effect of similar strength to EDSS (as assessed by BDI: *b*: −2.031, *p*: 0.003, BDI‐FS scores: *b*: −4.926, *p*: 0.006; and HADS depression scores: *b*: −2.337 *p*: 0.007).

For education (in years), a strong, significant positive association was observed with SDMT scores (*b*: 2.443, *p* < 0.01).

A strong association was observed for sex (percentage of females), with a significance similar to that of EDSS: the higher the percentage of females in the ‘mixed MS’ populations studied, the higher the SDMT score (*b*: 0.185, *p*: 0.001).

#### 3.3.2 RRMS Populations

In RRMS populations, similar to mixed MS, the EDSS score showed the most pronounced and significant negative association; that is, the more severe the physical impairment, the lower the raw SDMT scores (b: −3.731, *p*: 0.001). The disease duration primary exposure parameter showed a positive, non‐significant association with SDMT scores (b: 0.044, *p*: 0.913), in the opposite direction compared to the mixed MS group.

Among the other parameters studied, also notable is that education emerged as a very strong, significantly negative association parameter with SDMT scores in the RRMS subgroup (b: 3.636, *p* < 0.001), with a relatively moderate number of studies included.

A tabular summary of all the univariate meta‐regression results is shown in Table 1. The individual scatter plots for the examined meta‐regressions are detailed in Appendix S8.

### 3.4 Results of the Systematic Review of Study‐Level Multivariable Regression Models

Given the heterogeneity of variables included in the regression models of the studies and regression model types, we provide *a systematic review* of study‐level multivariable regression analyses.

Four studies conducted multivariate regressions on mixed MS populations. These analyses included linear, stepwise, or logistic regressions with sociodemographic, clinical variables, and other parameters.

Adjustment for age and education was performed in all regression models, with one showing a significant negative coefficient for both parameters. EDSS was the most impactful, with three studies showing significant negative effects, including one with a pronounced impact. Disease duration, sex, and depression showed no significant effects alongside other parameters.

A tabular summary of all the adjusted variables, which were included in the multivariable regression models and adjusted for SDMT as a target parameter, is detailed in Appendix S9, Table S2.

### 3.5 Meta‐Level Multivariate Regression Analyses of the Investigated Clinical and Sociodemographic Factors (Covariates) – Interdependence Analyses

For mixed MS populations, pairwise dependency analysis of the covariates (Appendix S10, Figure S8a) revealed strong positive pairwise linear associations between covariates *age*, *disease duration*, and *EDSS*. For this reason, we fitted three multivariate meta‐regression models. Each model contained the variables *sex* and *education* and one of the three strongly correlated variables.

*Sex* and *education* are always significant, with *education* showing a stronger effect (lower *p*‐value). Age and disease duration are not significant alongside sex and education. EDSS keeps its strong significance in the multivariate regression model, even with the sex and education variables, although education has the strongest effect (for details, see Appendix S11, Table S3).

For RRMS (Appendix S10, Figure S8b), the pairwise correlation analysis revealed that most of the pairwise correlations between the variables *age*, *disease duration*, *EDSS*, and *education* are quite large. We only fitted models containing covariates with not too high pairwise correlations. *Education* and *EDSS* remained consistently strong predictors, while *sex* and *age* had non‐significant or borderline effects. For details, see again Appendix S10, Figure S8, and Appendix S11, Table S3.

### 3.6 Risk of Bias Assessment and Quality of Evidence

On the basis of the JBI Quality Assessment Tool for Analytical Cross‐Sectional Studies, the first four questions indicate a low risk of selection and performance bias, suggesting that the populations in the meta‐analysis were representative. However, the remaining questions highlight a high risk of detection bias, and to a lesser extent, reporting bias, mainly due to variability in how associations were analyzed across studies. This is likely to reflect the heterogeneous reporting of factors influencing cognitive impairment and adjustments in the analyses.

The summary of the assessment of the risk of bias for each included study (listed according to the criteria of the “JBI Quality Assessment Tool for Prevalence Studies”) is detailed in Appendix S12

Rating of the quality of evidence is provided in Appendix S13.

## 4 Discussion

**FIGURE 3:**
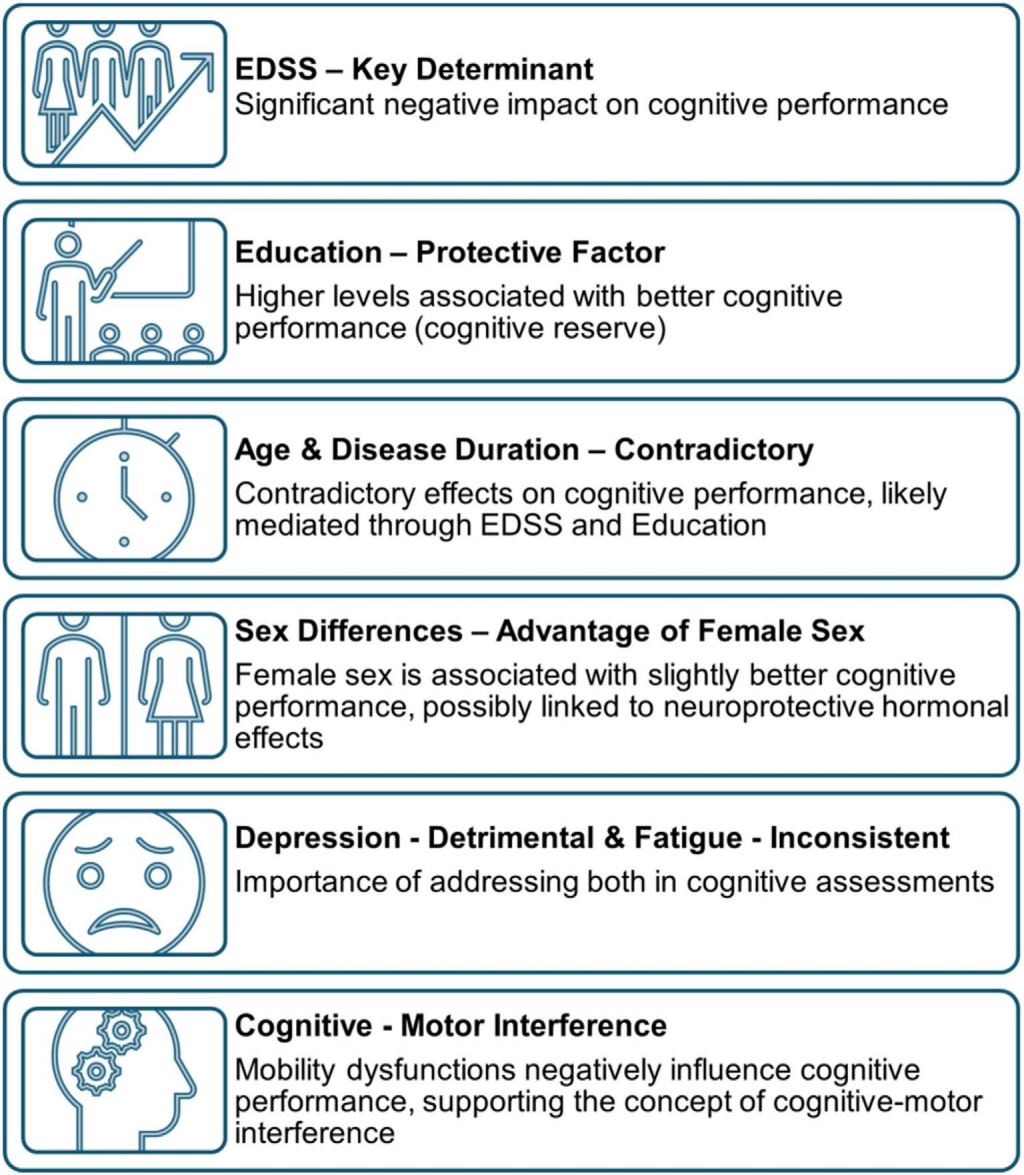
Key findings of our study reveal the multifactorial nature of the relationship between disease‐related and sociodemographic factors and SDMT performance as a key sentinel test for cognitive impairment.

Our study aimed to evaluate the relationship between disease‐related and sociodemographic factors and SDMT performance as a key sentinel test for cognitive impairment. Using robust methods across two levels of evidence, our findings revealed the multifactorial nature of cognitive dysfunction as a significant determinant of overall disability in MS. In the absence of complete evidence for PPMS and SPMS, our conclusions primarily focused on RRMS and mixed MS populations. In this section, our aim is to interpret the hierarchy of all primary and secondary exposure parameters examined in terms of their impact on SDMT performance and to highlight their relative importance based on our findings. Given the inclusion of observational studies (cross‐sectional data), population heterogeneity, and the potentially distorting effects of aggregation/ecological bias, the conclusions should be regarded as primarily exploratory and directional. The key findings supporting this perspective are summarized in Figure 3.

The most significant finding—at all levels of evidence—, was a clear negative impact of *EDSS* on SDMT in the mixed population, a trend also evident in RRMS. In these populations, increased EDSS scores correlated with poorer SDMT performance, supporting its role as a key risk factor of cognitive impairment. In parallel, pairwise dependency analyses in mixed MS populations revealed that age and disease duration had strong positive linear interference, while age positively correlated with EDSS. Educational attainment consistently emerged as a significant predictor for all these parameters in multivariate regression models, indicating that *the negative association between clinical status and cognition becomes more pronounced over time* (*referring to the role of age and disease duration*), *further shaped by the cognitive reserve, which is mainly determined by educational attainment* [31]. This is partially supported by the literature, but the EDSS‐cognition relationship remains debated [4, 32, 33, 34]. Lynch et al. [35] found a definite association between cognitive impairment and EDSS score, but—contrary to the assumption above—it was significant in both the early stage of MS and in those with over 10 years of disease duration. They noted that studies showing strong EDSS‐cognition correlations often used “speeded information processing” tests (e.g., Hohol et al. [36], Sonneville et al. [37])—such as the SDMT—, highlighting that “*patients*' *sensory and motor deficits simply suppress their performance on these tests, leading to poorer scores than their cognitive powers would otherwise yield*”, which also points to the effect of EDSS as a possible explanation. However, Lynch used a battery of more comprehensive tests and identified a correlation of similar magnitude, consistent with our findings.

Our results showed that *age* and *disease duration* had contradictory effects among mixed and RRMS populations. At the meta‐regression level, age was negatively correlated with SDMT scores in mixed MS and positively correlated in RRMS, whereas study‐level correlations and regression models suggested a negative trend in both populations. This discrepancy may be due to the fact that meta‐regression is based on average age in years, which raises the question of whether the observed association reflects different population age averages. Data from countries with higher life expectancy (i.e., higher income, better healthcare, better treatment access)—and thus higher average age—might reverse the association between increased age and improved cognitive performance. However, further investigation is needed to explore this. Disease duration showed a negative trend, but only in study‐level correlations, and was contradictory at the univariate meta‐regression level in RRMS. This complexity of the role of age and disease duration—considering their interdependency and multivariable regression analyses—suggests that *age and disease duration are likely to affect cognitive performance through other parameters* (such as EDSS and education). Lynch et al. [35] found no evidence between cognitive impairment and disease duration. They also questioned the interpretation of the relationship between cognitive impairment and physical disability, as measured by EDSS, as the former is not correlated with disease duration, whereas the latter is. They suggest that in MS, the “inevitable” progression of physical disability—in relation to disease duration and temporal deterioration—allows the association with cognitive decline to persist, despite different dynamics. Our cross‐evidence‐level and multivariable regression analysis appears to support this. Amato et al. [38] also concluded that the negative correlation between clinical status (EDSS) and cognition strengthens over time. This suggests a possible link between disease duration and EDSS, implying that EDSS *might, in fact, operate through the length of time with MS*. A meta‐analysis by Prakash et al. [39] also confirms this interdependence.

*Education* undoubtedly had a positive effect. In RRMS, it showed a significant impact at the meta‐regression level and study‐level multivariable regression models, and marginally significant at the study‐level correlations. In the mixed populations, it was significant at the meta‐regressions and the study‐level correlations, consistent with study‐level multivariable regression models. Overall, *higher educational attainment*—although it appears to be less important compared to EDSS changes—*is associated with higher SDMT scores*. Although there is a lack of detailed studies on years of education and SDMT, the existing literature suggests that education—as one of the main determinants of ‘cognitive reserve’—tends to positively influence cognitive functions in MS [40].

In summary, the highly comprehensive and delicately balanced interplay between EDSS, education, age, and disease duration highlights the complexity of factors influencing SDMT performance. Our study was based on cross‐sectional data; however, a recent longitudinal observational study by Longinetti et al. [41], conducted on a large population‐based sample, showed similar results. They aimed to identify trajectories of SDMT and EDSS and their connections and explore patient characteristics associated with trajectory groups over an 11‐year follow‐up (from DMT initiation) of 1645 RRMS patients, including analyses of conditional probabilities. They found that there is a strong association between processing speed (SDMT) and physical disability (EDSS) trajectories. Based on their results, older age was associated with cognitive impairment at baseline, while female sex and having more than 12 years of education were initially linked to better cognitive trajectories, although these associations weakened after adjusting for MS severity, reinforcing the dominant influence of physical disability on cognitive performance. This is consistent with findings from Foong et al. [42], based on a large longitudinal study of RRMS patients, which identified that higher EDSS, older age, male sex, and depression predicted poorer processing speed (measured by Processing Speed Test/PST—an iPad version of SDMT) over time, while higher educational attainment was protective. Importantly, patients with low baseline cognitive performance and no practice effect were at significantly greater risk of sustained decline. These findings support our cross‐sectional results and highlight the predictive value of EDSS, education, and baseline cognition in cognitive trajectories.

These also raise questions about the reliability of general adjustments for age and education when calculating derived SDMT values (such as *z*‐scores or *t*‐scores), which calls for further research to develop more precise models in this regard.

*Depression* significantly influenced SDMT in mixed MS populations at meta‐regression and study‐level correlation levels, although regression models were less clear. Although this appears to be a straightforward association, caution is needed when interpreting cognitive impairment in patients with concurrent depression due to the potential for pseudo‐dementia, highlighting the importance of parallel testing for comorbid depression when evaluating cognitive functions in MS.

The impact of *fatigue* was less clear due to variability in testing methods and insufficient data. The commonly available FSS test suggested a significant negative effect at study‐level correlations in mixed MS with contradictory findings at the meta‐regression level, and non‐significant negative association in RRMS populations, based on univariate meta‐regressions and study‐level correlations. One possible explanation is a study by Yigit et al. [34] investigating the interplay between depression and fatigue. They found that fatigue, as measured by the FSS score, significantly reduced SDMT scores, whereas depression had no significant effect. However, when both symptoms co‐occurred, a significant decline in information processing speed was observed, particularly on the SDMT from the BiCAMS test. This suggests that depression may “amplify” the impact of fatigue, further worsening cognitive performance. Unfortunately, we lacked sufficient data to perform interdependence and multivariable regression analyses to explore this question further.

*Sex* (the proportion of females in the population) showed a moderate but consistent positive effect on SDMT in mixed MS populations, suggesting that a *higher proportion of females in the MS population tends to correlate with better cognitive performance*. This aligns with literature indicating reduced susceptibility to cognitive decline in females, possibly due to the neuroprotective effect of estrogen (reversing myelin damage [43]) and less gray matter damage [44]. This might explain the protection observed, especially in early‐stage MS, which may also carry the potential risk of menopause [45].

Meta‐regression data alone limit conclusions due to aggregation bias for *T25FW*, *T9HP*, and *treatment* effects. In general, there is weak evidence that T25FW and T9HP tests have a negative influence on SDMT score, with the T25FW test having a significant effect in mixed MS and RRMS, supporting the concept of “cognitive‐motor interference” (CMi) or, alternatively, “dual‐task interference” (DTi) [46] or “cognitive‐postural interference” (CPI) [47], commonly referred to in the literature. They are associated with special neural correlates and the interactions of complex neural networks [48].

In terms of treatments, our results are inconsistent. The literature on DMTs suggests that their cognitive impact varies by specific therapeutic agents, focusing on these effects mainly as secondary or exploratory outcomes [49, 50, 51, 52, 53, 54], with a limited number of studies assessing aggregated effects within broader classifications such as ours [55, 56]. Variability in treatment protocols and patient adherence further complicates this area, underscoring the need for robust clinical trials focusing on cognitive outcomes as primary endpoints.

### 4.1 Strengths and Limitations

The strength of our study lies in the extensive dataset and comprehensive methodological approach, in line with established recommendations, which are critical for the evaluation of future association studies.

However, the main limitation is reliability and the fact that our study is a review of cross‐sectional studies.

We used aggregated data to draw individual conclusions, introducing the “fallacy of the wrong level”, where aggregated relationships may not apply individually. This leads to ecological and aggregation bias, distorting relationships. Interdependence among predictors further complicates regression results, making it difficult to identify the contribution of each parameter to the dependent variable (SDMT scores). To address this, we followed Harrer's guidelines [18], analyzed results across different evidence levels, and conducted interdependence and multivariable regressions. Where study‐level correlations/regressions and meta‐regressions aligned, the effect strength was reliably estimated, ranking of the impact of predictors on cognitive impairment. Thus, the strategy developed to address these confounding factors and the resulting limitations represents a major strength of our study.

Cross‐sectional data do not allow for conclusions about causal inferences or changes over time. Therefore, we can claim to have identified that certain clinical and sociodemographic parameters may act as potential risk factors for poorer cognitive screening performance in MS. Ideally, longitudinal data would enable us to assess how longitudinal cognitive *decline* in MS evolves in relation to these parameters. However, the number of available longitudinal studies was limited, and those that were available varied widely in terms of follow‐up duration and population characteristics, making their integration into a quantitative synthesis infeasible.

### 4.2 Implications for Clinical Practice and Research, and Future Directions

Our study confirms a controversial claim in the international scientific community: the physical status of patients with MS is a strong, independent factor influencing cognitive impairment that also significantly contributes to disability progression. Consequently, maintaining long‐term EDSS stability is critical for clinical practice in MS care in order to preserve the social, familial, and existential integrity of our patients.

A clear implication for research is the multi‐level analysis described, which may serve as a model for future association studies. In MS, a more sophisticated examination of patient‐related factors influencing cognitive impairment should integrate neuropsychological test batteries—for example, BRB‐N, MACFIMS, and BiCAMS [8, 57, 58]—with radiological markers (MRI parameters) and biomarkers, to capture multidomain functional aspects. This approach was not feasible in our study due to insufficient pooled data.

For the future, understanding the interaction of clinical and sociodemographic factors on cognitive performance may improve patient stratification and enable more targeted interventions, translating scientific findings into clinical practice—a top priority for the 21st century [59, 60].

## 5 Conclusion

In multiple sclerosis, poorer performance on SDMT—a sentinel test for cognitive impairment—is shaped by a complex interaction of sociodemographic and clinical factors. Among these, overall physical status (as measured by the EDSS score) and educational attainment were found to be the strongest influencing factors. While preserving patients' physical well‐being and slowing neurological progression remain critical, our findings also emphasize the protective role of higher education, suggesting that cognitive reserve may mitigate the impact of disease‐related cognitive involvement and help maintain cognitive functions essential for preventing disability progression and preserving quality of life in MS.

## Author Contributions

**Katalin Lugosi:** conceptualization, project administration, investigation, data curation, writing – original draft. **Marie A. Engh:** conceptualization, project administration, methodology, writing – review and editing. **Tamás Kói:** conceptualization, statistics, formal analysis, visualization, writing – review and editing. **Zsolt Molnár** and **Gábor Csukly:** conceptualization, writing – review and editing. **Klaudia Horváth** and **Emma Hargitai:** conceptualization, investigation, data curation, writing – review and editing. **Péter Hegyi:** conceptualization, funding acquisition, methodology, writing – review and editing. **Zsolt Mezei:** conceptualization, project administration, writing – review and editing, supervision. All authors certify that they have participated sufficiently in the work to take public responsibility for the content, including participation in the concept, design, analysis, writing, or revision of the manuscript.

## Ethics Statement

The authors have nothing to report. The datasets used in this study can be found in the full‐text articles included in the systematic review and meta‐analysis.

## Conflicts of Interest

Katalin Lugosi received speaking fees from Merck and Novartis, and conference attendance and travel grants from Biogen, Merck, Novartis, and Roche. Klaudia Horváth received conference attendance and travel grants from Merck, Novartis, and Roche. Zsolt Mezei received honoraria for lectures (directly) from Merck and Novartis and conference/travel grants and support (paid to the organizer) from Merck, Novartis, Roche, Sanofi Genzyme, and UCB. Marie A. Engh, Tamás Kói, Zsolt Molnár, Gábor Csukly, Emma Hargitai, and Péter Hegyi declare no conflicts of interest.

## Supporting information


**Data S1:** Supplementary Material.

**Table S1:** Baseline characteristics of the included studies.

## Data Availability

Data in the article will be shared on reasonable request to the corresponding author.

## References

[1] D. S. Goodin , “The Epidemiology of Multiple Sclerosis: Insights to Disease Pathogenesis,” in Handbook of Clinical Neurology, vol. 122 (Elsevier, 2014), 231–266, 10.1016/B978-0-444-52001-2.00010-8. 24507521

[2] F. D. Lublin , D. A. Häring , H. Ganjgahi , et al., “How Patients With Multiple Sclerosis Acquire Disability,” Brain: A Journal of Neurology 145, no. 9 (2022): 3147–3161, 10.1093/brain/awac016. 35104840 PMC9536294

[3] R. H. B. Benedict , M. P. Amato , J. DeLuca , and J. J. G. Geurts , “Cognitive Impairment in Multiple Sclerosis: Clinical Management, MRI, and Therapeutic Avenues,” Lancet. Neurology 19, no. 10 (2020): 860–871, 10.1016/S1474-4422(20)30277-5. 32949546 PMC10011205

[4] N. D. Chiaravalloti and J. DeLuca , “Cognitive Impairment in Multiple Sclerosis,” Lancet. Neurology 7, no. 12 (2008): 1139–1151, 10.1016/S1474-4422(08)70259-X. 19007738

[5] R. Kalb , M. Beier , R. H. Benedict , et al., “Recommendations for Cognitive Screening and Management in Multiple Sclerosis Care,” Multiple Sclerosis (Houndmills, Basingstoke, England) 24, no. 13 (2018): 1665–1680, 10.1177/1352458518803785. 30303036 PMC6238181

[6] A. Smith , Symbol Digit Modalities Test (Western Psychological Services, 1973).

[7] R. H. Benedict , J. DeLuca , G. Phillips , et al., “Validity of the Symbol Digit Modalities Test as a Cognition Performance Outcome Measure for Multiple Sclerosis,” Multiple Sclerosis (Houndmills, Basingstoke, England) 23, no. 5 (2017): 721–733, 10.1177/1352458517690821. 28206827 PMC5405816

[8] S. M. Rao , G. J. Leo , L. Bernardin , and F. Unverzagt , “Cognitive Dysfunction in Multiple Sclerosis. I. Frequency, Patterns, and Prediction,” Neurology 41, no. 5 (1991): 685–691, 10.1212/wnl.41.5.685. 2027484

[9] M. Maurelli , E. Marchioni , R. Cerretano , et al., “Neuropsychological Assessment in MS: Clinical, Neurophysiological and Neuroradiological Relationships,” Acta Neurologica Scandinavica 86, no. 2 (1992): 124–128, 10.1111/j.1600-0404.1992.tb05052.x. 1414220

[10] D. Basci and Z. Tulek , “Assessment of Cognitive Function and Its Predictors in Patients With Multiple Sclerosis: A Case‐Control Study,” Neurological Sciences: Official Journal of the Italian Neurological Society and of the Italian Society of Clinical Neurophysiology 44, no. 3 (2023): 1009–1016, 10.1007/s10072-022-06524-8. 36443543

[11] S. Ozakbas , R. Turkoglu , Y. Tamam , et al., “Prevalence of and Risk Factors for Cognitive Impairment in Patients With Relapsing‐Remitting Multiple Sclerosis: Multi‐Center, Controlled Trial,” Multiple Sclerosis and Related Disorders 22 (2018): 70–76, 10.1016/j.msard.2018.03.009. 29605801

[12] J. P. T. Higgins , J. Thomas , J. Chandler , et al., Cochrane Handbook for Systematic Reviews of Interventions Version 6.3 (Cochrane, 2022).

[13] M. J. Page , J. E. McKenzie , P. M. Bossuyt , et al., “The PRISMA 2020 Statement: An Updated Guideline for Reporting Systematic Reviews,” British Medical Journal 372 (2021): n71, 10.1136/bmj.n71. 33782057 PMC8005924

[14] Z. Munn , C. Stern , E. Aromataris , C. Lockwood , and Z. Jordan , “What Kind of Systematic Review Should I Conduct? A Proposed Typology and Guidance for Systematic Reviewers in the Medical and Health Sciences,” BMC Medical Research Methodology 18, no. 1 (2018): 5, 10.1186/s12874-017-0468-4. 29316881 PMC5761190

[15] J. F. Kurtzke , “Rating Neurologic Impairment in Multiple Sclerosis: An Expanded Disability Status Scale (EDSS),” Neurology 33, no. 11 (1983): 1444–1452, 10.1212/wnl.33.11.1444. 6685237

[16] R. H. Benedict , S. Morrow , J. Rodgers , et al., “Characterizing Cognitive Function During Relapse in Multiple Sclerosis,” Multiple Sclerosis 20, no. 13 (2014): 1745–1752, 10.1177/1352458514533229. 24842959

[17] F. D. Lublin , S. C. Reingold , J. A. Cohen , et al., “Defining the Clinical Course of Multiple Sclerosis: The 2013 Revisions,” Neurology 83, no. 3 (2014): 278–286, 10.1212/WNL.0000000000000560. 24871874 PMC4117366

[18] M. Harrer , P. Cuijpers , T. A. Furukawa , and D. D. Ebert , Doing Meta‐Analysis With R: A Hands‐On Guide (Chapman & Hall/CRC Press, 2021).

[19] C. Schmid , T. Stijnen , and I. White , Handbook of Meta‐Analysis, 1st ed. (CRC Press, 2020), https://www.perlego.com/book/1705208/handbook‐of‐metaanalysis‐pdf.

[20] Joanna Briggs Institute , “Critical Appraisal Tool for Analytical Cross‐Sectional Studies,” 2020, JBI, https://jbi.global/critical‐appraisal‐tools.

[21] L. A. McGuinness and J. P. T. Higgins , “Risk‐Of‐Bias VISualization (Robvis): An R Package and Shiny Web App for Visualizing Risk‐Of‐Bias Assessments,” Research Synthesis Methods 12 (2020): 55–61, 10.1002/jrsm.1411. 32336025

[22] OCEBM Levels of Evidence Working Group , “The Oxford 2011 Levels of Evidence,” Oxford Centre for Evidence‐Based Medicine, http://www.cebm.net/index.aspx?o=5653. *OCEBM Table of Evidence Working Group = Howick, Jeremy, Chalmers, Iain (James Lind Library), Glasziou, Paul, Greenhalgh, Trish, Heneghan, Carl, Liberati, Alessandro, Moschetti, Ivan, Phillips, Bob, Thornton, Hazel, Goddard, Olive, and Hodgkinson, Mary.

[23] A. T. Beck , C. H. Ward , M. Mendelson , J. Mock , and J. Erbaugh , “An Inventory for Measuring Depression,” Archives of General Psychiatry 4, no. 6 (1961): 561–571, 10.1001/archpsyc.1961.01710120031004. 13688369

[24] A. T. Beck , R. A. Steer , and G. K. Brown , Manual for the Beck Depression Inventory‐II (Psychological Corporation, 1996).

[25] A. T. Beck , R. A. Steer , and G. K. Brown , BDI‐FastScreen for Medical Patients: Manual (Psychological Corporation, 2000).

[26] A. S. Zigmond and R. P. Snaith , “The Hospital Anxiety and Depression Scale,” Acta Psychiatrica Scandinavica 67, no. 6 (1983): 361–370, 10.1111/j.1600-0447.1983.tb09716.x. 6880820

[27] L. B. Krupp , N. G. LaRocca , J. Muir‐Nash , and A. D. Steinberg , “The Fatigue Severity Scale: Application to Patients With Multiple Sclerosis and Systemic Lupus Erythematosus,” Archives of Neurology 46, no. 10 (1989): 1121–1123, 10.1001/archneur.1989.00520460115022. 2803071

[28] Multiple Sclerosis Council for Clinical Practice Guidelines , “Fatigue and Multiple Sclerosis: Evidence‐Based Management Strategies for Fatigue in Multiple Sclerosis,” 1998, Paralyzed Veterans of America.

[29] P. Feys , I. Lamers , G. Francis , et al., “The Nine‐Hole Peg Test as a Manual Dexterity Performance Measure for Multiple Sclerosis,” Multiple Sclerosis (Houndmills, Basingstoke, England) 23, no. 5 (2017): 711–720, 10.1177/1352458517690824. 28206826 PMC5405844

[30] R. W. Motl , J. A. Cohen , R. Benedict , et al., “Validity of the Timed 25‐Foot Walk as an Ambulatory Performance Outcome Measure for Multiple Sclerosis,” Multiple Sclerosis (Houndmills, Basingstoke, England) 23, no. 5 (2017): 704–710, 10.1177/1352458517690823. 28206828 PMC5405807

[31] R. Luerding , S. Gebel , E. M. Gebel , S. Schwab‐Malek , and R. Weissert , “Influence of Formal Education on Cognitive Reserve in Patients With Multiple Sclerosis,” Frontiers in Neurology 7 (2016): 46, 10.3389/fneur.2016.00046. 27065941 PMC4809897

[32] S. Demir , “Expanded Disability Status Scale (EDSS) in Multiple Sclerosis,” Cam and Sakura Medical Journal 2, no. 3 (2022): 82–89, 10.4274/csmedj.galenos.2022.2022-11-11.

[33] E. Heled , R. Aloni , and A. Achiron , “Cognitive Functions and Disability Progression in Relapsing‐Remitting Multiple Sclerosis: A Longitudinal Study,” Applied Neuropsychology. Adult 28, no. 2 (2021): 210–219, 10.1080/23279095.2019.1624260. 31204507

[34] P. Yigit , A. Acikgoz , Z. Mehdiyev , A. Dayi , and S. Ozakbas , “The Relationship Between Cognition, Depression, Fatigue, and Disability in Patients With Multiple Sclerosis,” Irish Journal of Medical Science 190 (2021): 1129–1136. 33006048 10.1007/s11845-020-02377-2

[35] S. G. Lynch , B. A. Parmenter , and D. R. Denney , “The Association Between Cognitive Impairment and Physical Disability in Multiple Sclerosis,” Multiple Sclerosis Journal 11, no. 4 (2005): 469–476, 10.1191/1352458505ms1182oa. 16042232

[36] M. J. Hohol , C. R. Guttmann , J. Orav , et al., “Serial Neuropsychological Assessment and Magnetic Resonance Imaging Analysis in Multiple Sclerosis,” Archives of Neurology 54, no. 8 (1997): 1018–1025, 10.1001/archneur.1997.00550200074013. 9267977

[37] L. M. De Sonneville , J. B. Boringa , I. E. Reuling , R. H. Lazeron , H. J. Adèr , and C. H. Polman , “Information Processing Characteristics in Subtypes of Multiple Sclerosis,” Neuropsychologia 40, no. 11 (2002): 1751–1765, 10.1016/s0028-3932(02)00041-6. 12062887

[38] M. P. Amato , V. Zipoli , and E. Portaccio , “Multiple Sclerosis‐Related Cognitive Changes: A Review of Cross‐Sectional and Longitudinal Studies,” Journal of the Neurological Sciences 245, no. 1–2 (2006): 41–46, 10.1016/j.jns.2005.08.019. 16643953

[39] R. S. Prakash , E. M. Snook , J. M. Lewis , R. W. Motl , and A. F. Kramer , “Cognitive Impairments in Relapsing‐Remitting Multiple Sclerosis: A Meta‐Analysis,” Multiple Sclerosis (Houndmills, Basingstoke, England) 14, no. 9 (2008): 1250–1261, 10.1177/1352458508095004. 18701571 PMC2847445

[40] G. Santangelo , M. Altieri , C. Enzinger , A. Gallo , and L. Trojano , “Cognitive Reserve and Neuropsychological Performance in Multiple Sclerosis: A Meta‐Analysis,” Neuropsychology 33, no. 3 (2019): 379–390, 10.1037/neu0000520. 30702305

[41] E. Longinetti , S. Englund , J. Burman , et al., “Trajectories of Cognitive Processing Speed and Physical Disability Over 11 Years Following Initiation of a First Multiple Sclerosis Disease‐Modulating Therapy,” Journal of Neurology, Neurosurgery, and Psychiatry 95, no. 2 (2024): 134–141, 10.1136/jnnp-2023-331784. 37558400 PMC10850621

[42] Y. C. Foong , D. Merlo , M. Gresle , et al., “Longitudinal Trajectories of Digital Cognitive Biomarkers for Multiple Sclerosis,” Annals of Clinical and Translational Neurology 12, no. 4 (2025): 842–850, 10.1002/acn3.70015. 40007145 PMC12040512

[43] C. E. Meyer , A. W. Smith , A. A. Padilla‐Requerey , et al., “Neuroprotection in Cerebral Cortex Induced by the Pregnancy Hormone Estriol,” Laboratory Investigation; A Journal of Technical Methods and Pathology 103, no. 8 (2023): 100189, 10.1016/j.labinv.2023.100189. 37245852 PMC11927460

[44] R. R. Voskuhl , K. Patel , F. Paul , et al., “Sex Differences in Brain Atrophy in Multiple Sclerosis,” Biology of Sex Differences 11, no. 1 (2020): 49, 10.1186/s13293-020-00326-3. 32859258 PMC7456053

[45] M. Avila , A. Bansal , J. Culberson , and A. N. Peiris , “The Role of Sex Hormones in Multiple Sclerosis,” European Neurology 80, no. 1–2 (2018): 93–99, 10.1159/000494262. 30343306

[46] G. Coghe , G. Fenu , L. Lorefice , et al., “Association Between Brain Atrophy and Cognitive Motor Interference in Multiple Sclerosis,” Multiple Sclerosis and Related Disorders 25 (2018): 208–211, 10.1016/j.msard.2018.07.045. 30103173

[47] S. Ruggieri , F. Fanelli , L. Castelli , N. Petsas , L. De Giglio , and L. Prosperini , “Lesion Symptom Map of Cognitive‐Postural Interference in Multiple Sclerosis,” Multiple Sclerosis (Houndmills, Basingstoke, England) 24, no. 5 (2018): 653–662, 10.1177/1352458517701313. 28337941 PMC5946662

[48] C. Leone , P. Feys , L. Moumdjian , E. D'Amico , M. Zappia , and F. Patti , “Cognitive‐Motor Dual‐Task Interference: A Systematic Review of Neural Correlates,” Neuroscience and Biobehavioral Reviews 75 (2017): 348–360, 10.1016/j.neubiorev.2017.01.010. 28104413

[49] E. Riepl , S. Pfeuffer , T. Ruck , et al., “Alemtuzumab Improves Cognitive Processing Speed in Active Multiple Sclerosis‐A Longitudinal Observational Study,” Frontiers in Neurology 8 (2018): 730, 10.3389/fneur.2017.00730. 29387035 PMC5775967

[50] M. Gudesblatt , K. Wissemann , M. Zarif , et al., “Improvement in Cognitive Function as Measured by NeuroTrax in Patients With Relapsing Multiple Sclerosis Treated With Natalizumab: A 2‐Year Retrospective Analysis,” CNS Drugs 32, no. 12 (2018): 1173–1181, 10.1007/s40263-018-0553-1. 30143944 PMC6280854

[51] R. H. Benedict , J. de Seze , and S. L. Hauser , “Impact of Ocrelizumab on Cognition in Patients at Increased Risk of Developing Progressive Disease (abstract DX67),” 2018, Presented at the Consortium of Multiple Sclerosis Centers Annual Meeting, Nashville, TN, May 30–June 2, 2018.

[52] V. Zipoli , P. Tortorella , and B. Goretti , “Effect of Delayed‐Release Dimethyl Fumarate on Cognition in Italian Patients With Relapsing‐Remitting Multiple Sclerosis: The Phase 4 StarTec Study (abstract P457),” 2018, Presented at the 34th Congress of the European Committee for Treatment and Research in Multiple Sclerosis, Berlin, Germany, October 10–12, 2018.

[53] B. A. Cree , M. D. Goldman , J. R. Corboy , et al., “Effect of Fingolimod on Functional Disability, Cognition, and Quality of Life Outcomes Versus Glatiramer Acetate in Relapsing‐Remitting Multiple Sclerosis Patients: Results From the ASSESS Study (abstract EPO1230),” 2019, Presented at the 5th Congress of the European Academy of Neurology, Oslo, Norway, June 29–July 2, 2019.

[54] T. Sprenger , L. Kappos , E. W. Radue , et al., “Association of Brain Volume Loss and Long‐Term Disability Outcomes in Patients With Multiple Sclerosis Treated With Teriflunomide,” Multiple Sclerosis (Houndmills, Basingstoke, England) 26, no. 10 (2020): 1207–1216, 10.1177/1352458519855722. 31198103 PMC7493202

[55] A. Aboseif , M. Amin , J. Bena , K. Nakamura , G. Macaron , and D. Ontaneda , “Association Between Disease‐Modifying Therapy and Information Processing Speed in Multiple Sclerosis,” International Journal of MS Care 26, no. 3 (2024): 91–97, 10.7224/1537-2073.2023-010. 38765300 PMC11096850

[56] M. Gonzalez‐Lorenzo , B. Ridley , S. Minozzi , et al., “Immunomodulators and Immunosuppressants for Relapsing‐Remitting Multiple Sclerosis: A Network Meta‐Analysis,” Cochrane Database of Systematic Reviews 1, no. 1 (2024): CD011381, 10.1002/14651858.CD011381.pub3. 38174776 PMC10765473

[57] R. H. Benedict , J. S. Fischer , C. J. Archibald , et al., “Minimal Neuropsychological Assessment of MS Patients: A Consensus Approach,” Clinical Neuropsychologist 16, no. 3 (2002): 381–397, 10.1076/clin.16.3.381.13859. 12607150

[58] D. W. Langdon , M. P. Amato , J. Boringa , et al., “Recommendations for a Brief International Cognitive Assessment for Multiple Sclerosis (BICAMS),” Multiple Sclerosis 18, no. 6 (2012): 891–898, 10.1177/1352458511431076. 22190573 PMC3546642

[59] P. Hegyi , B. Erőss , F. Izbéki , A. Párniczky , and A. Szentesi , “Accelerating the Translational Medicine Cycle: The Academia Europaea Pilot,” Nature Medicine 27, no. 8 (2021): 1317–1319, 10.1038/s41591-021-01458-8. 34312557

[60] P. Hegyi , O. H. Petersen , S. Holgate , et al., “Academia Europaea Position Paper on Translational Medicine: The Cycle Model for Translating Scientific Results Into Community Benefits,” Journal of Clinical Medicine 9, no. 5 (2020): 1532, 10.3390/jcm9051532. 32438747 PMC7290380

