# Supplementary material for: Cognitive Impairment in Multiple Sclerosis: The Role of Clinical and Sociodemographic Factors ‐ A Systematic Review and Meta‐Analysis
Source: Ann Clin Transl Neurol. 2025 Sep 24;13(1):58–70. doi: 10.1002/acn3.70172 (PMC12790164; doi:10.1002/acn3.70172)
Supplement: Supplementary file 1 — Data S1: Supplementary Material. [file ACN3-13-58-s002.docx]

**SUPPLEMENTARY MATERIAL**

**Katalin Lugosi, Zsolt Mezei et al.: Cognitive Impairment in Multiple Sclerosis: The Role of Clinical and Sociodemographic Factors**

- **A Systematic Review and Meta-analysis**

**Appendix 1:** PRISMA 2020 Checklist

**Appendix 2:** Search keys

**Appendix 3:** Clinical question and CoCoPop framework for literature searching

**Appendix 4:** Details of the selection and inclusion-exclusion criteria during the selection process

**Appendix 5:** List of references for all included studies

**Appendix 6, Supplemental table 1:** Baseline characteristics of the included studies (attached).

**Appendix 7, Supplemental figure 1-3:** Individual forest plots of study-level correlations stratified by different MS subtypes („Mixed MS”, RRMS)

**Appendix 8, Supplemental figure 4-7:** Individual scatter plots of meta-regressions stratified by different MS subtypes („Mixed MS”, RRMS)

**Appendix 9, Supplemental table 2a-b:** Summary table of the study-level multivariate regression analyses (systematic review part)

**Appendix 10, Supplemental figure 8a-b:** Pairwise dependency analysis of the covariates

**Appendix 11, Supplemental table 3:** Multivariable regression models based on the results of the pairwise analysis of the covariates

**Appendix 12, Supplemental figure 9-10:** Assessment of risk of bias for each included study (listed in accordance with the „JBI Quality Assessment Tool for Analytical Cross-Sectional Studies” criteria)

**Appendix 13:** Ratings of the quality of the evidence (the [Oxford Centre for Evidence-based Medicine](https://www.cebm.net/index.aspx-3Fo-3D5653) for ratings of individual studies)

**Appendix 1: PRISMA 2020 Checklist**

| **Section and Topic** | **Item #** | **Checklist item** | **Location where item is reported** |
| --- | --- | --- | --- |
| **TITLE** | | |  |
| Title | 1 | Identify the report as a systematic review. | Page 1 |
| **ABSTRACT** | | |  |
| Abstract | 2 | See the PRISMA 2020 for Abstracts checklist. | Page 3 |
| **INTRODUCTION** | | |  |
| Rationale | 3 | Describe the rationale for the review in the context of existing knowledge. | Page 4 |
| Objectives | 4 | Provide an explicit statement of the objective(s) or question(s) the review addresses. | Page 4 |
| **METHODS** | | |  |
| Eligibility criteria | 5 | Specify the inclusion and exclusion criteria for the review and how studies were grouped for the syntheses. | Page 5, Suppl Mat App 3-4 |
| Information sources | 6 | Specify all databases, registers, websites, organisations, reference lists and other sources searched or consulted to identify studies. Specify the date when each source was last searched or consulted. | Page 5, Suppl Mat App 2 |
| Search strategy | 7 | Present the full search strategies for all databases, registers and websites, including any filters and limits used. | Page 5, Suppl Mat App 2 |
| Selection process | 8 | Specify the methods used to decide whether a study met the inclusion criteria of the review, including how many reviewers screened each record and each report retrieved, whether they worked independently, and if applicable, details of automation tools used in the process. | Page 5-6, Suppl Mat, App 4 |
| Data collection process | 9 | Specify the methods used to collect data from reports, including how many reviewers collected data from each report, whether they worked independently, any processes for obtaining or confirming data from study investigators, and if applicable, details of automation tools used in the process. | Page 6 |
| Data items | 10a | List and define all outcomes for which data were sought. Specify whether all results that were compatible with each outcome domain in each study were sought (e.g. for all measures, time points, analyses), and if not, the methods used to decide which results to collect. | Page 6 |
|  | 10b | List and define all other variables for which data were sought (e.g. participant and intervention characteristics, funding sources). Describe any assumptions made about any missing or unclear information. | Page 6 |
| Study risk of bias assessment | 11 | Specify the methods used to assess risk of bias in the included studies, including details of the tool(s) used, how many reviewers assessed each study and whether they worked independently, and if applicable, details of automation tools used in the process. | Page 7 |
| Effect measures | 12 | Specify for each outcome the effect measure(s) (e.g. risk ratio, mean difference) used in the synthesis or presentation of results. | Page 6-7 |
| Synthesis methods | 13a | Describe the processes used to decide which studies were eligible for each synthesis (e.g. tabulating the study intervention characteristics and comparing against the planned groups for each synthesis (item #5)). | Page 6-7 |
|  | 13b | Describe any methods required to prepare the data for presentation or synthesis, such as handling of missing summary statistics, or data conversions. | Page 6-7 |
|  | 13c | Describe any methods used to tabulate or visually display results of individual studies and syntheses. | Page 6-7 |
|  | 13d | Describe any methods used to synthesize results and provide a rationale for the choice(s). If meta-analysis was performed, describe the model(s), method(s) to identify the presence and extent of statistical heterogeneity, and software package(s) used. | Page 6-7 |
|  | 13e | Describe any methods used to explore possible causes of heterogeneity among study results (e.g. subgroup analysis, meta-regression). | Page 6-7 |
|  | 13f | Describe any sensitivity analyses conducted to assess robustness of the synthesized results. | Page 6-7 |
| Reporting bias assessment | 14 | Describe any methods used to assess risk of bias due to missing results in a synthesis (arising from reporting biases). | Page 7 |
| Certainty assessment | 15 | Describe any methods used to assess certainty (or confidence) in the body of evidence for an outcome. | Page 7 |
| **RESULTS** | | |  |
| Study selection | 16a | Describe the results of the search and selection process, from the number of records identified in the search to the number of studies included in the review, ideally using a flow diagram. | Page 7-8 |
|  | 16b | Cite studies that might appear to meet the inclusion criteria, but which were excluded, and explain why they were excluded. | Page 7-8, Suppl Mat App 5-6 |
| Study characteristics | 17 | Cite each included study and present its characteristics. | Suppl Mat App. 5-6 |
| Risk of bias in studies | 18 | Present assessments of risk of bias for each included study. | Page 11-12, Suppl Mat App 12 |
| Results of individual studies | 19 | For all outcomes, present, for each study: (a) summary statistics for each group (where appropriate) and (b) an effect estimate and its precision (e.g. confidence/credible interval), ideally using structured tables or plots. | Suppl Mat App 7-9 |
| Results of syntheses | 20a | For each synthesis, briefly summarise the characteristics and risk of bias among contributing studies. | Page 9-12 |
|  | 20b | Present results of all statistical syntheses conducted. If meta-analysis was done, present for each the summary estimate and its precision (e.g. confidence/credible interval) and measures of statistical heterogeneity. If comparing groups, describe the direction of the effect. | Page 9-12, Suppl Mat App 10-11 |
|  | 20c | Present results of all investigations of possible causes of heterogeneity among study results. | Page 9-12, Suppl Mat App 10-11 |
|  | 20d | Present results of all sensitivity analyses conducted to assess the robustness of the synthesized results. | Page 9-12, Suppl Mat App 10-11 |
| Reporting biases | 21 | Present assessments of risk of bias due to missing results (arising from reporting biases) for each synthesis assessed. | Page 11-12, Suppl Mat App 12 |
| Certainty of evidence | 22 | Present assessments of certainty (or confidence) in the body of evidence for each outcome assessed. | Page 11-12, Suppl Mat App 12 |
| **DISCUSSION** | | |  |
| Discussion | 23a | Provide a general interpretation of the results in the context of other evidence. | Page 12-15 |
|  | 23b | Discuss any limitations of the evidence included in the review. | Page 15 |
|  | 23c | Discuss any limitations of the review processes used. | Page 15 |
|  | 23d | Discuss implications of the results for practice, policy, and future research. | Page 15-16 |
| **OTHER INFORMATION** | | |  |
| Registration and protocol | 24a | Provide registration information for the review, including register name and registration number, or state that the review was not registered. | Page 5 |
|  | 24b | Indicate where the review protocol can be accessed, or state that a protocol was not prepared. | Page 5 |
|  | 24c | Describe and explain any amendments to information provided at registration or in the protocol. | Page 5 |
| Support | 25 | Describe sources of financial or non-financial support for the review, and the role of the funders or sponsors in the review. | Page 18 |
| Competing interests | 26 | Declare any competing interests of review authors. | Page 17 |
| Availability of data, code and other materials | 27 | Report which of the following are publicly available and where they can be found: template data collection forms; data extracted from included studies; data used for all analyses; analytic code; any other materials used in the review. | Page 6, 18 |

**Appendix 2: Search keys**

Search engines: MEDLINE (via PubMed), CENTRAL (The Cochrane Central Register of Controlled Trials), Embase

• Date of search: 6th August 2023

• Restrictions: English records

**MEDLINE (via PubMed):**

(SDMT OR „Symbol Digit Modalities Test” OR „Symbol Digits Modalities Test” OR „Symbol Digits Modality Test” OR „Symbol Digit Modality Test” OR „Single Digit Modalities Test” OR „Single Digits Modalities Test” OR „Single Digit Modality Test” OR „Single Digits Modality Test”) AND multiple sclerosis

**Cochrane Library (CENTRAL):**

(SDMT OR „Symbol Digit Modalities Test” OR „Symbol Digits Modalities Test” OR „Symbol Digits Modality Test” OR „Symbol Digit Modality Test” OR „Single Digit Modalities Test” OR „Single Digits Modalities Test” OR „Single Digit Modality Test” OR „Single Digits Modality Test”) AND multiple sclerosis

**Embase:**

(SDMT OR „Symbol Digit Modalities Test” OR „Symbol Digits Modalities Test” OR „Symbol Digits Modality Test” OR „Symbol Digit Modality Test” OR „Single Digit Modalities Test” OR „Single Digits Modalities Test” OR „Single Digit Modality Test” OR „Single Digits Modality Test”) AND multiple sclerosis

**Appendix 3: Clinical question and CoCoPop framework for literature searching**

**Q:** What is the mean cognitive screening test result among the MS population? Does it differ based on EDSS score and disease duration?

**Co (condition):** Cognitive screening test result (SDMT raw scores)

**Co (context):** EDSS score, disease duration (DD) – (at least)

**Pop (population):** MS population

**Exposures:**

Exposure of interest:

As a minimum, EDSS score and/or data about disease duration

**Main outcomes:**

Mean SDMT raw scores (i.e., not derived/adjusted value, but the number of the insertions in 90 seconds)

**Appendix 4: Details of the selection and inclusion-exclusion criteria during the selection process**

**Inclusion criteria:**

- Adult (≥18years) MS patients of both sexes who have undergone SDMT testing and have EDSS score and/or data about disease duration at the same time
- Study designs: observational studies (both descriptive and analytical studies: case-controls, cross-sectionals and cohort studies - longitudinal/follow-ups: if it provides outcome data [SDMT raw scores] and baseline characteristics [clinical and sociodemographic parameters] paralelly at the same time for measuring)

**Exclusion criteria:**

- Patients <18years (pediatric population) and pediatric-onset MS (POMS) - except for studies comparing pediatric and adult MS, in which case we used adults’ data
- Study designs: Experimental studies:RCTs and non-RCTs, from the observational studies: case reports, case series and reviews, poster sessions, e-presentation sessions, corrigendums, supplements, book chapters, study protocols, conference/meeting abstracts, posters, commentaries, erratums, meeting papers, letters to the editor, preprints, pre-proofs, uncorrected proofs

NOTES:

- Due to the no restricting to MS subtype, the commonly referred MS population or „PwMS” („People with Multiple Sclerosis”) population was also included (if not concretized even in the full-text selection, these patients were put in the "mixed MS population" group)
- If it is only mentioned in general terms that a "cognitive test", "cognitive testing" or "neuropsychological testing" or „neuropsychological scores” or „neuropsychological assessment was taken” was carried out, this could be supported at this phase, as in many cases, only the full text gives the exact name of the tests being tested
- At this phase, BiCAMS, MACFIMS, and BRB (or BRB-N) were also included because SDMT is a part of these neuropsychological test batteries
- „MS diagnosis with…symptoms” – it is a restriction, excluded
- Smartphone-based or digital/computerized/modified/electronic/fMRI-adapted versions of the SDMT tests were not acceptable, but if a computerized test was compared with an original one, it could be included
- An article that used a single treatment for all patients - either a therapeutic drug treatment or a cognitive rehabilitation treatment - with the aim of testing the effect of the drug on cognitive function (or e.g., the effect of smoking on cognitive functions) was not eligible, as this would have biased the results
- Also, a comparative study where one group received one therapy and another group received another therapy (drug or rehabilitation therapy) to assess cognitive function was not included
- A divided population based on any aspects (e.g., patients with dyslipidemia - without dyslipidemia, fallers – non-fallers, employed – unemployed, cognitively impaired – cognitively not impaired, fatigued – non-fatigued, etc.) was not eligible
- RIS („Radiologically Isolated Syndrome”) population and „possible MS” patients must have been excluded
- Chart reviews were eligible
- Already the terms "chronic progressive MS", „progressive MS”, "relapsing MS (RMS)" and "relapsing progressive MS (RPMS)" mentioned in the title/abstract were not eligible; only the specific MS: CIS, RRMS, PPMS, SPMS, or "commonly referred to" was eligible
- NEDA 3/4 patients – as it is a restriction – were excluded
- If the study had already screened the population with a cognitive test beforehand and those with a lower score on that test were excluded, then those articles were dropped (although sometimes, it was only revealed at the full-text selection phase)
- „Benign MS” („BMS”) population was excluded
- Articles where cognitive testing was done during relapse and/or steroid treatment were excluded (at least one month had to elapse between relapse/steroid and cognitive testing)
- The „measurement of information processing speed” was also acceptable at this phase, because SDMT measures IPS
- Adjusted scores (z-score, t-score) of SDMT were not eligible
- Oral and written versions of SDMT were also eligible – if both were available, oral (verbal) was chosen
- Baseline data were used in case of longitudinal/follow-up studies
- In the case of overlapping populations, the primary criterion for selection was to include the highest level of association analysis (e.g. if one study included only mean demographic-clinical and SDMT values, and the overlapping population included correlation or even regression analysis, the latter was retained); if the overlap articles agreed on this requirement, the secondary criterion was the highest possible sample size
- **„First-line treatments” ( „platform” therapies in treatment analyses): interferons, teriflunomide, dimethyl-fumarate, glatiramer-acetate**
- **„Second-line treatments” („HET”: „High Efficacy Treatment” therapies in treatment analyses): fingolimod, natalizumab, ocrelizumab, alemtuzumab, cladribin, siponimod, ponesimod, ofatumumab, ozanimod, mitoxantron, daclizumab, rituximab, and other immunomodulatory therapies used in rheumatology that are not approved for the treatment of MS: mycophenolate-mofetil, azathioprin, methotrexate**

**Appendix 5: List of references for all included studies**

1. Abasıyanık, Z., Özdoğar, A. T., Sağıcı, Ö., Kahraman, T., Baba, C., Ertekin, Ö., & Özakbaş, S. (2020). Explanatory factors of balance confidence in persons with multiple sclerosis: Beyond the physical functions. *Multiple sclerosis and related disorders*, *43*, 102239. <https://doi.org/10.1016/j.msard.2020.102239>
2. Abdel Naseer, M., Fathi, S., Roshdy, N. K., Labib, D. M., Khalil, D. H., Ibrahim, W., & Magdy, R. (2019). Cognitive and physical disability in Egyptian patients with multiple sclerosis: genetic and optical coherence tomography study. *Neurological research*, *41*(7), 644–651. <https://doi.org/10.1080/01616412.2019.1609203>
3. Abel, S., Vavasour, I., Lee, L. E., Johnson, P., Ristow, S., Ackermans, N., Chan, J., Cross, H., Laule, C., Dvorak, A., Schabas, A., Hernández-Torres, E., Tam, R., Kuan, A. J., Morrow, S. A., Wilken, J., Rauscher, A., Bhan, V., Sayao, A. L., Devonshire, V., … Kolind, S. H. (2020). Associations Between Findings From Myelin Water Imaging and Cognitive Performance Among Individuals With Multiple Sclerosis. *JAMA network open*, *3*(9), e2014220. <https://doi.org/10.1001/jamanetworkopen.2020.14220>
4. Akatani, R., Chihara, N., Tachibana, H., Koto, S., Kowa, H., Kanda, F., Matsumoto, R., & Toda, T. (2019). Validation of the Guy's Neurological Disability Scale as a screening tool for cognitive impairment in multiple sclerosis. *Multiple sclerosis and related disorders*, *35*, 272–275. <https://doi.org/10.1016/j.msard.2019.08.012>
5. Akbar, N., Honarmand, K., Kou, N., & Feinstein, A. (2011). Validity of a computerized version of the symbol digit modalities test in multiple sclerosis. *Journal of neurology*, *258*(3), 373–379. <https://doi.org/10.1007/s00415-010-5760-8>
6. Andersen, K. W., Lasič, S., Lundell, H., Nilsson, M., Topgaard, D., Sellebjerg, F., Szczepankiewicz, F., Siebner, H. R., Blinkenberg, M., & Dyrby, T. B. (2020). Disentangling white-matter damage from physiological fibre orientation dispersion in multiple sclerosis. *Brain communications*, *2*(2), fcaa077. <https://doi.org/10.1093/braincomms/fcaa077>
7. Arnett, P. A., Cadden, M., Roman, C. A. F., Guty, E., Riegler, K., & Thomas, G. (2022). Sensory-Motor and Affective-Fatigue Factors are Associated with Symbol Digit Performance in Multiple Sclerosis. *Journal of the International Neuropsychological Society : JINS*, *28*(4), 362–370. <https://doi.org/10.1017/S1355617721000540>
8. Baijot, J., Van Laethem, D., Denissen, S., Costers, L., Cambron, M., D'Haeseleer, M., D'hooghe, M. B., Vanbinst, A. M., De Mey, J., Nagels, G., & Van Schependom, J. (2022). Radial diffusivity reflects general decline rather than specific cognitive deterioration in multiple sclerosis. *Scientific reports*, *12*(1), 21771. <https://doi.org/10.1038/s41598-022-26204-z>
9. Balloff, C., Penner, I. K., Ma, M., Georgiades, I., Scala, L., Troullinakis, N., Graf, J., Kremer, D., Aktas, O., Hartung, H. P., Meuth, S. G., Schnitzler, A., Groiss, S. J., & Albrecht, P. (2022). The degree of cortical plasticity correlates with cognitive performance in patients with Multiple Sclerosis. *Brain stimulation*, *15*(2), 403–413. <https://doi.org/10.1016/j.brs.2022.02.007>
10. Batista, S., Zivadinov, R., Hoogs, M., Bergsland, N., Heininen-Brown, M., Dwyer, M. G., Weinstock-Guttman, B., & Benedict, R. H. (2012). Basal ganglia, thalamus and neocortical atrophy predicting slowed cognitive processing in multiple sclerosis. *Journal of neurology*, *259*(1), 139–146. <https://doi.org/10.1007/s00415-011-6147-1>
11. Bellew, D., Davenport, L., Monaghan, R., Cogley, C., Gaughan, M., Yap, S. M., Tubridy, N., Bramham, J., McGuigan, C., & O'Keeffe, F. (2022). Interpreting the clinical importance of the relationship between subjective fatigue and cognitive impairment in multiple sclerosis (MS): How BICAMS performance is affected by MS-related fatigue. *Multiple sclerosis and related disorders*, *67*, 104161. <https://doi.org/10.1016/j.msard.2022.104161>
12. Benedict, R. H., Morrow, S. A., Weinstock Guttman, B., Cookfair, D., & Schretlen, D. J. (2010). Cognitive reserve moderates decline in information processing speed in multiple sclerosis patients. *Journal of the International Neuropsychological Society : JINS*, *16*(5), 829–835. <https://doi.org/10.1017/S1355617710000688>
13. Bergsland, N., Benedict, R. H. B., Dwyer, M. G., Fuchs, T. A., Jakimovski, D., Schweser, F., Tavazzi, E., Weinstock-Guttman, B., & Zivadinov, R. (2021). Thalamic Nuclei Volumes and Their Relationships to Neuroperformance in Multiple Sclerosis: A Cross-Sectional Structural MRI Study. *Journal of magnetic resonance imaging : JMRI*, *53*(3), 731–739. <https://doi.org/10.1002/jmri.27389>
14. Berrigan, L. I., Fisk, J. D., Walker, L. A., Wojtowicz, M., Rees, L. M., Freedman, M. S., & Marrie, R. A. (2014). Reliability of regression-based normative data for the oral symbol digit modalities test: an evaluation of demographic influences, construct validity, and impairment classification rates in multiple sclerosis samples. *The Clinical neuropsychologist*, *28*(2), 281–299. <https://doi.org/10.1080/13854046.2013.871337>
15. Betscher, E., Guenter, W., Langdon, D. W., & Bonek, R. (2021). Polish validation of the Brief International Cognitive Assessment for Multiple Sclerosis (BICAMS battery): correlation of cognitive impairment with mood disorders and fatigue. *Neurologia i neurochirurgia polska*, *55*(1), 59–66. <https://doi.org/10.5603/PJNNS.a2020.0080>
16. Birkeldh, U., Manouchehrinia, A., Hietala, M. A., Hillert, J., Olsson, T., Piehl, F., Kockum, I. S., Brundin, L., Zahavi, O., Wahlberg-Ramsay, M., Brautaset, R., & Nilsson, M. (2017). The Temporal Retinal Nerve Fiber Layer Thickness Is the Most Important Optical Coherence Tomography Estimate in Multiple Sclerosis. *Frontiers in neurology*, *8*, 675. <https://doi.org/10.3389/fneur.2017.00675>
17. Borrelli, S., Pereira Lima, J., & Dachy, B. (2023). The relation between the symbol digit modalities test, fatigue, depression, and anxiety symptoms in a Belgian MS cohort. *Acta neurologica Belgica*, *123*(6), 2147–2153. <https://doi.org/10.1007/s13760-022-02127-4>
18. Bsteh, G., Hegen, H., Altmann, P., Auer, M., Berek, K., Pauli, F. D., Wurth, S., Zinganell, A., Rommer, P., Deisenhammer, F., Leutmezer, F., & Berger, T. (2020). Retinal layer thinning is reflecting disability progression independent of relapse activity in multiple sclerosis. *Multiple sclerosis journal - experimental, translational and clinical*, *6*(4), 2055217320966344. <https://doi.org/10.1177/2055217320966344>
19. Carandini, T., Mancini, M., Bogdan, I., Rae, C. L., Barritt, A. W., Clerico, M., Sethi, A., Harrison, N., Rashid, W., Scarpini, E., Galimberti, D., Bozzali, M., & Cercignani, M. (2021). In vivo evidence of functional disconnection between brainstem monoaminergic nuclei and brain networks in multiple sclerosis. *Multiple sclerosis and related disorders*, *56*, 103224. <https://doi.org/10.1016/j.msard.2021.103224>
20. Carotenuto, A., Wilson, H., Giordano, B., Caminiti, S. P., Chappell, Z., Williams, S. C. R., Hammers, A., Silber, E., Brex, P., & Politis, M. (2020). Impaired connectivity within neuromodulatory networks in multiple sclerosis and clinical implications. *Journal of neurology*, *267*(7), 2042–2053. <https://doi.org/10.1007/s00415-020-09806-3>
21. Carotenuto, A., Costabile, T., Moccia, M., Falco, F., Petracca, M., Satelliti, B., Russo, C. V., Saccà, F., Lanzillo, R., & Brescia Morra, V. (2021). Interplay Between Cognitive and Bowel/Bladder Function in Multiple Sclerosis. *International neurourology journal*, *25*(4), 310–318. <https://doi.org/10.5213/inj.2040346.173>
22. Castillo-Triviño, T., Gómez-Ballesteros, R., Borges, M., Martín-Martínez, J., Sotoca, J., Alonso, A., Caminero, A. B., Borrega, L., Sánchez-Menoyo, J. L., Barrero-Hernández, F. J., Calles, C., Brieva, L., Blasco-Quílez, M. R., García-Soto, J. D., Del Campo-Amigo, M., Navarro-Cantó, L., Agüera, E., Garcés-Redondo, M., Carmona, O., Gabaldón-Torres, L., … Sainz de la Maza, S. (2022). Long-term prognosis communication preferences in early-stage relapsing-remitting multiple sclerosis. *Multiple sclerosis and related disorders*, *64*, 103969. <https://doi.org/10.1016/j.msard.2022.103969>
23. Cattaneo, D., Lamers, I., Bertoni, R., Feys, P., & Jonsdottir, J. (2017). Participation Restriction in People With Multiple Sclerosis: Prevalence and Correlations With Cognitive, Walking, Balance, and Upper Limb Impairments. *Archives of physical medicine and rehabilitation*, *98*(7), 1308–1315. <https://doi.org/10.1016/j.apmr.2017.02.015>
24. Chalah, M. A., Palm, U., Lefaucheur, J. P., Créange, A., & Ayache, S. S. (2018). Interhermispheric inhibition predicts anxiety levels in multiple sclerosis: A corticospinal excitability study. *Brain research*, *1699*, 186–194. <https://doi.org/10.1016/j.brainres.2018.08.029>
25. Charalambous, T., Tur, C., Prados, F., Kanber, B., Chard, D. T., Ourselin, S., Clayden, J. D., A M Gandini Wheeler-Kingshott, C., Thompson, A. J., & Toosy, A. T. (2019). Structural network disruption markers explain disability in multiple sclerosis. *Journal of neurology, neurosurgery, and psychiatry*, *90*(2), 219–226. <https://doi.org/10.1136/jnnp-2018-318440>
26. Chu, L., Casserly, C., Rosehart, H., & Morrow, S. A. (2022). Is there a multiple sclerosis personality? Personality characteristics in newly diagnosed multiple sclerosis and association with mood and cognition. *Journal of the neurological sciences*, *434*, 120145. <https://doi.org/10.1016/j.jns.2022.120145>
27. Clough, M., Foletta, P., Frohman, A. N., Sears, D., Ternes, A., White, O. B., & Fielding, J. (2018). Multiple sclerosis: Executive dysfunction, task switching and the role of attention. *Multiple sclerosis journal - experimental, translational and clinical*, *4*(2), 2055217318771781. <https://doi.org/10.1177/2055217318771781>
28. Cocozza, S., Petracca, M., Mormina, E., Buyukturkoglu, K., Podranski, K., Heinig, M. M., Pontillo, G., Russo, C., Tedeschi, E., Russo, C. V., Costabile, T., Lanzillo, R., Harel, A., Klineova, S., Miller, A., Brunetti, A., Morra, V. B., Lublin, F., & Inglese, M. (2017). Cerebellar lobule atrophy and disability in progressive MS. *Journal of neurology, neurosurgery, and psychiatry*, *88*(12), 1065–1072. <https://doi.org/10.1136/jnnp-2017-316448>
29. Cohen, J. N., Seng, E., & Foley, F. W. (2021). Cognitive and motor slowing mediate the relationship between depression and falls in multiple sclerosis patients. *Multiple sclerosis and related disorders*, *50*, 102808. <https://doi.org/10.1016/j.msard.2021.102808>
30. Costabile, T., Signoriello, E., Lauro, F., Altieri, M., Ziello, A. R., D'Ambrosio, A., Bisecco, A., Maniscalco, G., Bonavita, S., Gallo, A., Brescia Morra, V., Lus, G., Saccà, F., & Russo, C. V. (2023). Validation of an iPad version of the Brief International Cognitive Assessment for Multiple Sclerosis (BICAMS). *Multiple sclerosis and related disorders*, *74*, 104723. <https://doi.org/10.1016/j.msard.2023.104723>
31. Costers, L., Gielen, J., Eelen, P. L., Schependom, J. V., Laton, J., Remoortel, A. V., Vanzeir, E., Wijmeersch, B. V., Seeldrayers, P., Haelewyck, M. C., D'Haeseleer, M., D'hooghe, M. B., Langdon, D., & Nagels, G. (2017). Does including the full CVLT-II and BVMT-R improve BICAMS? Evidence from a Belgian (Dutch) validation study. *Multiple sclerosis and related disorders*, *18*, 33–40. <https://doi.org/10.1016/j.msard.2017.08.018>
32. Dackovic, J., Pekmezovic, T., Mesaros, S., Dujmovic, I., Stojsavljevic, N., Martinovic, V., & Drulovic, J. (2016). The Rao's Brief Repeatable Battery in the study of cognition in different multiple sclerosis phenotypes: application of normative data in a Serbian population. *Neurological sciences : official journal of the Italian Neurological Society and of the Italian Society of Clinical Neurophysiology*, *37*(9), 1475–1481. <https://doi.org/10.1007/s10072-016-2610-1>
33. Damasceno, A., Damasceno, B. P., & Cendes, F. (2014). The clinical impact of cerebellar grey matter pathology in multiple sclerosis. *PloS one*, *9*(5), e96193. <https://doi.org/10.1371/journal.pone.0096193>
34. de Caneda, M. A. G., Cuervo, D. L. M., Marinho, N. E., & de Vecino, M. C. A. (2018). The Reliability of the Brief Visuospatial Memory Test - Revised in Brazilian multiple sclerosis patients. *Dementia & neuropsychologia*, *12*(2), 205–211. <https://doi.org/10.1590/1980-57642018dn12-020014>
35. de la Peña, M. J., Peña, I. C., García, P. G., Gavilán, M. L., Malpica, N., Rubio, M., González, R. A., & de Vega, V. M. (2019). Early perfusion changes in multiple sclerosis patients as assessed by MRI using arterial spin labeling. *Acta radiologica open*, *8*(12), 2058460119894214. <https://doi.org/10.1177/2058460119894214>
36. de David, A. C., Sasaki, J. E., Ramari, C., Tauil, C. B., Moraes, A. G., Martins, F., von Glehn, F., & Motl, R. W. (2019). Validation of the Brazilian version of the patient-determined disease steps scale in persons with multiple sclerosis. *Multiple sclerosis and related disorders*, *30*, 208–214. <https://doi.org/10.1016/j.msard.2019.02.022>
37. Delgado-Álvarez, A., Matías-Guiu, J. A., Delgado-Alonso, C., Cuevas, C., Palacios-Sarmiento, M., Vidorreta-Ballesteros, L., Montero-Escribano, P., & Matías-Guiu, J. (2022). Validation of two new scales for the assessment of fatigue in Multiple Sclerosis: F-2-MS and FACIT-F. *Multiple sclerosis and related disorders*, *63*, 103826. <https://doi.org/10.1016/j.msard.2022.103826>
38. DiGiuseppe, G., Blair, M., & Morrow, S. A. (2018). *Short Report:* Prevalence of Cognitive Impairment in Newly Diagnosed Relapsing-Remitting Multiple Sclerosis. *International journal of MS care*, *20*(4), 153–157. <https://doi.org/10.7224/1537-2073.2017-029>
39. Dinoto, A., Baldini, S., Morelli, M. E., Pasquin, F., Bratina, A., Bosco, A., Sartori, A., & Manganotti, P. (2021). Unveiling the relationship between autonomic involvement, fatigue, and cognitive dysfunction in early relapsing-remitting multiple sclerosis. *Neurological sciences : official journal of the Italian Neurological Society and of the Italian Society of Clinical Neurophysiology*, *42*(10), 4281–4287. <https://doi.org/10.1007/s10072-021-05487-6>
40. Duque, B., Sepulcre, J., Bejarano, B., Samaranch, L., Pastor, P., & Villoslada, P. (2008). Memory decline evolves independently of disease activity in MS. *Multiple sclerosis (Houndmills, Basingstoke, England)*, *14*(7), 947–953. <https://doi.org/10.1177/1352458508089686>
41. Dusankova, J. B., Kalincik, T., Havrdova, E., & Benedict, R. H. (2012). Cross cultural validation of the Minimal Assessment of Cognitive Function in Multiple Sclerosis (MACFIMS) and the Brief International Cognitive Assessment for Multiple Sclerosis (BICAMS). *The Clinical neuropsychologist*, *26*(7), 1186–1200. <https://doi.org/10.1080/13854046.2012.725101>
42. Eizaguirre, M. B., Vanotti, S., Merino, Á., Yastremiz, C., Silva, B., Alonso, R., & Garcea, O. (2018). The Role of Information Processing Speed in Clinical and Social Support Variables of Patients with Multiple Sclerosis. *Journal of clinical neurology (Seoul, Korea)*, *14*(4), 472–477. <https://doi.org/10.3988/jcn.2018.14.4.472>
43. El Ghoneimy, A.T., Hassan, A., Homos, M., Farghaly, M., & Dahshan, A. (2015). Thalamic involvement and its impact on disability and cognition in multiple sclerosis: A clinical and diffusion tensor imaging study. *Egyptian Journal of Neurology Psychiatry and Neurosurgery, 52*(2), 139–145.
44. Eshaghi, A., Riyahi-Alam, S., Roostaei, T., Haeri, G., Aghsaei, A., Aidi, M. R., Pouretemad, H. R., Zarei, M., Farhang, S., Saeedi, R., Nazeri, A., Ganjgahi, H., Etesam, F., Azimi, A. R., Benedict, R. H., & Sahraian, M. A. (2012). Validity and reliability of a Persian translation of the Minimal Assessment of Cognitive Function in Multiple Sclerosis (MACFIMS). *The Clinical neuropsychologist*, *26*(6), 975–984. <https://doi.org/10.1080/13854046.2012.694912>
45. Eskut, N., Koc, A. M., Koskderelioglu, A., Dilek, I., & Tekindal, M. A. (2023). Correlation of brain segmental volume changes with clinical parameters: a longitudinal study in multiple sclerosis patients. Correlação das alterações do volume segmentar cerebral com parâmetros clínicos: um estudo longitudinal em pacientes com esclerose múltipla. *Arquivos de neuro-psiquiatria*, *81*(2), 164–172. <https://doi.org/10.1055/s-0043-1761492>
46. Estiasari, R., Fajrina, Y., Lastri, D. N., Melani, S., Maharani, K., Imran, D., Pangeran, D., & Sitorus, F. (2019). Validity and Reliability of Brief International Cognitive Assessment for Multiple Sclerosis (BICAMS) in Indonesia and the Correlation with Quality of Life. *Neurology research international*, *2019*, 4290352. <https://doi.org/10.1155/2019/4290352>
47. Evdoshenko, E., Laskova, K., Shumilina, M., Nekrashevich, E., Andreeva, M., Neofidov, N., Kalinin, I., Nikitchenko, D., Rogozina, A., Kupaeva, A., Bulanov, I., Bakirtzis, C., Grigoriadis, N., Khachanova, N., Davydovskaya, M., & Makshakov, G. (2022). Validation of the Brief International Cognitive Assessment for Multiple Sclerosis (BICAMS) in the Russian Population. *Journal of the International Neuropsychological Society : JINS*, *28*(5), 503–510. <https://doi.org/10.1017/S1355617721000722>
48. Faragó, P., Tóth, E., Szabó N, N., Kocsis, K., Kincses, B., Bozsik, B., Veréb, D., Biernacki, T. I., Király, A., Sandi, D., Bencsik, K., Klivényi, P., Vécsei, L., & Kincses, Z. T. (2022). Connection between microstructural alterations detected by diffusion MRI and cognitive dysfunction in MS: A model-free analysis approach. *Multiple sclerosis and related disorders*, *57*, 103442. <https://doi.org/10.1016/j.msard.2021.103442>
49. Farghaly M, Langdon DW, Shalaby NM, Shehata HS, Abokrysha NT, Hassan A, et al. Reliability and validity of Arabic version of the brief international cognitive assessment for multiple sclerosis: Egyptian dialect. Egypt J Neurol Psychiatry Neurosurg. 2021;57:51. https://doi.org/10.1186/ s41983-021-00303-6
50. Figved, N., Benedict, R., Klevan, G., Myhr, K. M., Nyland, H. I., Landrø, N. I., Larsen, J. P., & Aarsland, D. (2008). Relationship of cognitive impairment to psychiatric symptoms in multiple sclerosis. *Multiple sclerosis (Houndmills, Basingstoke, England)*, *14*(8), 1084–1090. <https://doi.org/10.1177/1352458508092262>
51. Fritz, N. E., Roy, S., Keller, J., Prince, J., Calabresi, P. A., & Zackowski, K. M. (2016). Pain, cognition and quality of life associate with structural measures of brain volume loss in multiple sclerosis. *NeuroRehabilitation*, *39*(4), 535–544. <https://doi.org/10.3233/NRE-161384>
52. Fuchs, T. A., Gillies, J., Jaworski, M. G., 3rd, Wilding, G. E., Youngs, M., Weinstock-Guttman, B., & Benedict, R. H. (2022). Repeated forms, testing intervals, and SDMT performance in a large multiple sclerosis dataset. *Multiple sclerosis and related disorders*, *68*, 104375. <https://doi.org/10.1016/j.msard.2022.104375>
53. Gao, K. C., Nair, G., Cortese, I. C., Koretsky, A., & Reich, D. S. (2014). Sub-millimeter imaging of brain-free water for rapid volume assessment in atrophic brains. *NeuroImage*, *100*, 370–378. <https://doi.org/10.1016/j.neuroimage.2014.06.014>
54. Gaughan, M., Monaghan, R., O'Connell, K., McNicholas, N., Yap, S. M., Tubridy, N., O'Keeffe, F., & McGuigan, C. (2021). Five-year follow up of the original Irish BICAMS validation cohort. *Multiple sclerosis and related disorders*, *56*, 103257. <https://doi.org/10.1016/j.msard.2021.103257>
55. Giedraitienė, N., Kizlaitienė, R., & Kaubrys, G. (2015). The BICAMS Battery for Assessment of Lithuanian-Speaking Multiple Sclerosis Patients: Relationship with Age, Education, Disease Disability, and Duration. *Medical science monitor : international medical journal of experimental and clinical research*, *21*, 3853–3859. <https://doi.org/10.12659/msm.896571>
56. Gill, S., Santo, J., Blair, M., & Morrow, S. A. (2019). Depressive Symptoms Are Associated With More Negative Functional Outcomes Than Anxiety Symptoms in Persons With Multiple Sclerosis. *The Journal of neuropsychiatry and clinical neurosciences*, *31*(1), 37–42. <https://doi.org/10.1176/appi.neuropsych.18010011>
57. Glanz, B. I., Healy, B. C., Hviid, L. E., Chitnis, T., & Weiner, H. L. (2012). Cognitive deterioration in patients with early multiple sclerosis: a 5-year study. *Journal of neurology, neurosurgery, and psychiatry*, *83*(1), 38–43. <https://doi.org/10.1136/jnnp.2010.237834>
58. Gouveia, A., Dias, S. P., Santos, T., Rocha, H., Coelho, C. R., Ruano, L., Galego, O., Diogo, M. C., Seixas, D., Sá, M. J., & Batista, S. (2017). Cognitive impairment and magnetic resonance imaging correlates in primary progressive multiple sclerosis. *Acta neurologica Scandinavica*, *136*(2), 109–115. <https://doi.org/10.1111/ane.12702>
59. Grothe, L., Grothe, M., Wingert, J., Schomerus, G., & Speerforck, S. (2022). Stigma in Multiple Sclerosis: The Important Role of Sense of Coherence and Its Relation to Quality of Life. *International journal of behavioral medicine*, *29*(4), 517–523. <https://doi.org/10.1007/s12529-021-10030-0>
60. Guenter, W., Betscher, E., & Bonek, R. (2022). Predictive Value of the Third Ventricle Width for Neurological Status in Multiple Sclerosis. *Journal of clinical medicine*, *11*(10), 2841. <https://doi.org/10.3390/jcm11102841>
61. Hämäläinen, P., Leo, V., Therman, S., & Ruutiainen, J. (2021). Validation of the Finnish version of the Brief International Cognitive Assessment for Multiple Sclerosis (BICAMS) and evaluation of the applicability of the Multiple Sclerosis Neuropsychological Questionnaire (MSNQ) and the Fatigue Scale for Motor and Cognitive Functions (FSMC). *Brain and behavior*, *11*(6), e02087. <https://doi.org/10.1002/brb3.2087>
62. Has Silemek, A. C., Fischer, L., Pöttgen, J., Penner, I. K., Engel, A. K., Heesen, C., Gold, S. M., & Stellmann, J. P. (2020). Functional and structural connectivity substrates of cognitive performance in relapsing remitting multiple sclerosis with mild disability. *NeuroImage. Clinical*, *25*, 102177. <https://doi.org/10.1016/j.nicl.2020.102177>
63. Higueras, Y., Salas, E., Meca-Lallana, V., Carrascal Rueda, P., Rodríguez De la Fuente, O., Cabello-Moruno, R., Maurino, J., & Ruiz Díaz, M. Á. (2022). Information-Seeking Strategies of People with Multiple Sclerosis in Spain: The INFOSEEK-MS Study. *Patient preference and adherence*, *16*, 51–60. <https://doi.org/10.2147/PPA.S344690>
64. Hildesheim, F. E., Benedict, R. H. B., Zivadinov, R., Dwyer, M. G., Fuchs, T., Jakimovski, D., Weinstock-Guttman, B., & Bergsland, N. (2021). Nucleus basalis of Meynert damage and cognition in patients with multiple sclerosis. *Journal of neurology*, *268*(12), 4796–4808. <https://doi.org/10.1007/s00415-021-10594-7>
65. Hoogs, M., Kaur, S., Smerbeck, A., Weinstock-Guttman, B., & Benedict, R. H. (2011). Cognition and physical disability in predicting health-related quality of life in multiple sclerosis. *International journal of MS care*, *13*(2), 57–63. <https://doi.org/10.7224/1537-2073-13.2.57>
66. Jacobsen, C., Zivadinov, R., Myhr, K. M., Dalaker, T. O., Dalen, I., Benedict, R. H., Bergsland, N., & Farbu, E. (2021). Brain atrophy and clinical characteristics predicting SDMT performance in multiple sclerosis: A 10-year follow-up study. *Multiple sclerosis journal - experimental, translational and clinical*, *7*(1), 2055217321992394. <https://doi.org/10.1177/2055217321992394>
67. Jakimovski, D., Benedict, R. H., Marr, K., Gandhi, S., Bergsland, N., Weinstock-Guttman, B., & Zivadinov, R. (2020). Lower total cerebral arterial flow contributes to cognitive performance in multiple sclerosis patients. *Multiple sclerosis (Houndmills, Basingstoke, England)*, *26*(2), 201–209. <https://doi.org/10.1177/1352458518819608>
68. Jamoussi, H., Ali, N. B., Missaoui, Y., Cherif, A., Oudia, N., Anane, N., Ftouhi, L., Mahmoud, M. B., Fray, S., & Fredj, M. (2023). Cognitive impairment in multiple sclerosis: Utility of electroencephalography. *Multiple sclerosis and related disorders*, *70*, 104502. <https://doi.org/10.1016/j.msard.2023.104502>
69. Kantorová, E., Hnilicová, P., Bogner, W., Grendár, M., Grossmann, J., Kováčová, S., Hečková, E., Strasser, B., Čierny, D., Zeleňák, K., & Kurča, E. (2022). Neurocognitive performance in relapsing-remitting multiple sclerosis patients is associated with metabolic abnormalities of the thalamus but not the hippocampus- GABA-edited 1H MRS study. *Neurological research*, *44*(1), 57–64. <https://doi.org/10.1080/01616412.2021.1956282>
70. Karpuz Seren, B., Acikgoz, M., Piri Cinar, B., Aciman Demirel, E., Celebi, U., & Atasoy, H. T. (2022). The relationship between alexithymia, reading the mind in the eyes and cognition in patients with multiple sclerosis. *Multiple sclerosis and related disorders*, *68*, 104196. <https://doi.org/10.1016/j.msard.2022.104196>
71. Kever, A., Buyukturkoglu, K., Riley, C. S., De Jager, P. L., & Leavitt, V. M. (2021). Social support is linked to mental health, quality of life, and motor function in multiple sclerosis. *Journal of neurology*, *268*(5), 1827–1836. <https://doi.org/10.1007/s00415-020-10330-7>
72. Khaligh-Razavi, S. M., Sadeghi, M., Khanbagi, M., Kalafatis, C., & Nabavi, S. M. (2020). A self-administered, artificial intelligence (AI) platform for cognitive assessment in multiple sclerosis (MS). *BMC neurology*, *20*(1), 193. <https://doi.org/10.1186/s12883-020-01736-x>
73. Khalil, H., Al-Shorman, A., El-Salem, K., Abdo, N., Alghwiri, A. A., Aburub, A., Shalabi, S., & Al-Mustafa, F. (2017). Fear of Falling in People With Multiple Sclerosis: Which Clinical Characteristics Are Important?. *Physical therapy*, *97*(7), 698–706. <https://doi.org/10.1093/ptj/pzx044>
74. Khan, A., Li, Y., Ponirakis, G., Akhtar, N., Gad, H., George, P., Ibrahim, F. M., Petropoulos, I. N., Canibano, B. G., Deleu, D., Shuaib, A., Kamran, S., & Malik, R. A. (2021). Corneal Immune Cells Are Increased in Patients With Multiple Sclerosis. *Translational vision science & technology*, *10*(4), 19. <https://doi.org/10.1167/tvst.10.4.19>
75. Khedr EM, Desoky T, Gamea A, Ezzeldin MY, Zaki AF. Thalamic atrophy, duration of illness, and years of education are the best predictors of cognitive impairment in multiple sclerosis. *Clin Exp Neuroimmunol*. 2022; 13(4): 216–225. <https://doi.org/10.1111/cen3.12705>
76. Labiano-Fontcuberta, A., Mitchell, A. J., Moreno-García, S., & Benito-León, J. (2014). Cognitive impairment in patients with multiple sclerosis predicts worse caregiver's health-related quality of life. *Multiple sclerosis (Houndmills, Basingstoke, England)*, *20*(13), 1769–1779. <https://doi.org/10.1177/1352458514532398>
77. Lam, K. H., Meijer, K. A., Loonstra, F. C., Coerver, E., Twose, J., Redeman, E., Moraal, B., Barkhof, F., de Groot, V., Uitdehaag, B., & Killestein, J. (2021). Real-world keystroke dynamics are a potentially valid biomarker for clinical disability in multiple sclerosis. *Multiple sclerosis (Houndmills, Basingstoke, England)*, *27*(9), 1421–1431. <https://doi.org/10.1177/1352458520968797>
78. Lebkuecher, A. L., Chiaravalloti, N. D., & Strober, L. B. (2021). The role of language ability in verbal fluency of individuals with multiple sclerosis. *Multiple sclerosis and related disorders*, *50*, 102846. <https://doi.org/10.1016/j.msard.2021.102846>
79. Loitfelder, M., Fazekas, F., Koschutnig, K., Fuchs, S., Petrovic, K., Ropele, S., Pichler, A., Jehna, M., Langkammer, C., Schmidt, R., Neuper, C., & Enzinger, C. (2014). Brain activity changes in cognitive networks in relapsing-remitting multiple sclerosis - insights from a longitudinal FMRI study. *PloS one*, *9*(4), e93715. <https://doi.org/10.1371/journal.pone.0093715>
80. López-Góngora, M., Querol, L., & Escartín, A. (2015). A one-year follow-up study of the Symbol Digit Modalities Test (SDMT) and the Paced Auditory Serial Addition Test (PASAT) in relapsing-remitting multiple sclerosis: an appraisal of comparative longitudinal sensitivity. *BMC neurology*, *15*, 40. <https://doi.org/10.1186/s12883-015-0296-2>
81. Lopez-Soley, E., Martinez-Heras, E., Solana, E., Solanes, A., Radua, J., Vivo, F., Prados, F., Sepulveda, M., Cabrera-Maqueda, J. M., Fonseca, E., Blanco, Y., Alba-Arbalat, S., Martinez-Lapiscina, E. H., Villoslada, P., Saiz, A., & Llufriu, S. (2023). Diffusion tensor imaging metrics associated with future disability in multiple sclerosis. *Scientific reports*, *13*(1), 3565. <https://doi.org/10.1038/s41598-023-30502-5>
82. Louapre, C., Govindarajan, S. T., Giannì, C., Cohen-Adad, J., Gregory, M. D., Nielsen, A. S., Madigan, N., Sloane, J. A., Kinkel, R. P., & Mainero, C. (2016). Is the Relationship between Cortical and White Matter Pathologic Changes in Multiple Sclerosis Spatially Specific? A Multimodal 7-T and 3-T MR Imaging Study with Surface and Tract-based Analysis. *Radiology*, *278*(2), 524–535. <https://doi.org/10.1148/radiol.2015150486>
83. Mackay, L., Johnson, A. M., Moodie, S. T., Rosehart, H., & Morrow, S. A. (2021). Predictors of cognitive fatigue and fatigability in multiple sclerosis. *Multiple sclerosis and related disorders*, *56*, 103316. <https://doi.org/10.1016/j.msard.2021.103316>
84. Maltby, V. E., Lea, R. A., Reeves, P., Saugbjerg, B., & Lechner-Scott, J. (2022). Reduced cognitive function contributes to economic burden of multiple sclerosis. *Multiple sclerosis and related disorders*, *60*, 103707. <https://doi.org/10.1016/j.msard.2022.103707>
85. Marinetto, S., Riccardi, A., Barbadoro, F., Pucci, V., Selini, E., Pavan, A., Rinaldi, F., Perini, P., Puthenparampil, M., Gallo, P., & Mondini, S. (2023). Sexual Dysfunction in Multiple Sclerosis: The Role of Executive Function. *Behavioral sciences (Basel, Switzerland)*, *13*(5), 369. <https://doi.org/10.3390/bs13050369>
86. Marstrand, L., Østerberg, O., Walsted, T., Skov, A. C., Schreiber, K. I., & Sellebjerg, F. (2020). Brief international cognitive assessment for multiple sclerosis (BICAMS): A danish validation study of sensitivity in early stages of MS. *Multiple sclerosis and related disorders*, *37*, 101458. <https://doi.org/10.1016/j.msard.2019.101458>
87. Martí-Juan, G., Sastre-Garriga, J., Martinez-Heras, E., Vidal-Jordana, A., Llufriu, S., Groppa, S., Gonzalez-Escamilla, G., Rocca, M. A., Filippi, M., Høgestøl, E. A., Harbo, H. F., Foster, M. A., Toosy, A. T., Schoonheim, M. M., Tewarie, P., Pontillo, G., Petracca, M., Rovira, À., Deco, G., & Pareto, D. (2023). Using The Virtual Brain to study the relationship between structural and functional connectivity in patients with multiple sclerosis: a multicenter study. *Cerebral cortex (New York, N.Y. : 1991)*, *33*(12), 7322–7334. <https://doi.org/10.1093/cercor/bhad041>
88. Matías-Guiu, J. A., Sánchez-Benavides, G., Rivera-Àvila, N., Cortés-Martínez, A., Delgado-Alonso, C., Delgado-Álvarez, A., Montero, P., Pytel, V., Matías-Guiu, J., & Peña-Casanova, J. (2020). Validation of the Neuronorma battery for neuropsychological assessment in multiple sclerosis. *Multiple sclerosis and related disorders*, *42*, 102070. <https://doi.org/10.1016/j.msard.2020.102070>
89. Maubeuge, N., Deloire, M. S. A., Brochet, B., Erhlé, N., Charré-Morin, J., Saubusse, A., Ruet, A., & BICAFMS study investigators (2021). French validation of the Brief International Cognitive Assessment for Multiple Sclerosis. *Revue neurologique*, *177*(1-2), 73–79. <https://doi.org/10.1016/j.neurol.2020.04.028>
90. McKay, K. A., Manouchehrinia, A., Berrigan, L., Fisk, J. D., Olsson, T., & Hillert, J. (2019). Long-term Cognitive Outcomes in Patients With Pediatric-Onset vs Adult-Onset Multiple Sclerosis. *JAMA neurology*, *76*(9), 1028–1034. <https://doi.org/10.1001/jamaneurol.2019.1546>
91. Motl, R. W., Cadavid, D., Sandroff, B. M., Pilutti, L. A., Pula, J. H., & Benedict, R. H. (2013). Cognitive processing speed has minimal influence on the construct validity of Multiple Sclerosis Walking Scale-12 scores. *Journal of the neurological sciences*, *335*(1-2), 169–173. <https://doi.org/10.1016/j.jns.2013.09.024>
92. Neuhaus, M., Bagutti, S., Yaldizli, Ö., Zwahlen, D., Schaub, S., Frey, B., Fischer-Barnicol, B., Burgunder, J. M., Martory, M. D., Pöttgen, J., Annoni, J. M., & Penner, I. K. (2018). Characterization of social cognition impairment in multiple sclerosis. *European journal of neurology*, *25*(1), 90–96. <https://doi.org/10.1111/ene.13457>
93. Niino, M., Mifune, N., Kohriyama, T., Mori, M., Ohashi, T., Kawachi, I., Shimizu, Y., Fukaura, H., Nakashima, I., Kusunoki, S., Miyamoto, K., Yoshida, K., Kanda, T., Nomura, K., Yamamura, T., Yoshii, F., Kira, J., Nakane, S., Yokoyama, K., Matsui, M., … Kikuchi, S. (2014). Apathy/depression, but not subjective fatigue, is related with cognitive dysfunction in patients with multiple sclerosis. *BMC neurology*, *14*, 3. <https://doi.org/10.1186/1471-2377-14-3>
94. Nocentini, U., Pasqualetti, P., Bonavita, S., Buccafusca, M., De Caro, M. F., Farina, D., Girlanda, P., Le Pira, F., Lugaresi, A., Quattrone, A., Reggio, A., Salemi, G., Savettieri, G., Tedeschi, G., Trojano, M., Valentino, P., & Caltagirone, C. (2006). Cognitive dysfunction in patients with relapsing-remitting multiple sclerosis. *Multiple sclerosis (Houndmills, Basingstoke, England)*, *12*(1), 77–87. <https://doi.org/10.1191/135248506ms1227oa>
95. Noori, H., Gheini, M. R., Rezaeimanesh, N., Saeedi, R., Rezaei Aliabadi, H., Sahraian, M. A., & Naser Moghadasi, A. (2019). The correlation between dyslipidemia and cognitive impairment in multiple sclerosis patients. *Multiple sclerosis and related disorders*, *36*, 101415. <https://doi.org/10.1016/j.msard.2019.101415>
96. Ntoskou, K., Messinis, L., Nasios, G., Martzoukou, M., Makris, G., Panagiotopoulos, E., & Papathanasopoulos, P. (2018). Cognitive and Language Deficits in Multiple Sclerosis: Comparison of Relapsing Remitting and Secondary Progressive Subtypes. *The open neurology journal*, *12*, 19–30. <https://doi.org/10.2174/1874205X01812010019>
97. O'Connell, K., Langdon, D., Tubridy, N., Hutchinson, M., & McGuigan, C. (2015). A preliminary validation of the brief international cognitive assessment for multiple sclerosis (BICAMS) tool in an Irish population with multiple sclerosis (MS). *Multiple sclerosis and related disorders*, *4*(6), 521–525. <https://doi.org/10.1016/j.msard.2015.07.012>
98. de Oliveira, M., Santinelli, F. B., Lisboa-Filho, P. N., & Barbieri, F. A. (2023). The Blood Concentration of Metallic Nanoparticles Is Related to Cognitive Performance in People with Multiple Sclerosis: An Exploratory Analysis. *Biomedicines*, *11*(7), 1819. <https://doi.org/10.3390/biomedicines11071819>
99. Ozturk, O., Ozturk, S., Gul, S., Bunul, S. D., Efendi, H., & Cam, I. (2021). The relationship between cerebellar volume, clinical disability and cognitive changes in multiple sclerosis patients. *Neurology Asia*, *26*(2), 341–346.
100. Paolicelli, D., Manni, A., Iaffaldano, A., Tancredi, G., Ricci, K., Gentile, E., Viterbo, R. G., Quitadamo, S. G., de Tommaso, M., & Trojano, M. (2021). Magnetoencephalography and High-Density Electroencephalography Study of Acoustic Event Related Potentials in Early Stage of Multiple Sclerosis: A Pilot Study on Cognitive Impairment and Fatigue. *Brain sciences*, *11*(4), 481. <https://doi.org/10.3390/brainsci11040481>
101. Parmar, K., Fonov, V. S., Naegelin, Y., Amann, M., Wuerfel, J., Collins, D. L., Gaetano, L., Magon, S., Sprenger, T., Kappos, L., Granziera, C., & Tsagkas, C. (2022). Regional Cerebellar Volume Loss Predicts Future Disability in Multiple Sclerosis Patients. *Cerebellum (London, England)*, *21*(4), 632–646. <https://doi.org/10.1007/s12311-021-01312-0>
102. Parmenter, B. A., Weinstock-Guttman, B., Garg, N., Munschauer, F., & Benedict, R. H. (2007). Screening for cognitive impairment in multiple sclerosis using the Symbol digit Modalities Test. *Multiple sclerosis (Houndmills, Basingstoke, England)*, *13*(1), 52–57. <https://doi.org/10.1177/1352458506070750>
103. Patel, V. P., Shen, L., Rose, J., & Feinstein, A. (2019). Taking the tester out of the SDMT: A proof of concept fully automated approach to assessing processing speed in people with MS. *Multiple sclerosis (Houndmills, Basingstoke, England)*, *25*(11), 1506–1513. <https://doi.org/10.1177/1352458518792772>
104. Pavisian, B., Patel, V. P., & Feinstein, A. (2019). Cognitive mediated eye movements during the SDMT reveal the challenges with processing speed faced by people with MS. *BMC neurology*, *19*(1), 340. <https://doi.org/10.1186/s12883-019-1543-8>
105. Pérez-Miralles, F. C., Prefasi, D., García-Merino, A., Ara, J. R., Izquierdo, G., Meca-Lallana, V., Gascón-Giménez, F., Martínez-Ginés, M. L., Ramió-Torrentà, L., Costa-Frossard, L., Fernández, Ó., Moreno-García, S., Maurino, J., Carreres-Polo, J., & Casanova, B. (2021). Brain region volumes and their relationship with disability progression and cognitive function in primary progressive multiple sclerosis. *Brain and behavior*, *11*(4), e02044. <https://doi.org/10.1002/brb3.2044>
106. Pinter, D., Khalil, M., Pirpamer, L., Damulina, A., Pichler, A., Fruhwirth, V., Ropele, S., Schmidt, R., Fuchs, S., & Enzinger, C. (2021). Long-term course and morphological MRI correlates of cognitive function in multiple sclerosis. *Multiple sclerosis (Houndmills, Basingstoke, England)*, *27*(6), 954–963. <https://doi.org/10.1177/1352458520941474>
107. Podda, J., Ponzio, M., Pedullà, L., Monti Bragadin, M., Battaglia, M. A., Zaratin, P., Brichetto, G., & Tacchino, A. (2021). Predominant cognitive phenotypes in multiple sclerosis: Insights from patient-centered outcomes. *Multiple sclerosis and related disorders*, *51*, 102919. <https://doi.org/10.1016/j.msard.2021.102919>
108. Pokryszko-Dragan, A., Banaszek, A., Nowakowska-Kotas, M., Jeżowska-Jurczyk, K., Dziadkowiak, E., Gruszka, E., Zagrajek, M., Bilińska, M., Budrewicz, S., Sąsiadek, M., & Bladowska, J. (2018). Diffusion tensor imaging findings in the multiple sclerosis patients and their relationships to various aspects of disability. *Journal of the neurological sciences*, *391*, 127–133. <https://doi.org/10.1016/j.jns.2018.06.007>
109. Povolo, C. A., Blair, M., Mehta, S., Rosehart, H., & Morrow, S. A. (2019). Predictors of vocational status among persons with multiple sclerosis. *Multiple sclerosis and related disorders*, *36*, 101411. <https://doi.org/10.1016/j.msard.2019.101411>
110. Printza, A., Boziki, M., Valsamidis, C., Bakirtzis, C., Constantinidis, J., Grigoriadis, N., & Triaridis, S. (2022). Smell as a Disease Marker in Multiple Sclerosis. *Journal of clinical medicine*, *11*(17), 5215. <https://doi.org/10.3390/jcm11175215>
111. Quinn, G., Comber, L., McGuigan, C., Hannigan, A., Galvin, R., & Coote, S. (2021). Risk factors for falling for people with Multiple Sclerosis identified in a prospective cohort study. *Clinical rehabilitation*, *35*(5), 765–774. <https://doi.org/10.1177/0269215520973197>
112. Radetz, A., Mladenova, K., Ciolac, D., Gonzalez-Escamilla, G., Fleischer, V., Ellwardt, E., Krämer, J., Bittner, S., Meuth, S. G., Muthuraman, M., & Groppa, S. (2021). Linking Microstructural Integrity and Motor Cortex Excitability in Multiple Sclerosis. *Frontiers in immunology*, *12*, 748357. <https://doi.org/10.3389/fimmu.2021.748357>
113. Rasche, L., Scheel, M., Otte, K., Althoff, P., van Vuuren, A. B., Gieß, R. M., Kuchling, J., Bellmann-Strobl, J., Ruprecht, K., Paul, F., Brandt, A. U., & Schmitz-Hübsch, T. (2018). MRI Markers and Functional Performance in Patients With CIS and MS: A Cross-Sectional Study. *Frontiers in neurology*, *9*, 718. <https://doi.org/10.3389/fneur.2018.00718>
114. Reia, A., Petruzzo, M., Falco, F., Costabile, T., Conenna, M., Carotenuto, A., Petracca, M., Servillo, G., Lanzillo, R., Brescia Morra, V., & Moccia, M. (2021). A Retrospective Exploratory Analysis on Cardiovascular Risk and Cognitive Dysfunction in Multiple Sclerosis. *Brain sciences*, *11*(4), 502. <https://doi.org/10.3390/brainsci11040502>
115. Riccardi, A., Ognibene, F., Mondini, S., Nucci, M., Margoni, M., Meglioranzi, I., Carta, E., Zywicki, S., Miante, S., Perini, P., Rinaldi, F., Puthenparampil, M., & Gallo, P. (2021). Designing a Self-Perception Cognitive Questionnaire for Italian Multiple Sclerosis Patients (Sclerosi Multipla Autovalutazione Cognitiva, SMAC). A Preliminary Exploratory Pilot Study. *Frontiers in neurology*, *12*, 668933. <https://doi.org/10.3389/fneur.2021.668933>
116. Roberg, B. L., & Bruce, J. M. (2016). Reconsidering outdoor temperature and cognition in multiple sclerosis. *Multiple sclerosis (Houndmills, Basingstoke, England)*, *22*(5), 694–697. <https://doi.org/10.1177/1352458515575172>
117. Rodgers, J. D., Tjaden, K., Feenaughty, L., Weinstock-Guttman, B., & Benedict, R. H. (2013). Influence of cognitive function on speech and articulation rate in multiple sclerosis. *Journal of the International Neuropsychological Society : JINS*, *19*(2), 173–180. <https://doi.org/10.1017/S1355617712001166>
118. Rosenstein, I., Axelsson, M., Novakova, L., Rasch, S., Blennow, K., Zetterberg, H., & Lycke, J. (2023). High levels of kappa free light chain synthesis predict cognitive decline in relapsing-remitting multiple sclerosis. *Frontiers in immunology*, *14*, 1106028. <https://doi.org/10.3389/fimmu.2023.1106028>
119. Roura, E., Maclair, G., Andorrà, M., Juanals, F., Pulido-Valdeolivas, I., Saiz, A., Blanco, Y., Sepulveda, M., Llufriu, S., Martínez-Heras, E., Solana, E., Martinez-Lapiscina, E. H., & Villoslada, P. (2021). Cortical fractal dimension predicts disability worsening in Multiple Sclerosis patients. *NeuroImage. Clinical*, *30*, 102653. <https://doi.org/10.1016/j.nicl.2021.102653>
120. Roy, S., Rodgers, J., Drake, A. S., Zivadinov, R., Weinstock-Guttman, B., & Benedict, R. H. (2016). Stable neuropsychiatric status in multiple sclerosis: a 3-year study. *Multiple sclerosis (Houndmills, Basingstoke, England)*, *22*(4), 569–574. <https://doi.org/10.1177/1352458515597570>
121. Ruet, A., Deloire, M. S., Charré-Morin, J., Hamel, D., & Brochet, B. (2013). A new computerised cognitive test for the detection of information processing speed impairment in multiple sclerosis. *Multiple sclerosis (Houndmills, Basingstoke, England)*, *19*(12), 1665–1672. <https://doi.org/10.1177/1352458513480251>
122. Sandi, D., Rudisch, T., Füvesi, J., Fricska-Nagy, Z., Huszka, H., Biernacki, T., Langdon, D. W., Langane, É., Vécsei, L., & Bencsik, K. (2015). The Hungarian validation of the Brief International Cognitive Assessment for Multiple Sclerosis (BICAMS) battery and the correlation of cognitive impairment with fatigue and quality of life. *Multiple sclerosis and related disorders*, *4*(6), 499–504. <https://doi.org/10.1016/j.msard.2015.07.006>
123. Sandroff, B. M., Pilutti, L. A., & Motl, R. W. (2019). Cardiorespiratory fitness and cognitive processing speed in multiple sclerosis: The possible roles of psychological symptoms. *Multiple sclerosis and related disorders*, *27*, 23–29. <https://doi.org/10.1016/j.msard.2018.09.033>
124. Sandry, J., Simonet, D. V., Brandstadter, R., Krieger, S., Katz Sand, I., Graney, R. A., Buchanan, A. V., Lall, S., & Sumowski, J. F. (2021). The Symbol Digit Modalities Test (SDMT) is sensitive but non-specific in MS: Lexical access speed, memory, and information processing speed independently contribute to SDMT performance. *Multiple sclerosis and related disorders*, *51*, 102950. <https://doi.org/10.1016/j.msard.2021.102950>
125. Saposnik, G., Andhavarapu, S., Sainz de la Maza, S., Castillo-Triviño, T., Borges, M., Barón, B. P., Sotoca, J., Alonso, A., Caminero, A. B., Borrega, L., Sánchez-Menoyo, J. L., Barrero-Hernández, F. J., Calles, C., Brieva, L., Blasco, M. R., García-Soto, J. D., Del Campo-Amigo, M., Navarro-Cantó, L., Agüera, E., Garcés, M., … Maurino, J. (2022). Delayed cognitive processing and treatment status quo bias in early-stage multiple sclerosis. *Multiple sclerosis and related disorders*, *68*, 104138. <https://doi.org/10.1016/j.msard.2022.104138>
126. Savini, G., Pardini, M., Castellazzi, G., Lascialfari, A., Chard, D., D'Angelo, E., & Gandini Wheeler-Kingshott, C. A. M. (2019). Default Mode Network Structural Integrity and Cerebellar Connectivity Predict Information Processing Speed Deficit in Multiple Sclerosis. *Frontiers in cellular neuroscience*, *13*, 21. <https://doi.org/10.3389/fncel.2019.00021>
127. Schiavi, S., Petracca, M., Sun, P., Fleysher, L., Cocozza, S., El Mendili, M. M., Signori, A., Babb, J. S., Podranski, K., Song, S. K., & Inglese, M. (2021). Non-invasive quantification of inflammation, axonal and myelin injury in multiple sclerosis. *Brain : a journal of neurology*, *144*(1), 213–223. <https://doi.org/10.1093/brain/awaa381>
128. Schmidt, S. L., Santos da Silva, M., Schmidt, J. J., Carvalho, A. L. N., Vasconcelos, C. C. F., Paes, R. A., Boechat, Y. E., Neder, R., & Alvarenga, R. P. (2018). Neuropsychiatric assessments in patients with multiple sclerosis in early phases and with low disability. *Neuropsychiatric disease and treatment*, *14*, 1665–1670. <https://doi.org/10.2147/NDT.S163480>
129. Shi, Z., Pan, Y., Yan, Z., Ding, S., Hu, H., Wei, Y., Luo, D., Xu, Y., Zhu, Q., & Li, Y. (2023). Microstructural alterations in different types of lesions and their perilesional white matter in relapsing-remitting multiple sclerosis based on diffusion kurtosis imaging. *Multiple sclerosis and related disorders*, *71*, 104572. <https://doi.org/10.1016/j.msard.2023.104572>
130. Siddiqui, K., Browne, R. W., Benedict, R. H. B., Jakimovski, D., Weinstock-Guttman, B., Zivadinov, R., & Ramanathan, M. (2023). Cholesterol pathway biomarkers are associated with neuropsychological measures in multiple sclerosis. *Multiple sclerosis and related disorders*, *69*, 104374. <https://doi.org/10.1016/j.msard.2022.104374>
131. Siepman, T. A., Janssens, A. C., de Koning, I., Polman, C. H., Boringa, J. B., & Hintzen, R. Q. (2008). The role of disability and depression in cognitive functioning within 2 years after multiple sclerosis diagnosis. *Journal of neurology*, *255*(6), 910–916. <https://doi.org/10.1007/s00415-008-0814-x>
132. Sirhan, B., Frid, L., & Kalron, A. (2018). Is the dual-task cost of walking and texting unique in people with multiple sclerosis?. *Journal of neural transmission (Vienna, Austria : 1996)*, *125*(12), 1829–1835. <https://doi.org/10.1007/s00702-018-1939-4>
133. Skorve, E., Lundervold, A. J., Torkildsen, Ø., & Myhr, K. M. (2020). A two-year longitudinal follow-up of cognitive performance assessed by BICAMS in newly diagnosed patients with MS. *Multiple sclerosis and related disorders*, *46*, 102577. <https://doi.org/10.1016/j.msard.2020.102577>
134. Souissi, A., Mrabet, S., Ferchichi, W., Gharbi, A., Nasri, A., Djebara, M. B., Kacem, I., & Gouider, R. (2022). Tunisian version of the brief international cognitive assessment for multiple sclerosis: Validation and normative values. *Multiple sclerosis and related disorders*, *58*, 103444. <https://doi.org/10.1016/j.msard.2021.103444>
135. Spedo, C. T., Frndak, S. E., Marques, V. D., Foss, M. P., Pereira, D. A., Carvalho, L.deF., Guerreiro, C. T., Conde, R. M., Fusco, T., Pereira, A. J., Gaino, S. B., Garcia, R. B., Benedict, R. H., & Barreira, A. A. (2015). Cross-cultural Adaptation, Reliability, and Validity of the BICAMS in Brazil. *The Clinical neuropsychologist*, *29*(6), 836–846. <https://doi.org/10.1080/13854046.2015.1093173>
136. Strober, L., Englert, J., Munschauer, F., Weinstock-Guttman, B., Rao, S., & Benedict, R. H. (2009). Sensitivity of conventional memory tests in multiple sclerosis: comparing the Rao Brief Repeatable Neuropsychological Battery and the Minimal Assessment of Cognitive Function in MS. *Multiple sclerosis (Houndmills, Basingstoke, England)*, *15*(9), 1077–1084. <https://doi.org/10.1177/1352458509106615>
137. Trufanov, A., Bisaga, G., Skulyabin, D., Temniy, A., Poplyak, M., Chakchir, O., Efimtsev, A., Dmitriy, T., Odinak, M., & Litvinenko, I. (2021). Thalamic nuclei degeneration in multiple sclerosis. *Journal of clinical neuroscience : official journal of the Neurosurgical Society of Australasia*, *89*, 375–380. <https://doi.org/10.1016/j.jocn.2021.05.043>
138. Tsagkas, C., Geiter, E., Gaetano, L., Naegelin, Y., Amann, M., Parmar, K., Papadopoulou, A., Wuerfel, J., Kappos, L., Sprenger, T., Granziera, C., Mallar Chakravarty, M., & Magon, S. (2022). Longitudinal changes of deep gray matter shape in multiple sclerosis. *NeuroImage. Clinical*, *35*, 103137. <https://doi.org/10.1016/j.nicl.2022.103137>
139. Valdés Cabrera, D., Stobbe, R., Smyth, P., Giuliani, F., Emery, D., & Beaulieu, C. (2020). Diffusion tensor imaging tractography reveals altered fornix in all diagnostic subtypes of multiple sclerosis. *Brain and behavior*, *10*(1), e01514. <https://doi.org/10.1002/brb3.1514>
140. Van Laethem, D., De Cock, A., Van Schependom, J., Benedict, R. H. B., Nagels, G., & D'hooghe, M. (2022). Correlates of patient-reported cognitive performance with regard to disability. *Scientific reports*, *12*(1), 13489. <https://doi.org/10.1038/s41598-022-17649-3>
141. Vanotti, S., Smerbeck, A., Eizaguirre, M. B., Saladino, M. L., Benedict, R. R. H., & Caceres, F. J. (2018). BICAMS in the Argentine population: Relationship with clinical and sociodemographic variables. *Applied neuropsychology. Adult*, *25*(5), 424–433. <https://doi.org/10.1080/23279095.2017.1323751>
142. Vázquez-Marrufo, M., Galvao-Carmona, A., Caballero-Díaz, R., Borges, M., Paramo, M. D., Benítez-Lugo, M. L., Ruiz-Peña, J. L., & Izquierdo, G. (2019). Altered individual behavioral and EEG parameters are related to the EDSS score in relapsing-remitting multiple sclerosis patients. *PloS one*, *14*(7), e0219594. <https://doi.org/10.1371/journal.pone.0219594>
143. Vinciguerra, C., Giorgio, A., Zhang, J., Nardone, V., Brocci, R. T., Pastò, L., Niccolai, C., Stromillo, M. L., Mortilla, M., Amato, M. P., & De Stefano, N. (2021). Peak width of skeletonized mean diffusivity (PSMD) and cognitive functions in relapsing-remitting multiple sclerosis. *Brain imaging and behavior*, *15*(4), 2228–2233. <https://doi.org/10.1007/s11682-020-00394-4>
144. Walker, L. A., Osman, L., Berard, J. A., Rees, L. M., Freedman, M. S., MacLean, H., & Cousineau, D. (2016). Brief International Cognitive Assessment for Multiple Sclerosis (BICAMS): Canadian contribution to the international validation project. *Journal of the neurological sciences*, *362*, 147–152. <https://doi.org/10.1016/j.jns.2016.01.040>
145. Warlop, N. P., Achten, E., Fieremans, E., Debruyne, J., & Vingerhoets, G. (2009). Transverse diffusivity of cerebral parenchyma predicts visual tracking performance in relapsing-remitting multiple sclerosis. *Brain and cognition*, *71*(3), 410–415. <https://doi.org/10.1016/j.bandc.2009.05.004>
146. Welton, T., Constantinescu, C. S., Auer, D. P., & Dineen, R. A. (2020). Graph Theoretic Analysis of Brain Connectomics in Multiple Sclerosis: Reliability and Relationship with Cognition. *Brain connectivity*, *10*(2), 95–104. <https://doi.org/10.1089/brain.2019.0717>
147. Wen, J., Yablonskiy, D. A., Luo, J., Lancia, S., Hildebolt, C., & Cross, A. H. (2015). Detection and quantification of regional cortical gray matter damage in multiple sclerosis utilizing gradient echo MRI. *NeuroImage. Clinical*, *9*, 164–175. <https://doi.org/10.1016/j.nicl.2015.08.003>
148. Wetter, N. C., Hubbard, E. A., Motl, R. W., & Sutton, B. P. (2016). Fully automated open-source lesion mapping of T2-FLAIR images with FSL correlates with clinical disability in MS. *Brain and behavior*, *6*(3), e00440. <https://doi.org/10.1002/brb3.440>
149. Wieder, L., Gäde, G., Pech, L. M., Zimmermann, H., Wernecke, K. D., Dörr, J. M., Bellmann-Strobl, J., Paul, F., & Brandt, A. U. (2013). Low contrast visual acuity testing is associated with cognitive performance in multiple sclerosis: a cross-sectional pilot study. *BMC neurology*, *13*, 167. <https://doi.org/10.1186/1471-2377-13-167>
150. Yaldizli, Ö., Penner, I. K., Frontzek, K., Naegelin, Y., Amann, M., Papadopoulou, A., Sprenger, T., Kuhle, J., Calabrese, P., Radü, E. W., Kappos, L., & Gass, A. (2014). The relationship between total and regional corpus callosum atrophy, cognitive impairment and fatigue in multiple sclerosis patients. *Multiple sclerosis (Houndmills, Basingstoke, England)*, *20*(3), 356–364. <https://doi.org/10.1177/1352458513496880>
151. Yigit, P., Acikgoz, A., Mehdiyev, Z., Dayi, A., & Ozakbas, S. (2021). The relationship between cognition, depression, fatigue, and disability in patients with multiple sclerosis. *Irish journal of medical science*, *190*(3), 1129–1136. <https://doi.org/10.1007/s11845-020-02377-2>
152. Yoshii, F., Takagi, S., Nagata, E., Hasegawa, Y., Kunika, N., Maki, F., Mochizuki, H., Ogino, M., & Kuroiwa, Y. (2015). Cognitive impairment of Japanese multiple sclerosis patients: Follow-up study using BRB-N assessment tool. *Journal of the neurological sciences*, *359*(1-2), 323–327. <https://doi.org/10.1016/j.jns.2015.11.002>
153. Yu, H. J., Christodoulou, C., Bhise, V., Greenblatt, D., Patel, Y., Serafin, D., Maletic-Savatic, M., Krupp, L. B., & Wagshul, M. E. (2012). Multiple white matter tract abnormalities underlie cognitive impairment in RRMS. *NeuroImage*, *59*(4), 3713–3722. <https://doi.org/10.1016/j.neuroimage.2011.10.053>
154. Zheng, P., Pilutti, L. A., DuBose, N. G., & Motl, R. W. (2023). Vascular function and cognition in persons with multiple sclerosis: Preliminary examination. *Multiple sclerosis and related disorders*, *71*, 104578. <https://doi.org/10.1016/j.msard.2023.104578>
155. Zhu, Q., Zheng, Q., Luo, D., Peng, Y., Yan, Z., Wang, X., Chen, X., & Li, Y. (2022). The Application of Diffusion Kurtosis Imaging on the Heterogeneous White Matter in Relapsing-Remitting Multiple Sclerosis. *Frontiers in neuroscience*, *16*, 849425. <https://doi.org/10.3389/fnins.2022.849425>

**Appendix 6, Supplemental table 1: Baseline characteristics of the included studies (attached)**

**Supplemental table 1.** Baseline characteristics of the included studies.

nd: no data; dg: diagnosis; sy: symptom; DD: Disease Duration; EDSS: Expanded Disability Status Scale; ys: years; PMS: Progressive Multiple Sclerosis; PRMS: Progressive-Relapsing Multiple Sclerosis; RMS: Relapsing MS

**Appendix 7, Supplemental figure 1-3: Individual forest plots of the meta-analysis of the study-level correlations stratified by different MS subtypes („Mixed MS”, RRMS)**


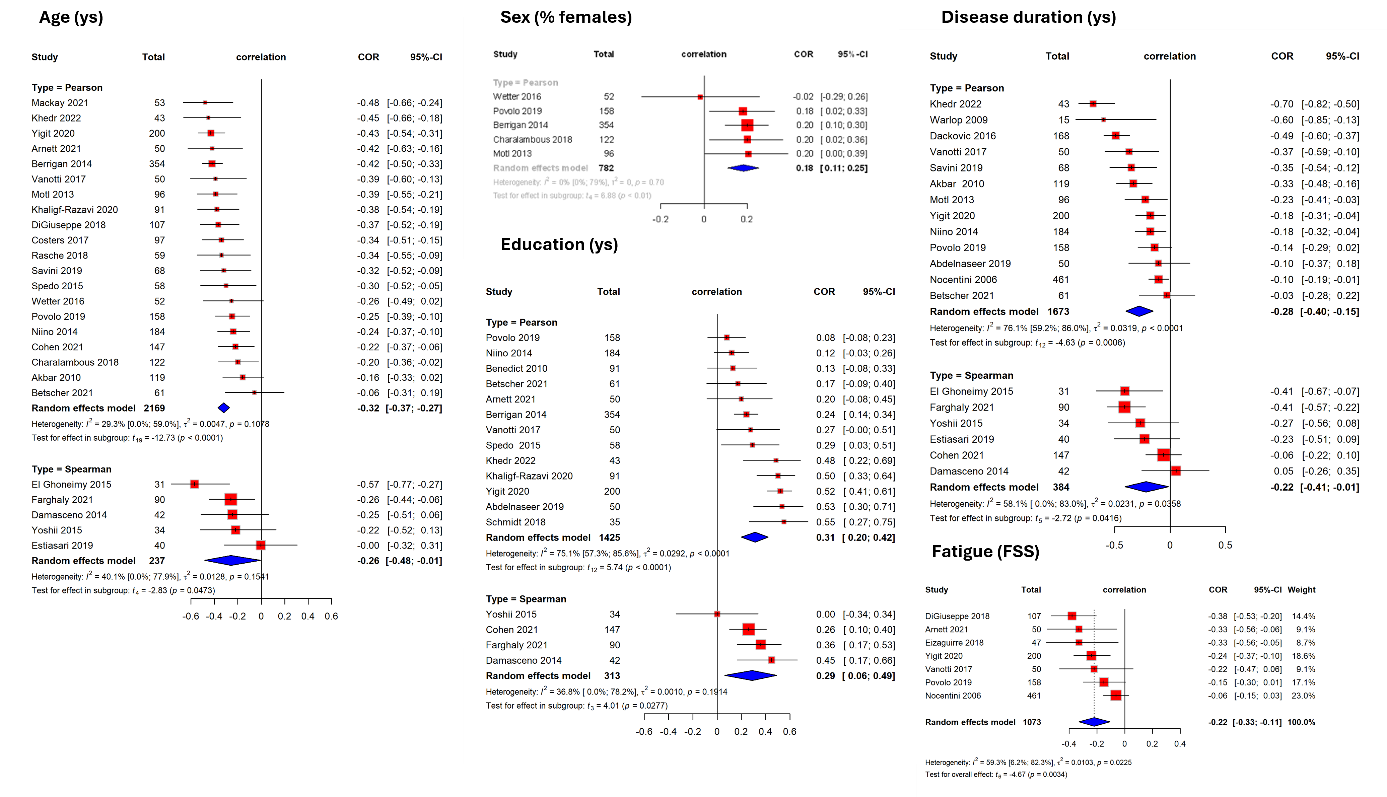


**Supplemental figure 1.** Meta-analysis of the study-level correlations regarding covariates of age (in years), sex (% of females), education (in years), disease duration (in years), and fatigue (FSS score) parameters in the Mixed MS population.


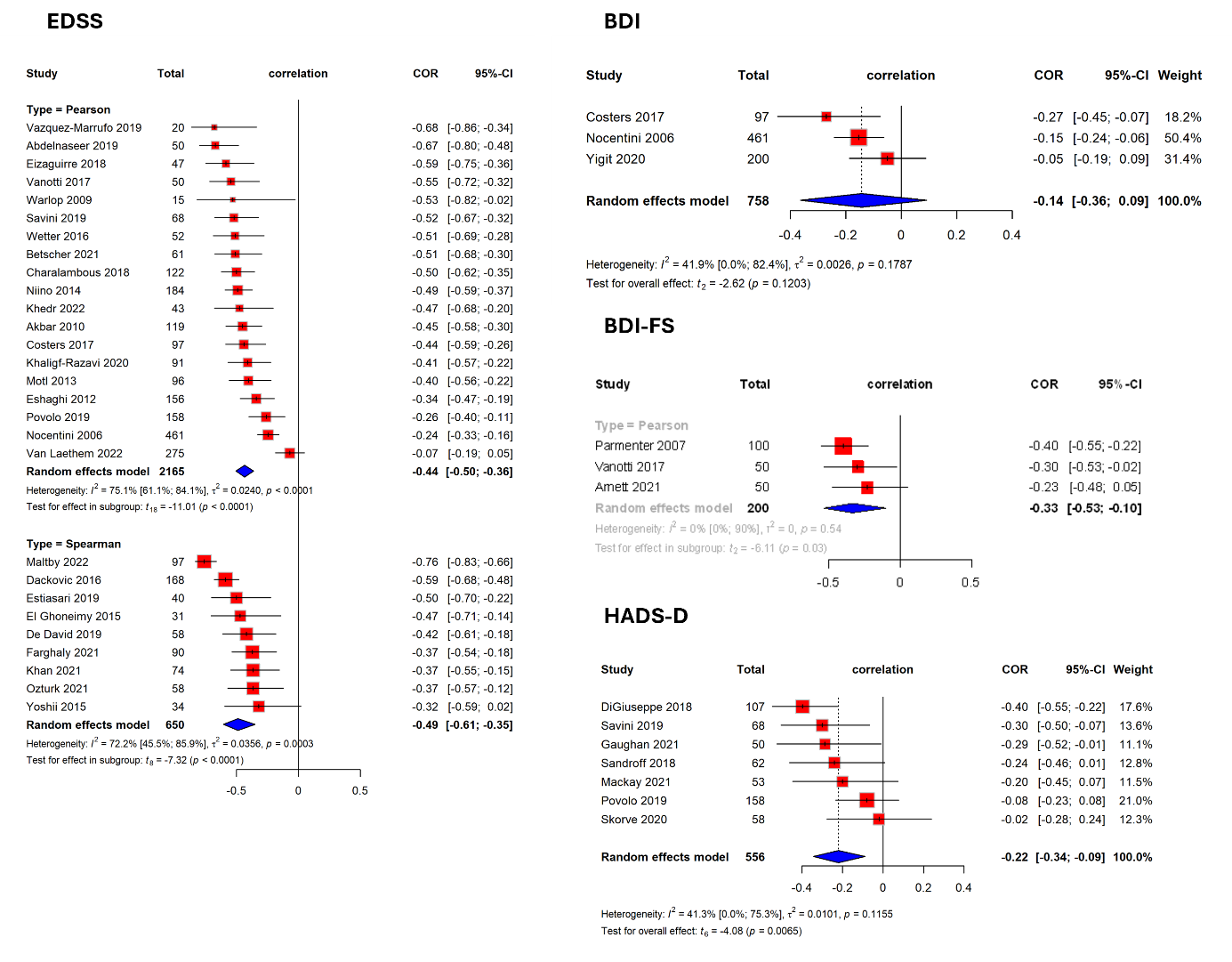


**Supplemental figure 2.** Meta-analysis of the study-level correlations regarding covariates of EDSS, and depression scores (BDI, BDI-FS, HADS-D) in the Mixed MS population.


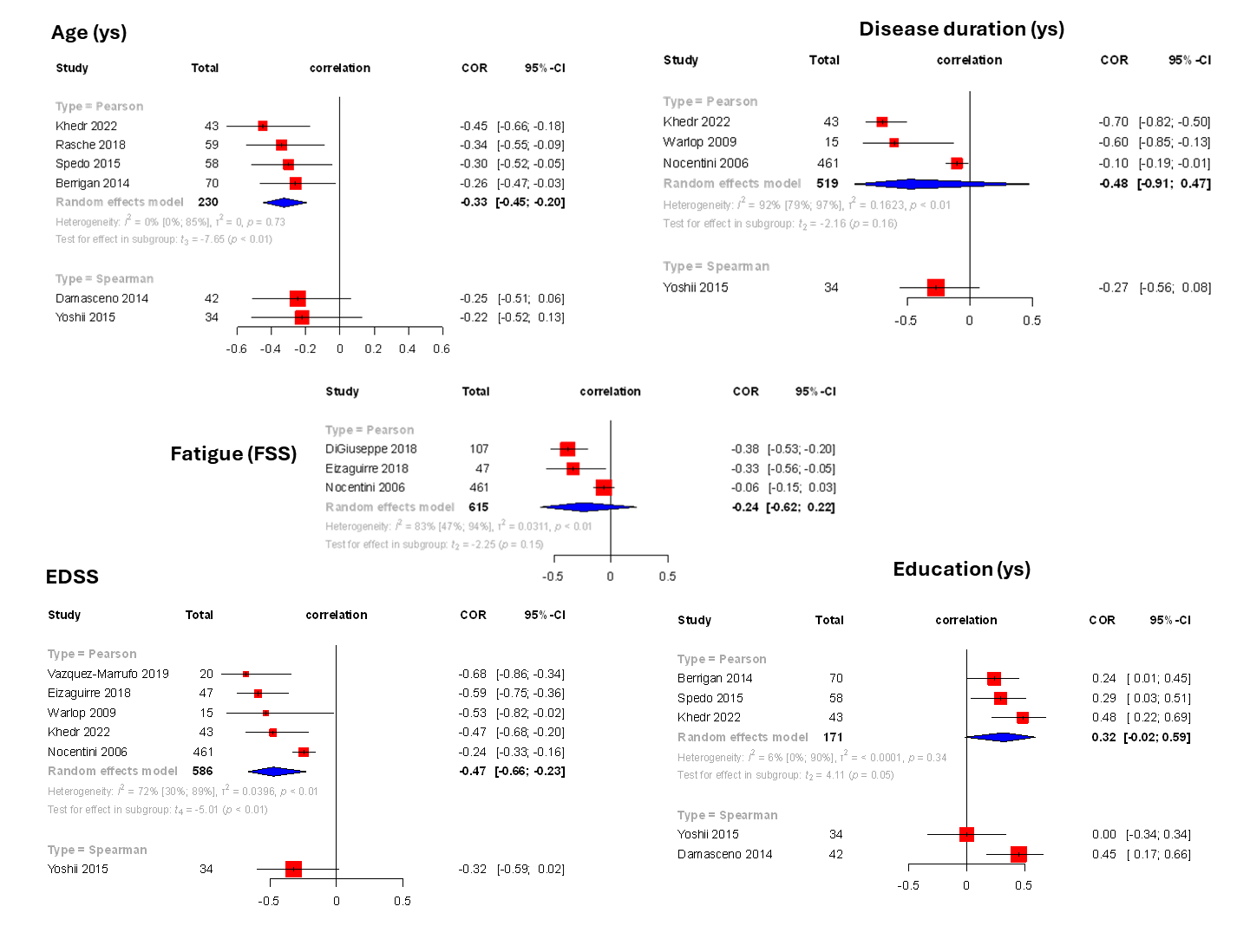


**Supplemental figure 3.** Meta-analysis of the study-level correlations regarding covariates of age (in years), disease duration (in years), fatigue (FSS score), EDSS, and education (in years) parameters in the RRMS population.

**Appendix 8, Supplemental figure 4-7: Individual scatter plots of meta-regressions stratified by different MS subtypes („Mixed MS”, RRMS)**


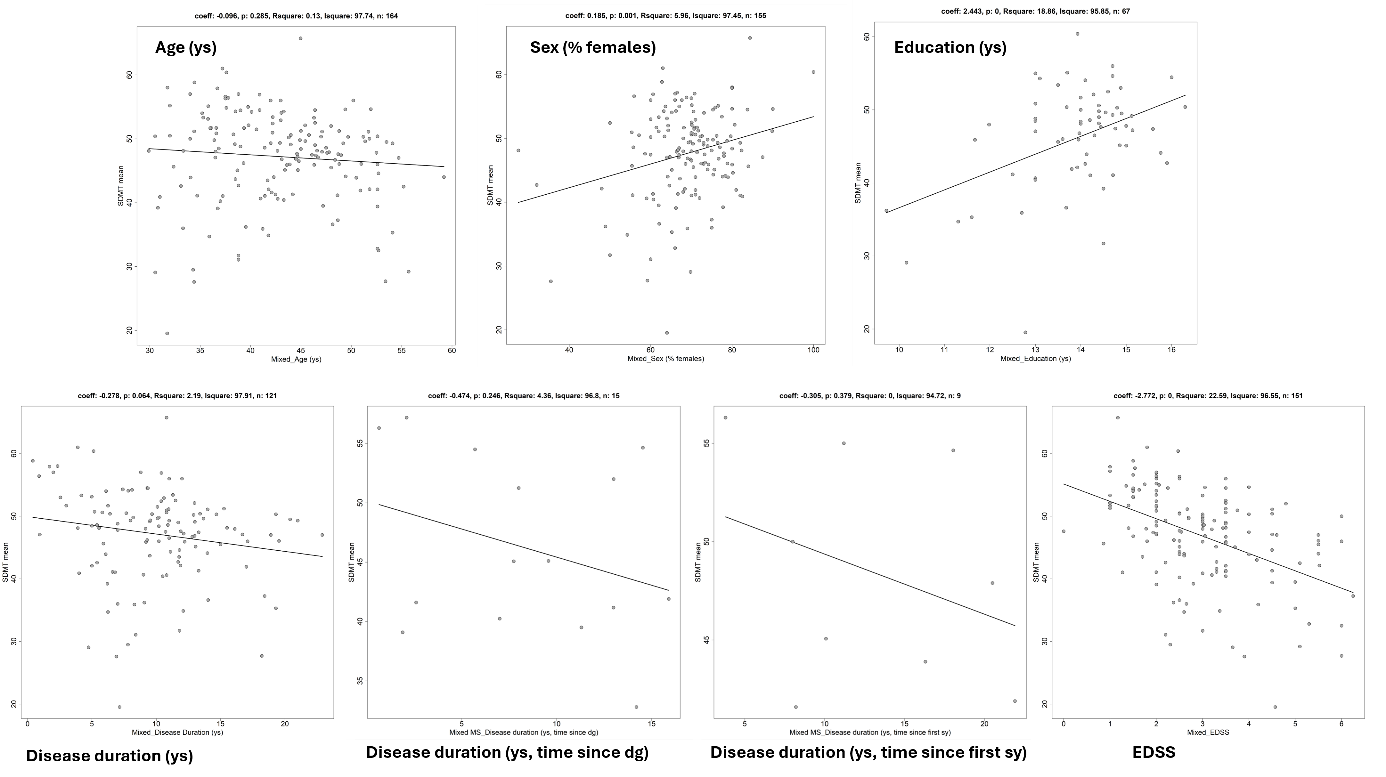


**Supplemental figure 4.** Scatter plots of the meta-regression analyses of age (in years), sex (% of females), education (in years), disease duration (in years), disease duration (time since dg, in years), disease duration (time since first sy, in years), EDSS variables in Mixed MS populations.


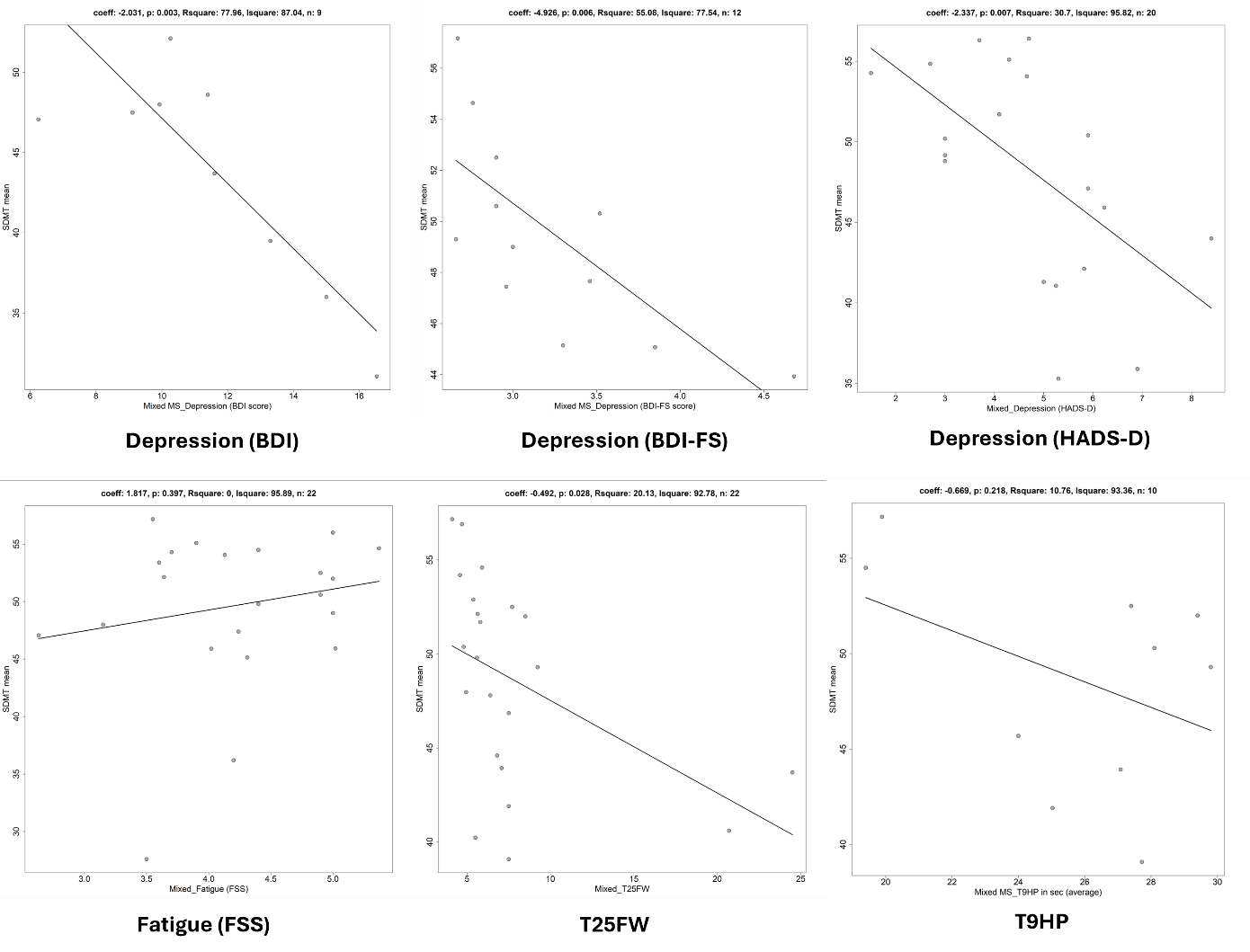


**Supplemental figure 5.** Scatter plots of the meta-regression analyses of depression (BDI, BDI-FS, HADS-D scores), fatigue (FSS score), T25FW, and T9HP variables in Mixed MS populations.


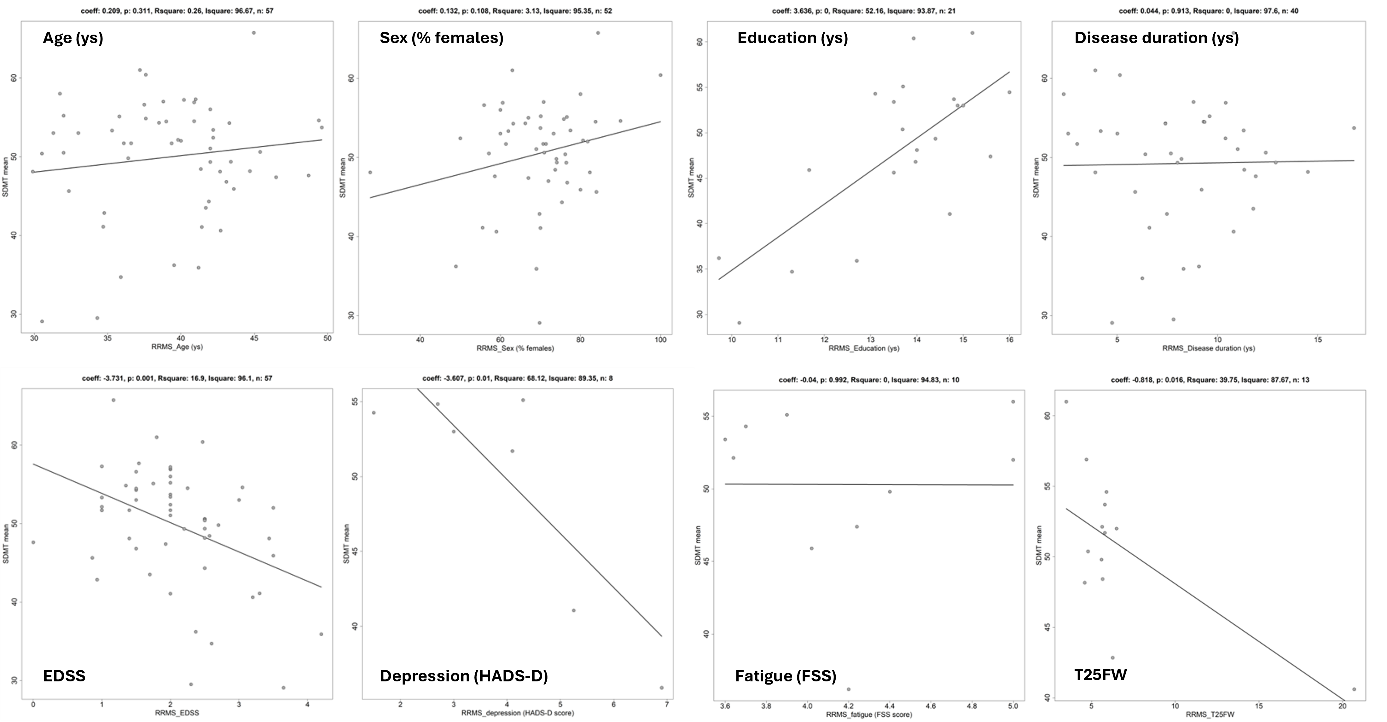


**Supplemental figure 6.** Scatter plots of the meta-regression analyses of age (in years), sex (% of females), education (in years), disease duration (in years), EDSS, depression (HADS-D score), fatigue (FSS), T25FW variables in RRMS populations.

**
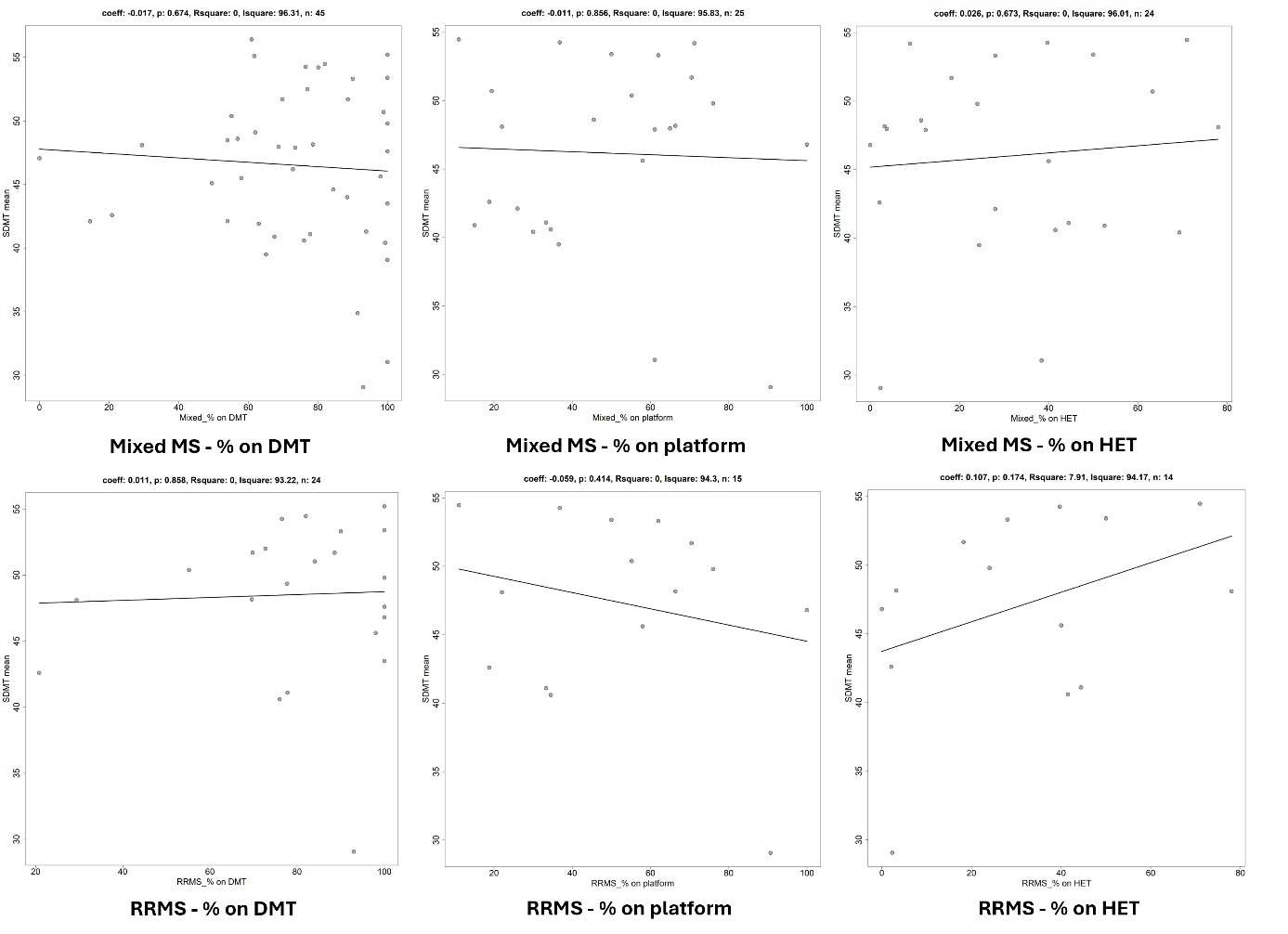
**

**Supplemental figure 7.** Scatter plots of the meta-regression analyses of % on DMT, % on platform, and % on HET variables in Mixed MS and RRMS populations.

**Appendix 9, Supplemetal table 2a-b: Summary table of the study-level multivariate regression model analyses (systematic review part)**

|  | | | Factors in the adjustment | | | | | | | |
| --- | --- | --- | --- | --- | --- | --- | --- | --- | --- | --- |
| Study | **MS subtypes – No. of patients** | **Regression type** | **Age** | **Edu** | **EDSS** | **DD** | **Sex** | **Depr** | **Anx** | **Fat** |
| Benedict et al., 2010 | mixed-91 | forward stepwise | y | **0.76** | n | y | y | n | n | n |
| Betscher et al., 2021 | mixed-61 | multivariate linear | y | y | **-0.51** | y | n | n | n | n |
| Figved et al., 2008 | mixed-78 | multivariate linear | y | y | **-3.34** | n | y | y^*^ | n | n |
| Van Laethem et al., 2022 | mixed-275 | multivariate logistic | **-3,1** | y | **-8.86** | n | y | y^**^ | n | n |

**Supplemental table 2a.** Summary table of the study-level multivariable regression models of the covariates published in the literature – age, education, EDSS, disease duration, sex, depression, anxiety and fatigue scores. Significant results (p<0.05) are highlighted in bold and burgundy.

Edu: education; DD: disease duration; Depr: depression; Anx: anxiety; Fat: fatigue; neg: negative; y: yes; n: no; fem: female; ^*^BDI: Beck Depression Inventory, BDI with items 15,17, 20,21 removed; ^**^The 2-question screening tool for depression in MS

| Study | MS subtypes – No. of patients | Regression type | Other adjusted parameters |
| --- | --- | --- | --- |
| Benedict et al., 2010 | mixed-91 | forward stepwise | IP index  disease course  cognitive reserve measures |
| Betscher et al., 2021 | mixed-61 | multivariate linear | no |
| Figved et al., 2008 | mixed-78 | multivariate linear | NPI (Neuropsychiatric Inventory) |
| Van Laethem et al., 2022 | mixed-275 | multivariate logistic | **MSNQ** |

**Supplemental table 2b.** Summary table of the study-level multivariate regression models of the covariates published in the literature – other adjusted parameters.

IP: Information Processing; MSNQ: Multiple Sclerosis Neuropsychological Questionnaire

**Appendix 10, Supplemental figure 8a-b: Pairwise dependency analysis of the covariates**


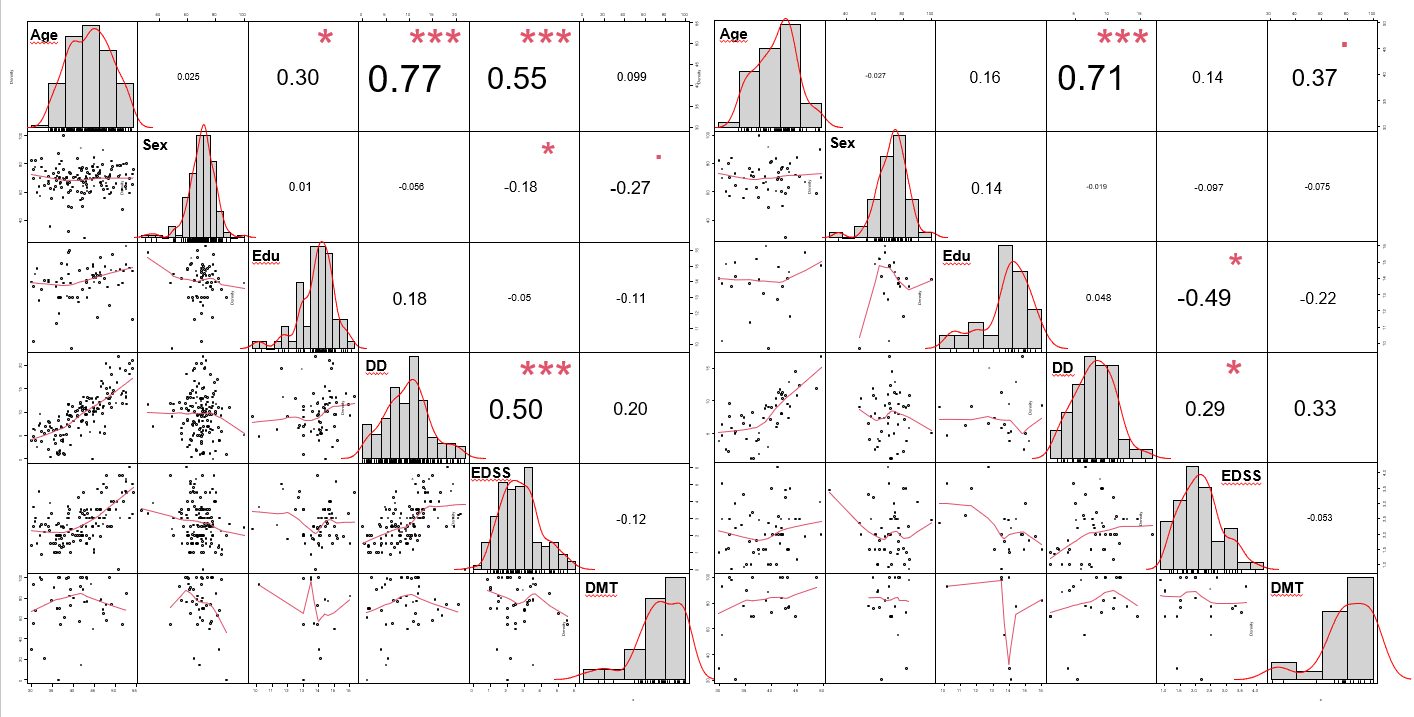


**Supplemental figure 8a-b.** Pairwise dependency matrix of the covariates in mixed (Fig 8a) and in RRMS populations (Fig 8b).

The results of the pairwise dependency analyses. The main diagonal elements contain the histograms of the covariates. Below the main diagonal pairwise scatter plot, visualizations are present while upper the main diagonal Pearson correlations are printed. The absolute value of the correlations represents the strength of the linear relationship between the variables while the sign indicates the direction of the relationship. One, two and three stars indicate p-values less than 0.001, 0.1 and 0.05, respectively. A small rectangle is used to indicate a p-value between 0.05 and 0.1.

DD: disease duration (ys), Edu: education (ys), EDSS: Expanded Disability Status Scale

**Appendix 11, Supplemental table 3: Multivariable regression models based on the results of the pairwise analysis of the covariates**

| **MS subtypes** | **Multivariable regression models** | **Variables** | **n** | **estimate** | **p-value** | **CI** |
| --- | --- | --- | --- | --- | --- | --- |
| **Mixed MS** | **Sex+Edu+Age** | **Sex** | 68 | **0.2011** | **0.0132** | [0.0421; 0,3601] |
|  |  | **Edu** |  | **2.4129** | **0.0003** | [1.1173; 3.7084] |
|  |  | Age |  | 0.0111 | 0.9270 | [-0.2262; 0.2483] |
|  | **Sex+Edu+DD** | **Sex** | 64 | **0.1799** | **0.0269** | [0.0206; 0.3392] |
|  |  | **Edu** |  | **2.7090** | **<0.0001** | [1.4338; 3.9842] |
|  |  | DD |  | -0.1305 | 0.4574 | [-0.4748; 0.2137] |
|  | **Sex+Edu+EDSS** | **Sex** | 63 | **0.1680** | **0.0459** | [0.0030; 0.3330] |
|  |  | **Edu** |  | **2.4995** | **0.0001** | [1.2360; 3.7630] |
|  |  | **EDSS** |  | **-1.5403** | **0.0395** | [-3.0066; -0.0739] |
| **RRMS** | **Sex+Age** | Sex | 52 | 0.1406 | 0.0876 | [-0.0207; 0.3019] |
|  |  | Age |  | 0.1390 | 0.4535 | [-0.2244; 0.5023] |
|  | **Sex+DD** | Sex | 47 | 0.1552 | 0.1272 | [-0.0443; 0.3547] |
|  |  | DD |  | -0.0856 | 0.7603 | [-0.6356; 0.4644] |
|  | **Sex+% on DMT** | Sex | 22 | 0.1224 | 0.4734 | [-0.2122; 0.4570] |
|  |  | % on DMT |  | 0.0296 | 0.6432 | [-0,0958; 0.1551] |
|  | **Sex+EDSS** | Sex | 51 | 0.1057 | 0.1614 | [-0.0422; 0.2535] |
|  |  | **EDSS** |  | **-3.8686** | **0.0003** | [-5.9580; -1.7791] |
|  | **Sex+Edu+Age** | Sex | 21 | 0.1679 | 0.2532 | [-0.1201; 0.4558] |
|  |  | **Edu** |  | **3.1657** | **0.0005** | [1.3956; 4.9357] |
|  |  | Age |  | 0.0236 | 0.9263 | [-0.4757; 0.5229] |
|  | **Sex+Edu** | Sex | 21 | 0.1650 | 0.2447 | [-0.1130; 0.4431] |
|  |  | **Edu** |  | **3.1807** | **0.0002** | [1.4879; 4.8734] |
|  | **Sex+% on DMT+Age** | Sex | 22 | 0.1762 | 0.3132 | [-0.1662; 0.5186] |
|  |  | % on DMT |  | 0.0026 | 0.9689 | [-0.1290; 0.1343] |
|  |  | Age |  | 0.3356 | 0.2261 | [-0.2078; 0.8790] |
|  | **Sex+EDSS+Age** | Sex | 51 | 0.1041 | 0.1625 | [-0.0420; 0.2501] |
|  |  | **EDSS** |  | **-4.1249** | **0.0001** | [-6.2115; -2.0383] |
|  |  | Age |  | 0.2640 | 0.1253 | [-0.0736; 0.6016] |

**Supplemental table 3.** Multivariable regression models, based on the results of the pairwise analysis of the covariates.

Significant results are highlighted in bold and burgundy, results close to significance are highlighted in bold and italics.

n*:* number of the included studies; CI: confidence interval; Edu: education; DD: disease duration; EDSS: Expanded Disability Status Scale; DMT: Disease-Modifying Therapies

**Appendix 12, Supplemental figure 9-10: Assessment of risk of bias for each included study (listed in accordance with the “JBI Quality Assessment Tool for Analytical Cross-Sectional Studies” criteria)**


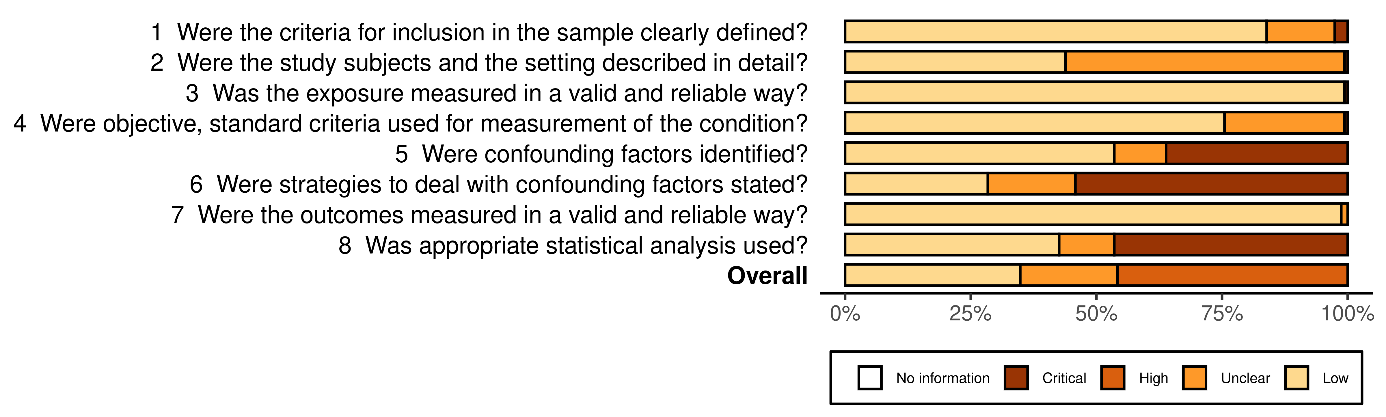


**Supplemental figure 9.** Assessment of risk of bias for each included study (Summary plot) using Risk-of-bias VISualization (robvis) visualization tool:

Quality evaluation based on the "JBI Quality Assessment Tool for Analytical Cross-Sectional Studies" criteria, examines the transparency of evidence synthesis results and findings along 8 aspects.

The first four questions (Q1-Q4) refer to the possibility of **„selection bias” and „performance bias”**.

Since we found 1.13% of „Critical”, nearly a quarter (23.23%) of „Unclear" and 75.64% of „Low” answers in the included articles based on all 4 questions, this corresponds to an approximately **„low risk”** and a low possibility of selection/performance bias – presumably due to the fact that all articles were not RCTs but observational studies (because of the nature of our clinical question).

Q5 and Q6 questions refer to the **„detection bias”** and also address the issue of **„reporting bias”**.

In assessing these two questions, we considered the level of evidence at which the associations between different sociodemographic/clinical parameters and SDMT outcomes were examined in the study: whether mean values were reported, whether direct correlation tests were performed, or whether the highest level of regression analysis was performed: in the latter case, what other relevant parameters were included in the multivariate analyses, or whether only univariate regression analysis was performed. In the case where at least correlational analyses were performed, but with multiple parameters and/or regression analyses, the study was rated "Low".

If there was only one type of correlation or a few, then the study was rated "Unclear" or „Some concerns”, and if only means were reported, then the study was rated as "Critical".

Given that 40,97% of the articles received "Low", 5,16% "Unclear", 8,71% "Some concerns", and 45,16% "Critical" responses, we considered this to be a "high risk", i.e., a possible "detection", to a lower extent "reporting bias".

Q7 question refers to the possibility of **„detection bias”**. In this regard, our study is considered to have a **„very low risk”**: 1.29% of the articles included obtained an "Unclear" answer to this question, all others receiving a "Low" answer. „Detection bias” does not arise in this aspect.

Q8 question is related to the statistical aspect of the included articles. This was primarily aligned with questions 6 and 7, meaning that we evaluated it according to the level of evidence presented in the study. The result is therefore similar to the evaluations for Q6 and Q7, with the distinction that here we also considered the most precise description of the statistical methods. A well-justified statistical method relevant to clarifying the question could have improved the evaluation. Accordingly, the final assessment resulted in 34.84% „Low”, 19.35% „Moderate”, and 45,81% „High”, which collectively corresponds to an overall **„moderate”/”high risk**” rating.

For a summary, based on the above considerations and taking into account all the questions and studies, we assess the risk of bias in our study as **„moderate risk”**.

**
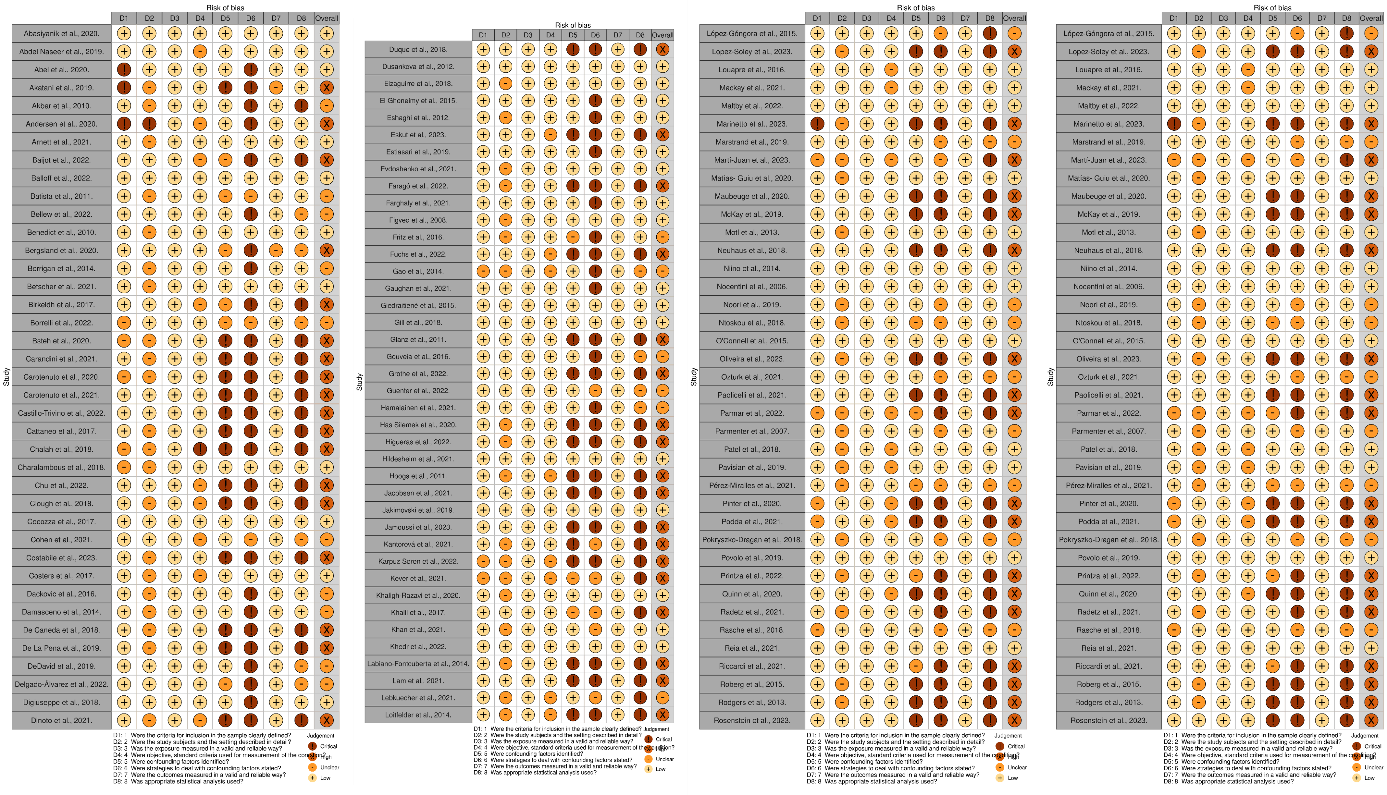
**

**Supplemental figure** **10.** Individual assessments of the risk of bias of all included studies.

**Appendix 13. Ratings of the quality of the evidence (the**[**Oxford Centre for Evidence-based Medicine**](https://www.cebm.net/index.aspx-3Fo-3D5653)**for ratings of individual studies)**:

**
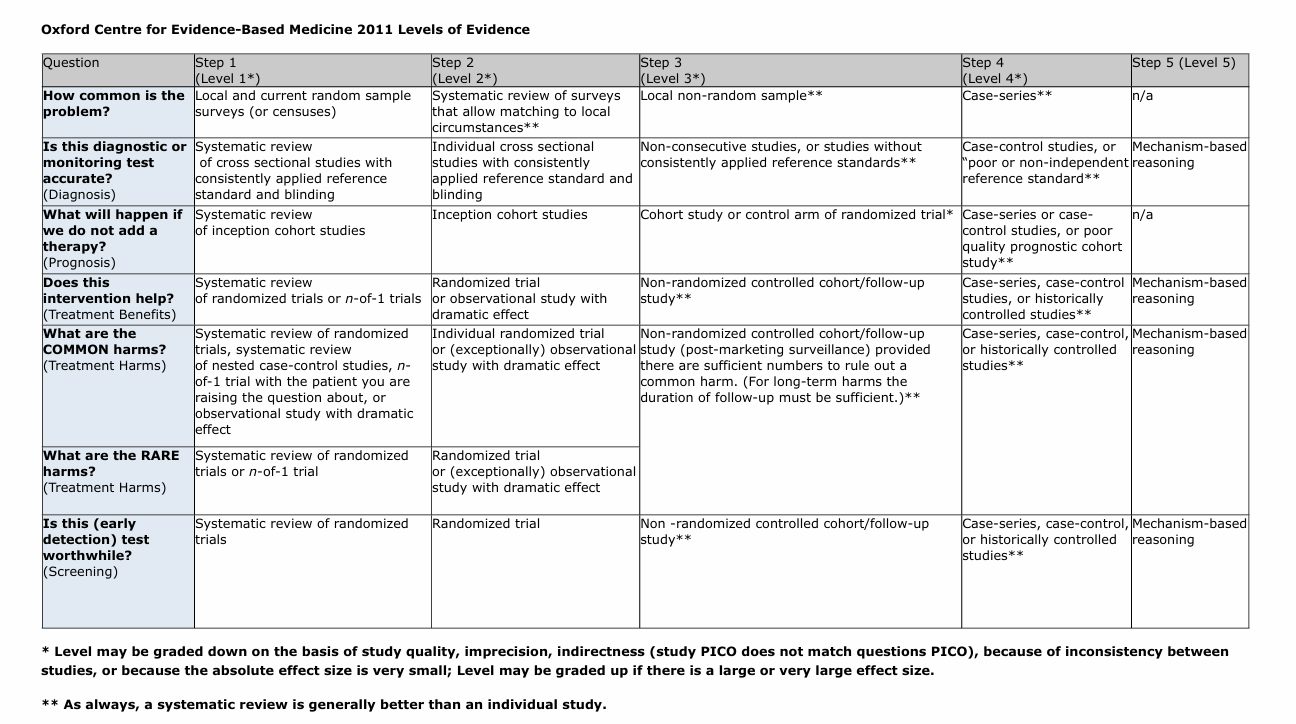
**

| **Quality Rating Scheme for Studies and Other Evidence** | |
| --- | --- |
| **1** | Properly powered and conducted randomized clinical trial; **systematic review with meta-analysis** |
| 2 | Well-designed controlled trial without randomization; prospective comparative cohort trial |
| 3 | Case-control studies; retrospective cohort study |
| 4 | Case series with or without intervention; cross-sectional study |
| 5 | Opinion of respected authorities; case reports |
