# Supplementary material for: Cognitive Impairment in Multiple Sclerosis: The Role of Clinical and Sociodemographic Factors ‐ A Systematic Review and Meta‐Analysis
Source: Ann Clin Transl Neurol. 2025 Sep 24;13(1):58–70. doi: 10.1002/acn3.70172 (PMC12790164; doi:10.1002/acn3.70172)
Supplement: Supplementary file 2 — Table S1: Baseline characteristics of the included studies. [file ACN3-13-58-s001.docx]

| **Supplemental table 1. Baseline characteristics of the included studies** | | | | |  |
| --- | --- | --- | --- | --- | --- |
|  |  |  |  |  |  |
| **First Author, year of publication, level of evidence** | **MS subtypes (% CIS, RRMS, PPMS, SPMS, additional inclusion criteria)** | **No of patients** | **Patient characteristics (female%, age, education, EDSS, DD) mean/*median* (SD) [range or IQR]** | **SDMT raw scores mean/*median* (SD) [range or IQR]** |  |
|  |  |  |  |  |  |
|  |  |  |  |  |  |
| **Abasiyanik et al., 2020 meta-regression** | **Mixed 78,42% RRMS 5,62% PPMS 15,95% SPMS inclusion 18-70ys** | 445 | Female%: 67,2 Age: 38,5 (11,7) EDSS: 2,6 (2,2) DD: 9,5 (8,3) | 43,7 (14) |  |
| **Abdelnaseer et al., 2019 meta-regression, correlation** | **Mixed (nd) inclusion 20-45ys** | 50 | Female%: 64 Age: 31,72 (7,03) Education: 12,78 (3,09) EDSS: 4,57 (2,16) DD: 7,15 (5,18) | 19,54 (9,44) |  |
| **Abel et al., 2020 meta-regression** | **Mixed 52% RRMS 16% PPMS 32% SPMS** | 73 | Females%: 66 Age: 50,2 (10,7) [26-65] Education: 14,7 (2,2) [12-22] EDSS: 3,5 [1-8,5] DD: *12* [0,3-48] | 56 [7-88] |  |
| **Akatani et al., 2019 meta-regression** | **Mixed 93% RRMS 7% SPMS** | 44 | Females%: 77 Age: 41,4 (8,8) EDSS: 3,03 (1,95) DD: 10,9 (7,7) | 48,6 (15,7), *52,5* [0-72] |  |
| **Akbar et al., 2010 meta-regression, correlation** | **Mixed 68% RRMS 8,4% PPMS 21,8% SPMS 1,68% PRMS** | 119 | Female%: 75,63 Age: 44,7 (8,5) Education: 15 (2,2) EDSS: 2,5 [0-8,5] DD (time since dg): 9,57 (7,72) | 45,1 (11,6) |  |
| **Andersen et al., 2020 meta-regression** | **Mixed 65% RRMS 35% PPMS** | 40 | Age: *46,3* (11,5) EDSS: 3 [0-7] | 53,3 (11,8) |  |
| **Andersen et al., 2020 meta-regression** | **RRMS 100% RRMS** | 26 | Age: *40,2* (8,9) EDSS: 2 [0-7] | 57,2 (11) |  |
| **Andersen et al., 2020 meta-regression** | **PPMS 100% PPMS** | 14 | Age *57,7* (5) EDSS: 4 [3-6,5] | 45,9 (9,8) |  |
| **Arnett et al., 2021 meta-regression, correlation** | **Mixed 58% RRMS 14% PPMS 28% SPMS** | 50 | Female%: 80 Age: 51,94 (9,28) Education: 14,7 (2,1) EDSS: 4 [0-7,5] DD (time since dg): 14,54 (8,67) DD (time since first sy): 18,04 (9,25) | 54,64 (13,22) |  |
| **Baijot et al., 2022 meta-regression** | **Mixed (nd)** | 61 | Female%: 55,74 Age: 47,5 (9,7) Education: 14,2 (2,5) EDSS: 3 [2-4] DD: 5,4 (8,4) | 48,6 (11,2) |  |
| **Balloff et al., 2022 meta-regression** | **RRMS 100% RRMS** | 63 | Female%: 67 Age: *39* [20-61] Education: *16* [8-22] EDSS: 1,5 [0-7,5] DD: *9,35* [0-30] | *55*[19-84] |  |
| **Batista et al., 2011 meta-regression** | **Mixed 68,6% RRMS 31,4% SPMS** | 86 | Female%: 71 Age: 45,8 (9,3) Education: 14,4 (2,2) EDSS: 3,5 [0-6,5] DD: 10,8 (7,6) | 50,6 (15,8) |  |
| **Bellew et al., 2022 meta-regression, correlation** | **Mixed 80,2% RRMS 4,2% PPMS 9,4% SPMS 6,2% unknown** | 192 | Female%: 69,3 Age: 42,92 (11,48) [22-81] DD: 9,53 (8,18) | 49,35 (13,64) [13-81] |  |
| **Benedict, et al., 2010 meta-regression, correlation, multivariate regression model** | **Mixed 78% RRMS 3,3% PPMS 18,7% SPMS** | 91 | Female%: 70 Age: 44,8 (8,8) Education: 14,3 (2) DD: 11 (8,3) | 46,5 (12,4) |  |
| **Bergsland et al., 2020 meta-regression** | **Mixed 62,96% RRMS 5,56% PPMS 31,48% SPMS inclusion 18-75ys** | 108 | Female%: 72,2 Age: 51,2 (10,4) Education: 14,8 (2,2) EDSS: *3* [1,5-5,9] DD: 19,3 (10) | 50,3 (15,1) |  |
| **Berrigan et al., 2014 correlation** | **Mixed (nd)** | 354 | Female%: 76,84 Age: 49,09 (10,46) [23-76] Education: 14,21 (2,2) [6-21] EDSS: 3,28 (2,17) [0-9] DD: 15,18 (9,86) [0-50] | nd |  |
| **Berrigan et al., 2014 correlation** | **RRMS 100% RRMS** | 70 | Female%: 81,43 Age: 40,34 (8,78) [23-59] Education: 14,81 (1,98) [12-21] EDSS: 1,83 (1,18) [0-6] DD: 4,37 (3,02) [0,08-10,42] | nd |  |
| **Betscher et al., 2021 meta-regression, correlation, multivariate regression model** | **Mixed 73,77% RRMS 6,56% PPMS 19,67% SPMS** | 61 | Female%: 74 Age: *39* [28-49] Education: *13* [12-17] EDSS: *3,5* [2-4,5] DD: *7* [3-13] | 48,8 (12,1) |  |
| **Betscher et al., 2021 meta-regression** | **RRMS 100% RRMS** | 45 | Female%: 73,3 Age: *33* [27-46] Education: *15* [12-17] EDSS: 3 [2-4] DD: *5* [2,5-9] | *53* [46-60] |  |
| **Betscher et al., 2021 meta-regression** | **SPMS 100% SPMS** | 12 | Female%: 75 Age: *47* [44-52,5] Education: *12* [11,5-17] EDSS: 4,75 [4,25-6,5] DD: *19,5* [15-25,5] | *43* [30-47] |  |
| **Birkeldh et al., 2017 meta-regression** | **RRMS 100% RRMS** | 391 | Female%: 70,8 Age: 38,8 (9,5) EDSS: 2 [1,25-2,75] DD: 8,8 (7,1) | *57* [49,5-64,5] |  |
| **Birkeldh et al., 2017 meta-regression** | **PPMS 100% PPMS** | 19 | Female%: 57,9 Age: 51,8 (13,9) EDSS: *6* [5-7] DD: 11,2 (7,9) | *50* [44,5-55,5] |  |
| **Birkeldh et al., 2017 meta-regression** | **SPMS 100% SPMS** | 136 | Female%: 66,9 Age: 54,7 (10,1) EDSS: *5,5* [4,25-6,75] DD: 22,9 (10) | *47* [33,5-60,5] |  |
| **Borelli et al., 2022 meta-regression** | **Mixed 9,1% CIS 69,7% RRMS 6,1% PPMS 15,1% SPMS** | 66 | Female%: 60,6% Age: 44,2 (14,8) EDSS: *3,5* [1,5-5,5] | 41,3 (14,4) |  |
| **Bsteh et al., 2020 meta-regression** | **Mixed 100% relapsing mixed population** | 171 | Female%: 73,1 Age: 35,2 (9,6) EDSS *1,5* [0-6,5] DD: 6,1 (6,5) | 54 (10,3) |  |
| **Carandini et al., 2021 meta-regression** | **RRMS 100% RRMS inclusion 18-55ys, EDSS<6,5** | 68 | Female%: 63,2 Age: 43,3 (7,7) EDSS: *1,5* [1-3] DD: 7,4 (6,5) | *49* [48,2-64,7] |  |
| **Carotenuto et al., 2020 meta-regression** | **RRMS 100% RRMS** | 29 | Female%: 59 Age: 42,7 (8,2) EDSS: 3,2 (1,3) DD: 10,8 (1,5) | 40,6 (9,8) |  |
| **Carotenuto et al., 2021 meta-regression** | **Mixed 72% RRMS 2,67% PPMS 24% SPMS** | 150 | Female%: 60,67 Age: 43,3 (11,5) [18-65] Education: 13 [0-18] EDSS: *3,5* [0-7,5] DD: *10,5* [0-40] | 40,42 (18,51) |  |
| **Castillo-Trivino et al., 2022 meta-regression** | **RRMS 100% RRMS early MS** | 189 | Female%: 71,4 Age: 36,1 (9,4) EDSS: *1* [0-2] DD (time since dg): 1,1 (0,8) DD (time since first sy): 1,2 (0,8) | 51,7 (14,7) |  |
| **Cattaneo et al., 2017 meta-regression** | **Mixed 33% RRMS 10% PPMS 57% SPMS** | 98 | Female%: 59,2 Age: 53,4 (11,3) [25-82] EDSS: 6 (1,7) [1,5-8,5] DD: 18,2 (11,2) [1-47] | 27,7 (11,2) [13-59] |  |
| **Chalah et al., 2018 meta-regression** | **Mixed 28% RRMS 36% PPMS 46% SPMS** | 50 | Female%: 48 Age: 51,82 (12,72) [31-73] Education: 13,92 (2,02) [11-18] EDSS: 5,52 (1,64) [2-7,5] DD: 11,88 (6,03) [3-25] | 42,12 (14,08) [26-64] |  |
| **Charalambous et al., 2018 meta-regression, correlation** | **Mixed 47,54% RRMS 22,95% PPMS 29,5% SPMS** | 122 | Female%: 70,49 Age: 48 (11) EDSS: *5,5* [0-8,5] DD: 15 (10) | 45,5 (13,27) |  |
| **Charalambous et al., 2018 meta-regression** | **RRMS 100% RRMS** | 58 | Female%: 68,97 Age: 42 (10) EDSS: *2* [0-7] DD: 11 (8) | 51,04 (14,28) |  |
| **Charalambous et al., 2018 meta-regression** | **PPMS 100% PPMS** | 28 | Female%: 64,29 Age: 52 (9) EDSS: *6* [3-8] DD: 14 (7) | 42,86 (9,46) |  |
| **Charalambous et al., 2018 meta-regression** | **SPMS 100% SPMS** | 36 | Female%: 77,78 Age: 53 (7) EDSS: *6,5* [4-8,5] DD: 22 (10) | 39 (10,88) |  |
| **Chu et al., 2022 meta-regression** | **Mixed 94,5% RRMS 5,5% PPMS early MS** | 384 | Female%: 68,8 Age: 37,8 (10,4) EDSS: *2* [0-6] DD: 0,9 (0,5) | 56,4 (11,1) |  |
| **Clough et al., 2018 meta-regression** | **RRMS 100% RRMS** | 45 | Female%: 84,4 Age: 44,97 (1,74) EDSS: 1,17 (1,53) DD: 10,8 (6,33) | 65,73 (2,32) |  |
| **Cocozza et al., 2017 meta-regression** | **PPMS 100% PPMS inclusion 18-70ys, EDSS≤7** | 47 | Female%: 57,45 Age: 52,6 (11,1) [31-69] Education: 15,4 (3,6) EDSS: *4,5* [1-6,5] DD: 10,1 (7,1) | 39,4 (14,9) |  |
| **Cocozza et al., 2017 meta-regression** | **SPMS 100% SPMS inclusion 18-70ys, EDSS≤7** | 35 | Female%: 68,57 Age: 52,6 (8,5) [29-66] Education: 14,7 (3,6) EDSS: *6* [2-7] DD: 17,1 (11) | 46 (16,4) |  |
| **Cohen et al., 2021 meta-regression, correlation** | **Mixed (nd)** | 147 | Female%: 71,4 Age: 47,6 (11,1) Education: *15* [13-16] DD: 7 [3-13] | 47,8 (12,4) |  |
| **Costabile et al., 2023 meta-regression** | **Mixed 84,2% RRMS inclusion 18-70ys** | 139 | Female%: 70 Age: 36,6 (10,7) [19-64] Education: 13 (4) [4-21] EDSS: *2* [0-7,5] | 50,85 (13,98) |  |
| **Costers et al., 2017 meta-regression, correlation** | **Mixed 84% RRMS 4% PPMS 12% SPMS** | 97 | Female%: 70,1 Age: 45,42 (9,24) Education: 14,28 (1,86) EDSS: 3,5 (2,5) DD: 12,97 (7,16) | 52,11 (13,11) |  |
| **Dackovic et al., 2016 EDSS, DD correlation data only** | **Mixed 22% CIS 38,7% RRMS 20,8% PPMS 18,5% SPMS** | 168 | Female%: 63,7 | nd |  |
| **Damasceno et al., 2014 meta-regression, correlation** | **RRMS 100% RRMS** | 42 | Female%: 76,2 Age: 30,52 (6,6) Education: 13,69 (1,83) EDSS: *2,5* [0-4] DD: 12,97 (7,16) | 50,38 (13,31) |  |
| **De Caneda et al., 2018 meta-regression** | **RRMS 100% RRMS** | 40 | Female%: 27,5 Age: 42,67 [21-67] EDSS: 3,44 (1,28) | 48,1 (18,5) |  |
| **De David et al., 2019 meta-regression, correlation** | **Mixed (nd)** | 58 | Age: *37* [18-69] EDSS: 2,5 [1-4,5] DD (time since dg): *7* [4-11] | *39* [31,5-50] |  |
| **De La Pena et al., 2019 meta-regression** | **Mixed 71,43% RRMS 28,57% SPMS early MS** | 28 | Female%: 64,3 Age: *45* [39-52] EDSS: *1* [0,3-3,6] DD (time since dg): *8* [5-14] | *51* [42-62] |  |
| **De La Pena et al., 2019 meta-regression** | **RRMS 100% RRMS early MS** | 20 | Age: *41* [37-47] EDSS: *1* [0-1] DD (time since dg): *6* [5-10] | *58* [50-64] |  |
| **De La Pena et al., 2019 meta-regression** | **SPMS 100% SPMS early MS** | 8 | Age: *51* [48-57] EDSS: *4,5* [4-6,3] DD (time since dg): *13* [11-18] | *36* [24-43] |  |
| **Delgado-Álvarez et al., 2022 meta-regression** | **RRMS 100% RRMS** | 115 | Female%: 67 Age: 46,49 (10,082) Education: 15,59 (3,384) EDSS: 1,93 (1,954) | 47,39 (13,38) |  |
| **DiGiuseppe et al., 2018 meta-regression, correlation** | **RRMS 100% RRMS newly diagnosed MS** | 107 | Female%: 76,6 Age: 35,8 (9,5) Education: 13,7 (2,3) EDSS: *1,75* [0-5] | 55,1 (11,4) |  |
| **Dinoto et al., 2021 meta-regression** | **RRMS 100% RRMS EDSS≤ 4, DD≤ 10 years** | 44 | Female%: 61,4 Age: 39,36 (11,1) EDSS: *1* [0-4] DD: 3 [0-10] | 51,69 (9,52) |  |
| **Duque et al., 2008. meta-regression** | **Mixed 34% CIS 47,8% RRMS 9,1% PPMS 9,1% SPMS** | 44 | Female%: 68,18 Age: 36 (9,43) Education: *14* [7-28] EDSS: *2* [0-7] DD: 6,25 [1-36] | 51,68 (14,31) |  |
| **Dusankova et al., 2012 meta-regression** | **Mixed 68% RRMS 3% PPMS 26% SPMS 4% PRMS** | 367 | Female%: 68 Age: 34 (10) Education: 14 (3) EDSS: 3 (1,5) DD (time since first sy): 8 (7) | 50 (13) |  |
| **Eizaguirre et al., 2018 correlation** | **RRMS 100% RRMS** | 47 | Female%: 66 Age: 39,04 (13,17) [14-67] Education: 13 (3,87) [3-22] EDSS: 2,78 (1,81) [0-7] DD: 8,07 (6,26) [1-27] | nd |  |
| **El Ghoneimy et al., 2015 meta-regression, correlation** | **Mixed 74,19% RRMS 25,81% SPMS inclusion 18-45ys** | 31 | Female%: 35,48 Age: 34,4 (8,5) EDSS: 3,9 (1,6) [2-6,5] DD: 6,9 (4,9) [2-20] | 27,6 (15,5) [2-60] |  |
| **Eshaghi et al., 2012 meta-regression, correlation** | **Mixed 81% RRMS 18% SPMS** | 156 | Female%: 71 Age: 34,05 (9,09) Education: 14,13 (2,97) EDSS: *2,5*  DD: 6,07 (5,08) | 43,93 (16,55) |  |
| **Eskut et al., 2023 meta-regression** | **RRMS 100% RRMS** | 47 | Female%: 48,9 Age: 39,53 (9,07) *39* [23-63] Education: 9,72 (3,57) *11* [5-16] EDSS: 2,37 (1,69) 2 [0-6,5] DD: 9,07 (6,23) 8 [1-22] | 36 [12,83) *36* [12-62] |  |
| **Estiasari et al., 2019 meta-regression, correlation** | **Mixed 77,5% RRMS 22,5% SPMS** | 40 | Female%: 82,5 Age: *31* [20-61] EDSS: *3* [1-7,5] DD: 4 [0,1-15] | 40,9 (14,8) |  |
| **Evdoshenko et al., 2021 meta-regression, correlation** | **Mixed 85,7% RRMS 14,3% SPMS** | 98 | Female%: 70,4 Age: 38,44 (11,47) Education: 15,12 (2,79) EDSS: *3* [1-6,5] DD: 9,5 (7,44) | 49,16 (13,42) |  |
| **Faragó et al., 2022 meta-regression** | **RRMS 100% RRMS** | 53 | Age: 41,69 (10,96) EDSS: 1,7 (1,65) DD: 11,78 (9,85) | 43,5 (11,4) |  |
| **Farghaly et al., 2021 meta-regression, correlation** | **Mixed 84,5% RRMS 2,2% PPMS 13,3% SPMS inclusion 18-55ys** | 90 | Female%: 77,78 Age: 30,8 (6,7) [19-52] Education: 14,5 (2,6) [8-10] EDSS: 2,8 (1,8) [1-6,5] DD: 6,2 (5,8) | 39,2 (13,3) |  |
| **Figved et al., 2008 meta-regression, correlation, multivariate regression model** | **Mixed 91% RRMS (of whom 13% had converted to SPMS) 9% PPMS** | 78 | Female%: 68 Age: 42,1 (9,9) EDSS: 3,3 (1,5) DD (time since dg): 2,6 (0,9) DD (time since first sy): 8,2 (7,3) | 41,6 (12,9) |  |
| **Fritz et al., 2016 meta-regression** | **RRMS 100%RRMS inclusion EDSS 1-6,5** | 29 | Female%: 58,62 Age: 48,7 (11,5) EDSS: *4* [1-6,5] DD: 11,9 (8,7) | 47,6 (12,5) |  |
| **Fuchs et al., 2022 meta-regression** | **Mixed 82,1% RRMS 17,9% progressive MS** | 740 | Female%: 75,3 Age: 44,9 (10,3) *45* [38-52] [18-88] Education: 14,4 (2,4) *14* [12-16] [7-24] EDSS: 3 (1,7) *2,5* [1,5-4] [0-9] DD: 10,2 (8,2) *8* [4-15] [0,17-52] | 49,8 (12,8) *50* [41-59] [0-98] |  |
| **Gao et al., 2014 meta-regression** | **Mixed 7,2% CIS 36,14% RRMS 34,93% PPMS 20,48% SPMS 1,2% RIS** | 83 | Female%: 55,42 Age: [25-70] EDSS: *3,5* [0-7,5] | *45,5* [12-82] |  |
| **Gaughan et al., 2021 meta-regression, correlation** | **Mixed 62% RRMS** | 50 | Age: 44 EDSS: *2* [1-3,6] DD: 13 | 49,1 (12,3) |  |
| **Giedraitiené et al., 2015 meta-regression** | **Mixed 4% CIS 88% RRMS 2% PPMS 6% SPMS** | 50 | Female%: 32 Age: 38,8 (10,2) Education: 15,9 (2,8) EDSS: 3,3 (1,3) DD: 11,7 (9,2) | 42,7 (13,9) |  |
| **Gill et al., 2018 meta-regression** | **Mixed 71,9% RRMS 4,7% PPMS 23,4% SPMS inclusion 18-59ys** | 128 | Female%: 74,21 Age: 46,32 (8,23) Education: 13,95 (2,4) EDSS: *3* [0-7] DD: 12,21 (7,99) | 45,92 (11,74) |  |
| **Glanz et al., 2011 meta-regression** | **Mixed 23,33% CIS 76,67% RRMS early MS, 18-55ys** | 90 | Female%: 80 Age: 36,7 (8,6) EDSS: *1* [0-5,5] DD: 1,7 (1,6) | 57,9 (9,8) |  |
| **Gouveia et al., 2016 meta-regression** | **PPMS 100% PPMS** | 55 | Female%: 56,4 Age: 52,7 (9,1) Education: *9* [4-17] EDSS: *6* [4-7,5] DD: *42* [35-53] | 32,5 (16,7) |  |
| **Grothe et al., 2022 meta-regression** | **Mixed 89,1% RRMS 1% PPMS 9,9% SPMS** | 101 | Female%: 73,26 Age: 46,1 (12,21) EDSS: 2,54 (1,88) *2* DD: 10,72 (6,75) | 47,49 (13,16) |  |
| **Guenter et al., 2022 meta-regression** | **Mixed 64,5% RRMS 8,6% PPMS 26,8% SPMS** | 93 | Female%: 72 Age: *41* [33-50] [20-67] Education: *13* [12-17] [8-18] DD: *9* [4-16] [0,1-44] | *41* [31-50] |  |
| **Hämäläinen et al., 2021 meta-regression, correlation** | **Mixed 62% RRMS 38% progressive MS** | 65 | Female%: 71 Age: 50,9 (8,8) Education: 13,8 (9,8) DD (time since dg): 15,9 (9,8) DD (time since first sy): 21,9 (11,2) | 41,9 (11,8) |  |
| **Has Silemek et al., 2020 meta-regression** | **RRMS 100% RRMS inclusion EDSS 0-4** | 33 | Female%: 60,6 Age: 40,9 (9,7) EDSS: *2* [0-4] DD: 10,4 (8,1) | 56,9 (9,5) |  |
| **Higueras et al., 2022 meta-regression** | **Mixed 90,4% RRMS 4,3% PPMS 5,3% SPMS** | 302 | Female%: 64,2 Age: 42,3 (10,1) EDSS: 2,6 (1,9) [0-6] | 44 (12) |  |
| **Hildesheim et al., 2021 meta-regression** | **Mixed 64,3% RRMS 35,7% SPMS inclusion 20-65ys** | 84 | Female%: 66,7 Age: 46,6 (8,8) [41-53] Education: 14,4 (2,3) [12,25-16] EDSS: *3,5* [2,5-6] DD: 15,5 (9,3) [8,25-22] | 48,15 (15,49) |  |
| **Hildesheim et al., 2021 meta-regression** | **RRMS 100% RRMS** | 54 | Female%: 74,1 Age: 43,4 (8,4) [36-50] Education: 14,4 (2,3) [12-16] EDSS: *2,5* [1,5-4] DD: 12,9 (8,1) [6-17] | 49,35 (15,08) |  |
| **Hildesheim et al., 2021 meta-regression** | **SPMS 100% SPMS** | 30 | Female%: 53,3 Age: 52,4 (6,2´) [49-57] Education: 14,5 (2,3) [13-16] EDSS: *6* [5-6,5] DD: 20,3 (9,6) [11-29] | 46 (16,25) |  |
| **Hoogs et al., 2011 meta-regression** | **Mixed 71,21% RRMS 28,78% SPMS** | 132 | Female%: 73 Age: 46,4 (10,3) Education: 14,4 (2,1) EDSS: *3,5* [0-6,5] DD: 11,7 (8,3) | 49 (15,2) |  |
| **Jacobsen et al., 2021 meta-regression** | **Mixed 77,6% RRMS 9,2% PPMS 13,2% SPMS** | 76 | Female%: 68,4 Age: 41,8 (9,7) EDSS: *3,5* [2,57-4] DD: *5* [3,25-11,75] | 42,1 (12,8) |  |
| **Jakimovski et al., 2019 meta-regression** | **Mixed 62,12% RRMS 37,88% PPMS&SPMS inclusion 18-75ys** | 132 | Female%: 69,7 Age: 53,5 (11,3) Education: 14,9 (2,3) EDSS: *3* [2-6] DD: 20,4 (10,5) | 49,5 (12,5) |  |
| **Jamoussi et al., 2023 meta-regression** | **Mixed 77,14% RRMS 8,57% PPMS 14,28% SPMS** | 35 | Female%: 54,28 Age: 41,77 (11,11) EDSS: 3,37 (1,75) DD: 12,1 (9,36) | 34,88 (13,95) |  |
| **Kantorová et al., 2021 meta-regression** | **RRMS 100% RRMS early MS** | 36 | Female%: 55,56 Age: 34,7 (8,7) [21-54] EDSS: 3,3 (1,2) [1-5] DD: 6,6 (3,4) [0,83-13,5] | 41,1 (9,7) [20-56] |  |
| **Karpuz Seren et al., 2022 meta-regression** | **Mixed 88,6% RRMS 2,9% PPMS 8,6% SPMS** | 70 | Female%: 60 Age: 38,8 (9,6) EDSS: 2,2 (1,68) DD: 8,4 (7) | 31,06 (13,77) |  |
| **Kever et al., 2021, MEMCONNECT cohort meta-regression** | **RRMS 100% RRMS inclusion 18-65ys** | 62 | Female%: 80,65 Age: 39,8 (9,5) EDSS: *1* [0-1] DD (time since dg): 7,2 (6,6) | 52,13 (10,85) |  |
| **Kever et al., 2021, RADIEMS cohort meta-regression** | **Mixed 10,81% CIS 89,19% RRMS newly diagnosed MS, inclusion 20–50 ys** | 185 | Female%: 66,49 Age: 34,4 (7,5) EDSS: *1* [0-1,5] DD (time since dg): 2,1 (1,5) | 57,17 (10,78) |  |
| **Khaligf-Razavi et al., 2020 meta-regression, correlation** | **Mixed 91% RRMS 2% PPMS 7% SPMS** | 91 | Female%: 82 Age: 37,24 (10,2) Education: 14,21 (3,16) EDSS: 1,27 (1,8) DD: 6,8 | 41,04 (11,02) |  |
| **Khalil et al., 2017 meta-regression** | **RRMS 100% RRMS** | 70 | Age: 34,3 (9,4) EDSS: 2,3 (1,6) DD: 7,8 (1) | 29,5 (12) |  |
| **Khan et al., 2021 meta-regression, correlation** | **Mixed 12,16% CIS 58,1% RRMS 29,72% SPMS** | 74 | Female%: 66,21 Age: 36,75 (9,15) EDSS: 2 (2,38) DD (time since dg): 1,89 (4,38) | 39,09 (14,15) |  |
| **Khan et al., 2021 meta-regression** | **RRMS 100% RRMS** | 43 | Female%: 69,77 Age: 34,77 (8,8) EDSS: 0,93 (1,26) DD: 7,47 (3,43) | 42,84 (12,37) |  |
| **Khan et al., 2021 meta-regression** | **SPMS 100% SPMS** | 22 | Female%: 59 Age: 40,76 (9,72) EDSS: 4,64 (2,45) DD: 10,44 (4,93) | 30,71 (15,14) |  |
| **Khedr et al., 2022 meta-regression, correlation** | **RRMS 100% RRMS** | 43 | Female%: 69,8 Age: 30,53 (7,68) [16-47] Education: 10,16 (7,68) [16-47] EDSS: 3,65 (1,66) [1-7] DD: 4,74 (4,31) [0,5-19] | 29,07 (11,74) |  |
| **Labiano-Fontcuberta et al., 2014 meta-regression** | **Mixed 55,6% RRMS 42,8% progressive MS** | 63 | Female%: 62 Age: 47,2 (10,2) EDSS: *5* [1-8] DD (time since dg): 11,3 (7,1) | 39,5 (15,5) |  |
| **Lam et al., 2021 meta-regression** | **Mixed 60% RRMS 10,6% PPMS 29,4% SPMS** | 85 | Female%: 75,3 Age: 46,4 (10,1) EDSS: *3,5* [2,5-4] DD (time since dg): 5,7 [3-13,5] | 54,5 (10,4) |  |
| **Lebkuecher et al., 2021 meta-regression** | **Mixed 71,62% RRMS 6,75% PPMS 20,27% SPMS 1,35% PRMS** | 74 | Female%: 78,37 Age: 49,45 (9,5) Education: 15,76 (2,38) DD: 13,97 (9,03) | 44,11 (12,73) [11-68] |  |
| **Loitfelder et al., 2014 meta-regression** | **RRMS 100% RRMS** | 13 | Female%: 60 Age: 31,3 (10) Education: 14,88 (3,8) EDSS: *1,5* [0-3,5] DD: 2,55 [0,3-10,1] | 53 (18) |  |
| **López-Góngora et al., 2015 meta-regression** | **RRMS 100% RRMS** | 237 | Female%: 66,24 Age: 38,5 (10,2) Education: 13,1 (4,1) EDSS: *1,5* [0-6] DD: 7,4 (7,1) | 54,3 (13,4) |  |
| **Lopez‑Soley et al., 2023 meta-regression** | **Mixed 6% CIS 86% RRMS 2% PPMS 6% SPMS** | 185 | Female%: 71 Age: 43 (9,75) DD: *10,6* [0,1-41,7] | 52,9 (12,8) |  |
| **Louapre et al., 2016 meta-regression** | **Mixed 5,88% CIS 67,65% RRMS 26,47% SPMS inclusion 18-60ys** | 34 | Female%: 67,65 Age: 43 (9,3) EDSS: *2,5* [1-8] DD: 11 (7,1) | 56 (14) |  |
| **Mackay et al., 2021 correlation** | **Mixed 88,67% RRMS 1,88% PPMS 9,43% SPMS** | 53 | Female%: 69,81 Age: 44,23 (11,19) [20-62] EDSS: *2* [0-8] DD: 13,25 (8,41) [1-36] | nd |  |
| **Maltby et al., 2022 meta-regression, correlation** | **Mixed 66% RRMS 7% PPMS 27% SPMS** | 97 | Female%: 79 Age: 50,5 (12,7) [26-81] EDSS: 4 (2,2) [0-8] DD (time since first sy): 16,3 (11) [1,25-47] | 43,9 (13,9) |  |
| **Marinetto et al., 2023 meta-regression** | **Mixed 98,18% RRMS 1,82% PPMS inclusion 19-62ys** | 55 | Female%: 63,64 Age: 39,76 (11,25) 39 [19-62] Education: 13 (3,59) *13* [5-19] EDSS: 2,06 ˙(1,45) *1,5* [0-6,5] DD (time since first sy): 11,2 (9,24) [1-47] | 55 (14,3) *52* [31-109] |  |
| **Marstrand et al., 2019 meta-regression, correlation** | **RRMS 100% RRMS early MS, inclusion 18-59ys** | 65 | Female%: 63 Age: 37,2 (8,8) [19-56] Education: 15,2 (2,4) EDSS: 1,8 (1,2) [0-4] DD: 3,9 (2,7) [1-10] | 61 (10) |  |
| **Martí-Juan et al., 2023 (Amsterdam) meta-regression** | **Mixed (nd)** | 173 | Female%: 71,68 Age: 48,8 (11,3) EDSS: *3,5* [2,5-5,5] DD: 15,26 (8,7) | 51,21 (13,3) |  |
| **Martí-Juan et al., 2023 (Clinic) meta-regression** | **Mixed (nd)** | 58 | Female%: 72,41 Age: 48,81 (9,6) EDSS: *2,5* [1,5-3,9] DD: 19,54 (9,4) | 46,05 (13,4) |  |
| **Martí-Juan et al., 2023 (London) meta-regression** | **Mixed (nd)** | 43 | Female%: 62,79 Age: 34,43 (7,9) EDSS: 1,5 [1-2] DD: 0,41 (0,5) | 58,84 (9,9) |  |
| **Martí-Juan et al., 2023 (Mainz) meta-regression** | **Mixed (nd)** | 50 | Female%: 64 Age: 35,78 (11,6) EDSS: 1,5 [1,5-6,1] DD: 4,99 (6,6) | 53,12 (11,3) |  |
| **Martí-Juan et al., 2023 (Milan) meta-regression** | **Mixed (nd)** | 56 | Female%: 55,366 Age: 42,18 (9,7) EDSS: *3,75* [1,5-6,1] DD: 10,81 (9,8) | 50,95 (14,3) |  |
| **Martí-Juan et al., 2023 (Naples) meta-regression** | **Mixed (nd)** | 51 | Female%: 66,67 Age: 42,48 (12,9) EDSS: *4,5* [2,5-6] DD: 13,29 (9) | 41,29 (13,8) |  |
| **Martí-Juan et al., 2023 (Oslo) meta-regression** | **Mixed (nd)** | 58 | Female%: 70,69 Age: 40,59 (7,2) EDSS: *2* [1,5-2,9] DD: 10,09 (5,3) | 51,48 (9,5) |  |
| **Matías-Guiu et al., 2020 meta-regression** | **Mixed 76,78% RRMS 8,21% PPMS 15% SPMS inclusion 18-80ys** | 280 | Female%: 62,1 Age: 48,16 (10,68) Education: 13,68 (3,54) EDSS: *2,5* [1,5-4] DD: 14,03 (7,9) | 36,61 (13,35) |  |
| **Maubeuge et al., 2020 meta-regression** | **Mixed 32,52% RRMS 34,15% PPMS 33,33% SPMS inclusion 18-64ys** | 123 | Female%: 63,4 Age: 49,69 (9,41) EDSS: *4* [0-8] DD: 14,67 (9,09) | 50,31 (11,12) |  |
| **McKay et al., 2019 meta-regression** | **Mixed 97,6% relapsing-onset MS inclusion 18-55ys** | 5404 | Female%: 70,3 Age: *38,3* [31,4-45,2] DD: *5,2* [17-11] | 50,7 (12,1) |  |
| **Motl et al., 2013 meta-regression, correlation** | **Mixed 82,3% RRMS 13,54% progressive MS 4,17% unknown** | 96 | Female%: 80,2 Age: 52,7 (11,1) EDSS: *4,5* [3-6] DD: 11,8 (10) [1-43] | 44,6 (11) |  |
| **Neuhaus et al., 2018 meta-regression** | **Mixed 71,42% RRMS 22,85% PPMS 5,71% SPMS** | 35 | Female%: 60 Age: 43,8 (12,13) EDSS: *3* [0-6,5] DD: 12,9 (9,6) | 50,2 (16,5) |  |
| **Niino et al., 2014 meta-regression, correlation** | **Mixed 90,76% RRMS 1,08% PPMS 8,15% SPMS** | 184 | Female%: 73,36 Age: 39,3 (10,1) [18-71] Education: 4,92 (1,83) [0-9] EDSS: 2,38 (2,04) [0-8,5] DD: 9,3 (7,2) | 46,2 (15,3) [4-84] |  |
| **Nocentini et al., 2006 meta-regression, correlation** | **RRMS 100% RRMS** | 461 | Age: 35,9 (8,4) Education: 11,3 (3,6) EDSS: 2,6 (1,3) DD: *6,25* | 34,7 (11,7) |  |
| **Noori et al., 2019 meta-regression** | **RRMS 100% RRMS** | 50 | Female%: 84 Age: 32,36 (7,62) Education: 13,5 (2,88) EDSS: 0,86 (1,2) DD: 5,89 (4,06) | 45,62 (12,69) |  |
| **Ntoskou et al. 2018 meta-regression** | **RRMS 100% RRMS inclusion 18-55ys, EDSS<6,5** | 15 | Female%: 80 Age: 43,6 (9,74) Education: 11,67 (2,61) EDSS: *3,5* [1,5-4] DD: 9,2 (3,7) | 45,9 (7,5) |  |
| **Ntoskou et al. 2018 meta-regression** | **SPMS 100% SPMS** | 12 | Female%: 75 Age: 48,67 (8,07) Education: 11,75 (3,36) EDSS: *6,25* [6-7,5] DD: 18,42 (5,82) | 37,25 (6,5) |  |
| **O'Connell et al., 2015 meta-regression** | **Mixed 70% RRMS 2% PPMS 28% SPMS** | 67 | Female%: 73 Age: 43,9 (12,1) Education: 13,6 (2,7) EDSS: 1,8 (0,9) DD: 10,2 (8,4) | 46 (12,9) |  |
| **Oliveira et al., 2023 meta-regression** | **RRMS 100% RRMS inclusion 18-42ys, EDSS≤4,5** | 21 | Female%: 57,14 Age: 32 (7) [18-45] EDSS: 2,5 (1,1) [1-4,5] DD: 7,67 (6,42) [0,42-23] | 50,49 (10,7) |  |
| **Ozturk et al., 2021 correlation** | **Mixed (nd)** | 58 | Female%: 75,9 Age: 37,17 (10,25) DD: 5,85 (5,45) | nd |  |
| **Paolicelli et al., 2021 meta-regression** | **Mixed 6,25% CIS 93,75% RRMS early MS, inclusion 18-55ys, EDSS≤6** | 16 | Female%: 87,5 Age: *36,5* [32,75-40,25] Education: *13* [12,5-13,5] EDSS: *2* [1,5-2,5] DD: *0,95* [0,12-1,78] | 47,063 |  |
| **Parmar et al., 2022 meta-regression** | **Mixed 76,69% RRMS 23,31% SPMS** | 163 | Female%: 68 Age: 47,1 (11,3) EDSS: *3* [0-7,5] DD: 16,1 (9,4) | *47* [24-94] |  |
| **Parmar et al., 2022 meta-regression** | **RRMS 100% RRMS** | 125 | Female%: 72 Age: 44,7 (10,9) EDSS: *2,5* [0-6,5] DD: 14,5 (10,9) | *47* [24-94] |  |
| **Parmar et al., 2022 meta-regression** | **SPMS 100% SPMS** | 38 | Female%: 55,26 Age: 55,1 (8,8) EDSS: *5,25* [3-7,5] DD: 21,5 (9,7) | *38* [27-69] |  |
| **Parmenter et al., 2007 meta-regression, correlation** | **Mixed 70% RRMS 30% SPMS** | 100 | Female%: 78 Age: 44,61 (8,39) Education: 14,44 (2) EDSS: 2,5 [0-7,5] | 47,66 (14,71) |  |
| **Patel et al., 2018 meta-regression** | **Mixed 2% CIS 86% RRMS 4% PPMS 8% SPMS inclusion 21-60ys** | 50 | Female%: 78 Age: 44,66 (11,08) Education: 15,14 (2,36) EDSS: *2* [0-6,5] DD: 12,13 (8,11) | 47,22 (11,96) |  |
| **Pavisian et al., 2019 meta-regression** | **RRMS 100% RRMS inclusion 18-60ys** | 33 | Female%: 70 Age: 41,42 (9,89) Education: 14,71 (2,13) EDSS: *2* [0-6] | 41,06 (10,37) |  |
| **Pérez-Miralles et al., 2020 meta-regression** | **PPMS 100% PPMS** | 43 | Female%: 34,9 Age: 55,7 (9,5) EDSS: 5,1 (1,6) DD (time since dg): 4,8 (5,4) | 29,2 (12,9) |  |
| **Pinter et al., 2020 meta-regression** | **Mixed 28,6% CIS 65,1% RRMS 6,3% SPMS** | 63 | Female%: 67 Age: 36,85 (9,95) Education: *13* [15-11] EDSS: *2* [1-3] DD: *5* [0,5-9,5] | 48,48 (12,84 |  |
| **Podda et al., 2021 meta-regression** | **Mixed 45,3% RRMS 12,6% PPMS 42,1% SPMS** | 872 | Female%: 65,2 Age: 54,1 (12,6) [19-87] Education: 11,6 (4) EDSS: 5 (2) DD: 19,3 (12,3) | 35,3 (15,1) *35,5* [24,5-46] |  |
| **Pokryszko-Dragan et al., 2018 meta-regression** | **RRMS 100% RRMS** | 50 | Female%: 74 Age: 36,4 [20-56] EDSS: 2,7 [1-6] DD: 8,2 [2-20] | 49,8 (13,9) |  |
| **Povolo et al., 2019 meta-regression, correlation** | **Mixed 81% RRMS** | 158 | Female%: 65,2 Age: 43,06 (9,96) Education: 14,1 (2,2) EDSS: *2* [0-7] DD: 7,82 (7,81) | 54,06 (13,01) |  |
| **Printza et al., 2022 meta-regression** | **Mixed 72,17% RRMS 13,9% PPMS 13,9% SPMS** | 115 | Female%: 65,2 Age: 38,69 (12,19) [16-68] EDSS: 3,7 (2,02) DD (time since dg): 7,74 (7,8) DD (time since first sy): 10,08 (8,47) | 45,07 (14,24) |  |
| **Quinn et al., 2020 meta-regression** | **Mixed 24% RRMS 19% PPMS 53% SPMS 2% BMS 2% unknown** | 100 | Female%: 66 Age: 52,6 (10,78) [29-78] EDSS: 5,3 (1,2) *6* [4-8] [3-6,5] DD (time since dg): 14,2 (9,5) *14* [7,2-20,8] | 32,8 (12,3) |  |
| **Radetz et al., 2021 meta-regression** | **RRMS 100% RRMS** | 50 | Female%: 62 Age: 35,3 (11,1) EDSS: *1* [1,625-0,375] DD: 4,17 (4,92) | 53,32 (10,5) |  |
| **Rasche et al., 2018 meta-regression, correlation** | **RRMS 100% RRMS** | 59 | Female%: 55,93 Age: 37,5 (9,7) EDSS: *1,5* [0-3] DD (time since first sy): 4,1 (2,7) | 56,6 (11,7) |  |
| **Reia et al., 2021 meta-regression, correlation** | **Mixed 75,36% RRMS 2,9% PPMS 21,74% SPMS** | 69 | Female%: 71 Age: 48,63 (11,8) Education: 12,5 (3,77) EDSS: *3,5* [1-8] DD (time since dg): 12,99 (8,11) | 41,17 (11,59) |  |
| **Riccardi et al., 2021 meta-regression** | **RRMS 100% RRMS** | 120 | Female%: 77,5 Age: 42,2 (10,1) [20-60] Education: 13,5 (3,9) [8-26] EDSS: *2* [1,5-3] [0-7] DD: 11,3 (9,5) [0,1-39] | 53,4 (14,6) [17-109] |  |
| **Roberg et al., 2015 (Study1) meta-regression** | **Mixed 72% RRMS 9,32% PPMS 18,6% SPMS** | 118 | Female%: 79,66 Age: 47 (10,5) DD: 9,67 (8,55) | 50,34 (12,02) |  |
| **Roberg et al., 2015 (Study2) meta-regression** | **Mixed 89,87% RRMS 10,12% SPMS** | 79 | Female%: 89,87 Age: 47,1 (10,79) EDSS: 2,67 (1,47) DD: 10,96 (8,3) | 51,13 (12,63) |  |
| **Roberg et al., 2015 (Study3) meta-regression** | **Mixed 51,51% RRMS 48,48% SPMS** | 66 | Female%: 78,78 Age: 51,38 (9,9) DD: 13,67 (10,4) | 49,6 (13,45) |  |
| **Rodgers et al., 2013 meta-regression** | **Mixed 48% RRMS 52% SPMS** | 50 | Female%: 60 Age: 49 (8,88) Education: 14,76 (2,48) EDSS: 4 (1,9) DD: 13,3 (8) | 47,44 (15,34) |  |
| **Rosenstein et al., 2023 meta-regression** | **RRMS 100% RRMS early MS** | 77 | Female%: 70,1 Age: *32* [27,5-43] EDSS: *2* [0,5-3] DD (time since first sy): 9,6 (5,4) | 55,2 (11,4) |  |
| **Roura et al., 2021 meta-regression** | **Mixed 4,1% CIS 95,89% RRMS** | 146 | Female%: 70,54 Age: 40,1 (9,19) EDSS: 1,63 (0,87) *1,5* [1-2] DD: 8,12 (6,89) | 54,2 (13,6) |  |
| **Roy et al., 2015 meta-regression** | **Mixed 66,6% RRMS 33,3% SPMS** | 87 | Female%: 70,1 Age: 46,4 (9,4) Education: 14,6 (2,3) EDSS: *3,5* [0-6,5] DD: 11,5 (8,5) | 52,5 (12,6) |  |
| **Ruet et al., 2013 meta-regression** | **Mixed 72,1% RRMS 27,9% progressive MS** | 43 | Female%: 69,77 Age: 48,4 (9,6) EDSS: *3,5* [0-8,5] DD: 12,8 (3,1) | 46,7 (14,2) |  |
| **Ruet et al., 2013 meta-regression** | **RRMS 100% RRMS** | 31 | Female%: 70,97 Age: 45,4 (8,3) EDSS: *2,5* [0-6] DD: 12,4 (2,2) | 50,6 (12,8) |  |
| **Sandi et al., 2015 meta-regression** | **RRMS 100% RRMS** | 65 | Female%: 75,38 Age: 41,9 (8,9) EDSS: 2,5 (1,8) DD: 11,1 (7,6) | 44,31 (11,76) |  |
| **Sandroff et al., 2018 meta-regression, correlation** | **Mixed 77% RRMS 23% progressive MS inclusion 18-64ys** | 62 | Female%: 72,6 Age: 52,6 (7,1) Education: 16,3 (2,1) EDSS: *4,5* [1,5-6,5] DD: 13,4 (8,8) | 50,4 (12,8) |  |
| **Sandry et al., 2021 (Clinical Sample) meta-regression** | **Mixed 5,14% CIS 74,05% RRMS 7,57% PPMS 6,48% SPMS 6,76% unknown** | 370 | Female%: 70 Age: 44 (11,8) DD: *5,8* [1,9-13] | 50,6 (12,2) |  |
| **Sandry et al., 2021 (Community sample) meta-regression** | **Mixed 79,2% RRMS 5,7% PPMS 15,1% SPMS** | 106 | Female%: 82 Age: 49,2 (9,5) DD: *11* [7-17] | 49,3 (11,1) |  |
| **Sandry et al., 2021 (RADIEMS sample) meta-regression** | **Mixed 10,81% CIS 89,18% RRMS** | 185 | Female%: 66 Age: 34 (7,5) DD: *2* [0,88-3,3] | 57 (9,7) |  |
| **Saposnik et al., 2022 meta-regression** | **RRMS 100% RRMS early MS, EDSS 0-5,5** | 188 | Female%: 70,7 Age: 36,6 (9,5) EDSS: 1,4 (1) 1 [0-2] DD (time since dg): 1,2 (0,8) | 51,7 (14,7) |  |
| **Savini et al., 2019 meta-regression, correlation** | **Mixed 100% relapse-onset MS** | 68 | Female%: 64,7 Age: 46,7 (11) EDSS: *4,5* [1-8,5] DD: 16,7 (10,4) | 47,1 (12,5) |  |
| **Schiavi et al., 2020 meta-regression** | **RRMS 100% RRMS** | 24 | Female%: 50 Age: 42,2 (10,5) EDSS: *2* [0-6] DD: 10,39 (8,08) | 52,4 (11,13) |  |
| **Schmidt et al., 2018 correlation** | **RRMS 100% RRMS early MS, EDSS≤3** | 35 | Female%: 80 Age: 35,4 (8,8) Education: 15,4 (4,3) DD: 1,77 (0,975) | nd |  |
| **Shi et al., 2023 meta-regression** | **RRMS 100% RRMS inclusion 18-60ys** | 68 | Female%: 82,35 Age: 29,9 (9,3) Education: 14 (2,7) EDSS: 1,4 (1,5) DD: 3,9 (5,1) | 48,1 (14,6) |  |
| **Siddiqui et al., 2022 meta-regression** | **Mixed 60,65% RRMS 39,34% progressive MS** | 122 | Female%: 73 Age: 54,1 (11,5) Education: 14,7 (2,3) EDSS: *3,25* [1,25-5,25] DD: 21 (10,7) | 49,3 (14) |  |
| **Siddiqui et al., 2022 meta-regression** | **RRMS 100% RRMS** | 74 | Female%: 70 Age: 49,6 (11,5) Education: 14,8 (2,28) EDSS: *2* [1,1-2,9] DD: 16,8 (9,13) | 53,7 (12,4) |  |
| **Siepman et al., 2008 meta-regression** | **Mixed (nd) early MS, inclusion 18-55ys, EDSS≤6** | 101 | Female%: 70 Age: 37,5 (9,5) EDSS: *2,5* [0,5-3,5] DD (time since dg): 0,65 (0,54) *0,425* DD (time since first sy): 3,8 (4,6) *2,1* | 56,3 (13,2) |  |
| **Sirhan et al., 2018 meta-regression** | **Mixed 86,67% RRMS 13,33% progressive MS inclusion 18-45ys** | 30 | Female%: 50 Age: 38,8 (5,7) Education: 14,5 (2,5) EDSS: *3* [2-5] DD: 11,8 (6,8) | 31,7 (7,6) |  |
| **Skorve et al., 2020 meta-regression, correlation** | **RRMS 100% RRMS newly diagnosed MS** | 58 | Female%: 75,86 Age: 37,6 (10,6) EDSS: 1,35 *1,5* DD (time since dg): 1,2 (0,8) [0,2-2,7] DD (time since first sy): 1,9 (1,3) [0,3-5,3] | 54,84 (10,83) |  |
| **Souissi et al., 2021 meta-regression, correlation** | **Mixed 88,5% RRMS 3,84% PPMS 7,69% SPMS** | 104 | Female%: 75 Age: 33,3 (9,8) EDSS: 2,65 (2,06) DD: 7 (6,4) | 36 (13) |  |
| **Spedo et al., 2015 meta-regression, correlation** | **RRMS 100% RRMS** | 58 | Female%: 69 Age: 41,2 (12,2) [18-65] Education: 12,7 (5,2) [3-28] EDSS: 4,2 (2) *4,25* [0-7,5] DD: 8,3 (6,6) | 35,9 (16,1) [4-64] |  |
| **Strober et al., 2009 meta-regression** | **Mixed 1,5% CIS 72% RRMS 3% PPMS 22% SPMS 1,5% PRMS** | 65 | Female%: 80 Age: 45 (9,9) Education: 14 (2,4) DD: 10,2 (7,6) | 48,4 (10,1) |  |
| **Trufanov et al., 2021 meta-regression** | **RRMS 100% RRMS** | 40 | Female%: 80 Age: 31,75 (5,96) EDSS: 1,54 (0,46) DD: 2,33 (1,5) | 57,67 (9,39) |  |
| **Trufanov et al., 2021 meta-regression** | **SPMS 100% SPMS** | 28 | Female%: 75 Age: 33,33 (5,7) EDSS: 4,17 (0,85) DD: 5,56 (4,41) | 43 (8) |  |
| **Tsagkas et al., 2022 meta-regression** | **Mixed 77,8% RRMS 22,17% SPMS** | 230 | Female%: 69,1 Age: 44,5 (11,3) EDSS: 3,05 (1,63) [0-7,5] DD: 12,99 (9,18) | 46,86 (13,42) |  |
| **Tsagkas et al., 2022 meta-regression** | **RRMS 100% RRMS** | 179 | Female%: 73,7 Age: 41,36 (10,22) EDSS: 2,57 (1,36) [0-7,5] DD: 11,33 (8,29) | 48,42 (13,94) |  |
| **Tsagkas et al., 2022 meta-regression** | **SPMS 100% SPMS** | 51 | Female%: 52,9 Age: 55,29 (7,62) DD: 18,96 (9,74) | 41,3 (9) |  |
| **Valdés Cabrera et al., 2019 meta-regression** | **Mixed 39,28% RRMS 28,57% PPMS 32,14% SPMS** | 28 | Female%: 64,28 Age: 48 (12) [21-66] EDSS: 4,8 (1,7) [1,5-8,5] DD (time since dg): 13 (10) [1-34] | 52 (14) [23-89] |  |
| **Valdés Cabrera et al., 2019 meta-regression** | **RRMS 100% RRMS** | 11 | Female%: 81,8 Age: 40 (12) [21-58] EDSS: 3,5 (1,5) [1,5-6] DD (time since dg): 9 (8) [1-28] | 52 (9) [36-71] |  |
| **Valdés Cabrera et al., 2019 meta-regression** | **SPMS 100% SPMS** | 9 | Female%: 77,77 Age: 55 (7) [45-66] EDSS: 5,6 (1,5) [4-8,5] DD (time since dg): 22 (9) [2-34] | 57 (17) [34-89] |  |
| **Valdés Cabrera et al., 2019 meta-regression** | **PPMS 100% PPMS** | 8 | Female%: 0,25 Age: 54 (7) [41-65] EDSS: 5,8 (1) [4-7] DD (time since dg): 8 (6) [3-17] | 46 (13) [23-62] |  |
| **Van Laethem et al., 2022 meta-regression, correlation, multivariate regression model** | **Mixed 75,3% RRMS** | 275 | Female%: 64 Age: 52,5 [23-78] *53* EDSS: 4,6 *4,5* [0-9] DD: 18,9 *18* [2-49] | 47 *48* [15-74] |  |
| **Vanotti et al., 2017 meta-regression, correlation** | **Mixed 78% RRMS 4% PPMS 18% SPMS inclusion 18-60ys** | 50 | Female%: 74 Age: 43,42 (10,17) [19-60] Education: 14,86 (2,78) EDSS: 3,29˙(2,55) *2,25* [0-8] DD: 13,06 (9,08) [1-40] | 45,14 (16,07) [10-91] |  |
| **Vazquez-Marrufo et al., 2019 EDSS correlation only** | **RRMS 100% RRMS** | 20 | nd | nd |  |
| **Vinciguerra et al., 2020 meta-regression** | **RRMS 100% RRMS** | 60 | Female%: 76,7 Age: 43,1 (9,9) Education: 13,97 (3,3) EDSS: *1,5* [1-3] DD (time since first sy): 9,53 (7,43) | 46,8 (11,1) |  |
| **Walker et al., 2016 meta-regression** | **Mixed 77,2% RRMS 7% PPMS 15,8% SPMS inclusion 18-59ys** | 57 | Female%: 71,93 Age: 45,44 (9,93) Education: 14,55 (2,68) EDSS: 2,7 (1,85) DD: 10,11 (7,72) | 49,65 (10,78) |  |
| **Warlop et al., 2009 meta-regression, correlation** | **RRMS 100% RRMS inclusion 21-49ys, EDSS 0-7** | 15 | Female%: 100 Age: 37,6 [21-49] Education: 13,93 EDSS: 2,47 (2,17) [0-7] DD: 5,13 (3,38) [1-11] | 60,4 (14,52) |  |
| **Welton et al., 2020 meta-regression** | **Mixed 59,45% RRMS 40,54% SPMS** | 37 | Female%: 81 DD: 17 (10) | 41,91 (13,09) |  |
| **Wen et al., 2015 meta-regression** | **RRMS 100% RRMS** | 10 | Female%: 90 Age: 49,4 (10,4) [32-60] EDSS: 3,05 (1,7) [1-6,5] | 54,6 (10,9) [32-68] |  |
| **Wen et al., 2015 meta-regression** | **PPMS 100% PPMS** | 10 | Female%: 60 Age: 55,2 (10,2) [37-74] EDSS: 5,1 (1,3) [3,5-6,5] | 42,5 (14,5) [10-61] |  |
| **Wen et al., 2015 meta-regression** | **SPMS 100% SPMS** | 9 | Female%: 77,7 Age: 59,2 (9,3) [45-75] EDSS: 5,5 (1,1) [4-6,5] | 44 (15,2) [18-63] |  |
| **Wetter et al., 2016 meta-regression, correlation** | **Mixed (nd) inclusion 18-64ys** | 52 | Female%: 78,85 Age: 51 (8,4) [25-64] EDSS: *5,5* [4,08-6,92] [0-7,5] | 46,06 (12,16) [20-77] |  |
| **Wieder et al., 2013 meta-regression** | **RRMS 100% RRMS** | 89 | Female%: 60 Age: 42 (9) [25-62] EDSS: *2* [0-6] DD (time since dg): 8,08 (5,58) | 56 (12) [32-85] |  |
| **Yaldizli et al., 2013 meta-regression** | **Mixed 1,8% CIS 74,3% RRMS 4,4% PPMS 19,5% SPMS** | 113 | Female%: 66,4 Age: 47,8 (10,8) *48* [45,8-49,8] EDSS: 3,2 (1,7) *3* [3-3,6] DD (time since first sy): 20,5 (9,1) *19* [18,9-22,2] | 47,9 (13,7) |  |
| **Yigit et al., 2020 meta-regression, correlation** | **Mixed 95% RRMS 2% PPMS 3% SPMS** | 200 | Female%: 70 Age: 36,53 (9,73) Education: 11,98 (3,55) EDSS: 1,78 (1,58) DD: 9,17 (4,85) | 47,99 (12,15) |  |
| **Yoshii et al., 2015 meta-regression, correlation** | **RRMS 100% RRMS** | 34 | Female%: 76,47 Age: 42 (12) [20-74] EDSS: 2,2 (2,4) [0-8,5] DD: 8 (7,2) [1-32] | *50* [38,8-59,3] |  |
| **Yu et al., 2011 meta-regression** | **RRMS 100% RRMS** | 37 | Female%: 83,78 Age: 40,9 (10,1) EDSS: *2,25* [0-4] DD: 9,3 (9,5) | 54,5 (12) |  |
| **Zheng et al., 2023 meta-regression** | **Mixed 73% RRMS 27% progressive MS inclusion 18-64ys, EDSS<8** | 45 | Female%: 62 Age: 51,7 (7,8) [27-64] EDSS: *4,5* [3,25-5,75] DD: 14 (8,8) | 51,1 (15,4) [na-na] |  |
| **Zhu et al., 2022 meta-regression** | **RRMS 100% RRMS** | 48 | Female%: 64,58 Age: 33,1 (9,2) Education: 14,1 (2,9) EDSS: 2,2 (1,3) DD: 5,4 (5,2) | 42,6 (17,3) |  |
| nd: no data; dg: diagnosis; sy: symptom; DD: Disease Duration; EDSS: Expanded Disability Status Scale; ys: years; PMS: Progressive Multiple Sclerosis; PRMS: Progressive-Relapsing Multiple Sclerosis; RMS: Relapsing MS | | | | |  |
|  |  |  |  |  |  |
| **PPMS - Univariate meta-regressions** |  | | | |  |
| **Mixed MS - Univariate meta-regressions** |  |  |  |  |  |
| **RRMS - Univariate meta-regressions** |  |  |  |  |  |
| **RRMS - Correlations** |  |  |  |  |  |
| **Mixed MS - Univariate meta-regressions, correlations and multivariable regression models** |  |  |  |  |  |
| **RRMS - Univariate meta-regressions and correlations** |  |  |  |  |  |
| **Mixed MS - Correlations** |  |  |  |  |  |
| **SPMS - Univariate meta-regressions** |  |  |  |  |  |
| **Mixed MS - Univariate meta-regressions and correlations** |  |  |  |  |  |
